# CancerNet: a unified deep learning network for pan-cancer diagnostics

**DOI:** 10.1186/s12859-022-04783-y

**Published:** 2022-06-13

**Authors:** Steven Gore, Rajeev K. Azad

**Affiliations:** 1 Department of Biological Sciences and BioDiscovery Institute, University of North Texas, Denton, TX 76203 USA; 2 Department of Mathematics, University of North Texas, Denton, TX 76203 USA

**Keywords:** Cancer, Neural network, Deep learning, Metastatic cancer

## Abstract

**Background:**

Despite remarkable advances in cancer research, cancer remains one of the leading causes of death worldwide. Early detection of cancer and localization of the tissue of its origin are key to effective treatment. Here, we leverage technological advances in machine learning or artificial intelligence to design a novel framework for cancer diagnostics. Our proposed framework detects cancers and their tissues of origin using a unified model of cancers encompassing 33 cancers represented in The Cancer Genome Atlas (TCGA). Our model exploits the learned features of different cancers reflected in the respective dysregulated epigenomes, which arise early in carcinogenesis and differ remarkably between different cancer types or subtypes, thus holding a great promise in early cancer detection.

**Results:**

Our comprehensive assessment of the proposed model on the 33 different tissues of origin demonstrates its ability to detect and classify cancers to a high accuracy (> 99% overall F-measure). Furthermore, our model distinguishes cancers from pre-cancerous lesions to metastatic tumors and discriminates between hypomethylation changes due to age related epigenetic drift and true cancer.

**Conclusions:**

Beyond detection of primary cancers, our proposed computational model also robustly detects tissues of origin of secondary cancers, including metastatic cancers, second primary cancers, and cancers of unknown primaries. Our assessment revealed the ability of this model to characterize pre-cancer samples, a significant step forward in early cancer detection. Deployed broadly this model can deliver accurate diagnosis for a greatly expanded target patient population**.**

**Supplementary Information:**

The online version contains supplementary material available at 10.1186/s12859-022-04783-y.

## Introduction

Survival rates of cancer patients dramatically improve when diagnosed in early stages as tumors may not have spread yet. However, detection rates in early stages are inconsistent across cancers. As an example, ~ 63% of breast cancer cases are diagnosed in stage 1 while only ~ 17% lung cancer cases are diagnosed in the same stage (https://seer.cancer.gov/csr/1975_2017/). This is owing, in part, to the fact that diagnostic development has historically focused on detecting individual cancers. Many cancers are detected only when the symptoms appear, which most often occur in later stages. The development of pan-cancer diagnostics would enable detection of more cancer types, including rare cancers that are not typically the focus of individual biomarker research, thus dramatically improving the prognosis and survival of cancer patients. Such a tool would allow clinicians to diagnose more patients earlier and guide more informed treatment decisions. Additionally, successful application of such a tool to pre-symptomatic patients would necessitate further efforts to locate the tumor to a specific body site with greater resolution. Here, we present a unified cancer diagnostic capable of both, robust cancer diagnosis and tissue of origin detection, for 33 different cancers.

Approximately 60% of genes in humans are found in genomic regions dense with CpG dinucleotides called CpG islands which may be methylated [1]. The degree of methylation influences expression of downstream genomic regions. Tissue specific patterns of methylation arise through development and limit the possible changes to the cell state during development or carcinogenesis [2, 3]. Methylation has been shown to be significantly altered in many cancers making it promising as a pan-cancer biomarker, and furthermore, as patterns of alteration vary by cancer types or subtypes, methylation is being exploited to distinguish different cancer types or subtypes [4–9]. Methylation data have previously been used to successfully develop classifiers for individual cancer types and cancers derived from tissues with common developmental lineages [10–22].

The high dimensional, real value data obtained from high-throughput methylation arrays, such as those archived in The Cancer Genome Atlas (TCGA; https://www.cancer.gov/tcga), are well suited for use with machine learning classifiers, including neural network. Detection of tissues of origin of cancers can be cast as a supervised task within the realm of machine learning. Supervised methods may artificially separate samples based only on pre-defined classes; however, unsupervised methods may generate a latent space which can be leveraged for many downstream tasks while retaining the underlying structure of the data. Among neural network architectures, unsupervised methods have seen growing use in biological data analysis, particularly for dimensionality reduction with high degrees of success [23–27].

Briefly, a class of unsupervised methods attempts to regenerate realistic samples from some low dimensional representation [28, 29]. Variational autoencoders (VAEs) [28], an unsupervised method, have been used as a basis for downstream regression or classification in a host of applications, including methylation or transcriptional data analysis. This has been done by passing the latent mapping of a sample to a classifier such as a support vector machine [27]. However, this does not allow for features in the latent space to be modified to improve the classification task. The unsupervised and supervised tasks may be used to constrain each other, resulting in an unsupervised latent space that retains the natural distribution of the data but is optimized for the classification task.

Here we propose a model where both the generative (unsupervised) and the classification (supervised) trainings take place at the same time. This hybrid generator/classifier architecture enables learning of discriminative features intrinsic to input data in tandem with producing a robust classifier. Tuned for and trained on cancer tissues of origin and normal/non-cancerous tissues, our proposed neural network, CancerNet, is currently capable of detecting 33 different cancers. CancerNet was assessed on multiple independent datasets including samples that were not used in training, and metastatic and early cancer samples.

## Materials and methods

### Methylation data

Illumina 450 K methylation array data were downloaded from The Cancer Genome Atlas (TCGA) GDC portal for all cancer types. Metastatic and recurrent samples were removed. This resulted in total 13,325 samples. Each sample was labeled by its tissue of origin and TCGA cancer type designation. Rather than creating a distinct class for each normal tissue, all samples that were from non-cancerous tissues were included in the normal class. This was done due to the extremely low numbers of normal samples available for some tissue types. Additional validation sample sets were downloaded from NCBI GEO (https://www.ncbi.nlm.nih.gov/geo/). Details of specific GEO datasets used are provided in Additional file 1: Table S1.

### Data preparation

We relied on the CpG density clustering approach implemented in CancerLocator to process the methylation data before inputting to CancerNet [16]. CpGs that were not assigned to CpG islands were removed. The remaining methylation data were scanned for Illumina 450 K probes that map to within 100 bp of each other, which were then concatenated. These clusters were then filtered to eliminate those with 3 CpGs or less [16]. The beta values for the resulting clusters were then averaged. This resulted in 24,565 clusters that map to CpG islands. These average beta values were used as input to CancerNet. The dataset was then randomly split into training/test/validation sets with 80% in training set and 10% each in the test and validation sets. We ensured that the training set did not include more than one sample per patient by removing one of any matched pairs present in the dataset and replacing it with a random sample from the same class.

### Performance assessment

Held-out test data from TCGA and GEO datasets were used to assess CancerNet’s performance measured in terms of recall, precision, and F-measure. For a specific class (e.g. a cancer tissue of origin or normal), recall defines the fraction of samples belonging to this class that are correctly identified by a classification method. Precision is the fraction of predictions for this class that are correct, and F-measure is the harmonic mean of recall and precision. Unless otherwise noted, the F-measure presented in this work is weighted F-measure due to large class imbalance among tumor classes. Weighted F-measure is the weighted average of F-measure values with weight proportional to the number of true instances for each class. The F-measure function in the scikit learn python library (https://scikit-learn.org/stable/index.html) was used to calculate this.

### Neural network

**Fig. 1:**
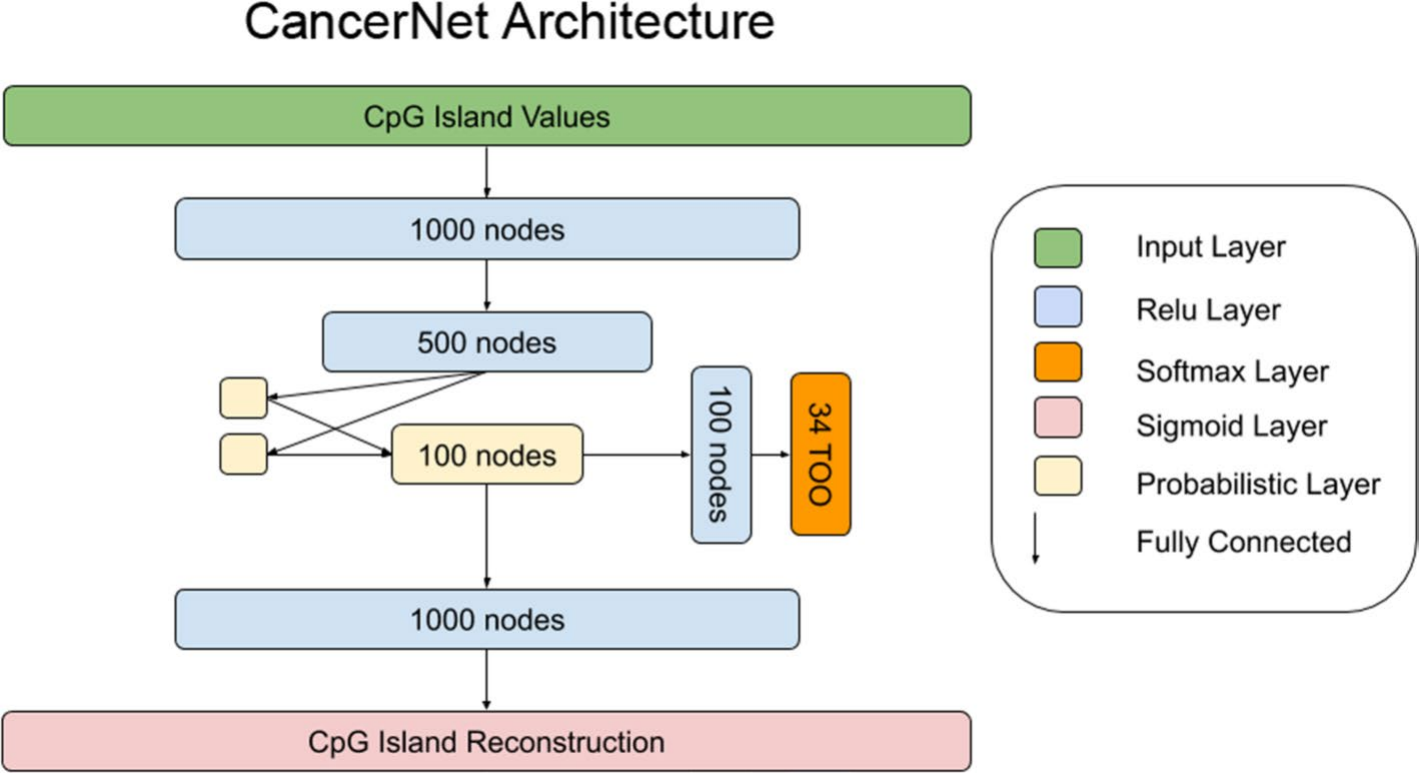
The CancerNet architecture. Methylation data are input to the encoder. The encoder is composed of two dense feedforward layers using the Relu activation function. Output of the encoder is passed to the probabilistic layer, which passes its output to the classifier and generator/decoder. The classifier is two dense feedforward layers, the first with the ReLu activation function and the second with the softmax activation function. The decoder is two dense feedforward layers, the first using the Relu activation and the second using the sigmoid activation

The CancerNet program was written in Python using the keras package (version 2.0.8) [30] with a tensorflow (version 1.12.0) [31] backbone. The neural network architecture of CancerNet consists of an encoder, decoder and classifier (Fig. 1). The encoder has an input layer of 24,565 nodes, which is fully connected to a dense layer of 1,000 nodes that uses a relu nonlinearity and two dense activation free layers that are passed to a probabilistic layer, also called the latent layer, characteristic of a VAE architecture with 100 nodes. The decoder has a single dense layer of 1000 nodes that uses a relu activation and is fully connected to an output layer of 24,565 nodes that uses a sigmoid activation. The classifier takes the latent layer as an input to a dense 100 node layer that uses a relu activation and is fully connected to the classifier output layer that has 34 nodes and uses a softmax activation (Fig. 1). CancerNet was trained using the Adam optimizer with a learning rate of 0.001. All layers were randomly initialized and then trained until convergence. Early stopping was used to limit training time and prevent overfitting and was limited to 50 epochs without validation accuracy improvement. The final loss of the network was the sum of the VAE loss and the categorical cross-entropy loss, which are applied to the generative output and the classification output respectively.

VAE loss is composed of two terms. The first term quantifies the divergence between the output of the generator and the input to the model using categorical cross-entropy. The second term is used to enforce gaussian distributions in the latent layer by calculating the Kullbeck–Leibler divergence of the encoders’ distribution and a standard normal. The VAE loss beta term can be used to create a disentangled VAE. When beta is greater than one, features are forced to disentangle and become easier to interpret. Beta is set to 1 in CancerNet.

Cross-entropy is applied to the classifier output to estimate a loss based on the difference between the classifier output and the class labels. This is distinct from the cross-entropy for quantifying the VAE loss based on the difference between the generative output and the sample itself. Weights of 0.01 and 1 are applied to the VAE loss and classifier loss, respectively. The generator and classification losses together enforce the latent space representation of samples to preserve information about samples’ natural distribution while also creating an easily classifiable distribution of samples. In doing so the latent space acts as a prior in the classifier.

### Prevention of leakage

Leakage is a phenomenon in machine learning where information about the task is inadvertently added to the data on which the task is being performed [32]. This can lead to very brittle models or even completely useless models when used outside the test and training data. Tasks such as normalizing datasets prior to splitting into training/testing/validation sets can introduce information present in the test and validation sets into the trained model, thus artificially inflating the performance of the model in validation and test phases [32]. The beta values of the Illumina 450 k array were normalized on a sample by sample basis and bounded in the range [0, 1], preventing information from crossing among samples. For CpG feature assignment and a list of samples used in test, train and validation sets please see Additional File 2. The validation set is then used as a sanity check to confirm the model performance on unseen data. We also demonstrate further that the model is robust by using independent datasets retrieved from GEO.

### CancerNet software

CancerNet is an open source software. CancerNet and associated datasets have been made available at the Open Science Framework (OSF) site: https://osf.io/j6gcv/?view_only=d18c6831b9e24002937ab1028fc0d418.

## Results

### Model performance

CancerNet’s parameters were learnt from training data obtained from The Cancer Genome Atlas (TCGA) for 33 different cancers and a normal class. The cancers investigated were Adrenocortical carcinoma (ACC), Bladder urothelial carcinoma (BLCA), Breast invasive carcinoma (BRCA), Cervical squamous cell carcinoma and endocervical adenocarcinoma (CESC), Cholangiocarcinoma (CHOL), Colon adenocarcinoma (COAD), Lymphoid neoplasm diffuse large B-cell lymphoma (DLBC), Esophageal carcinoma (ESCA), Glioblastoma multiforme (GBM), Head and Neck squamous cell carcinoma (HNSC), Kidney chromophobe (KICH), Kidney renal clear cell carcinoma (KIRC), Kidney renal papillary cell carcinoma (KIRP), Acute myeloid leukemia (LAML), Brain lower grade glioma (LGG), Liver hepatocellular carcinoma (LIHC), Lung adenocarcinoma (LUAD), Lung squamous cell carcinoma (LUSC), Mesothelioma (MESO), Ovarian serous cystadenocarcinoma (OV), Pancreatic adenocarcinoma (PAAD), Pheochromocytoma and paraganglioma (PCPG), Prostate adenocarcinoma (PRAD), Rectum adenocarcinoma (READ), Sarcoma (SARC), Skin cutaneous melanoma (SKCM), Stomach adenocarcinoma (STAD), Testicular germ cell tumors (TGCT), Thyroid carcinoma (THCA), Thymoma (THYM), Uterine corpus endometrial carcinoma (UCEC), Uterine carcinosarcoma (UCS), Uveal melanoma (UVM).

**Fig. 2:**
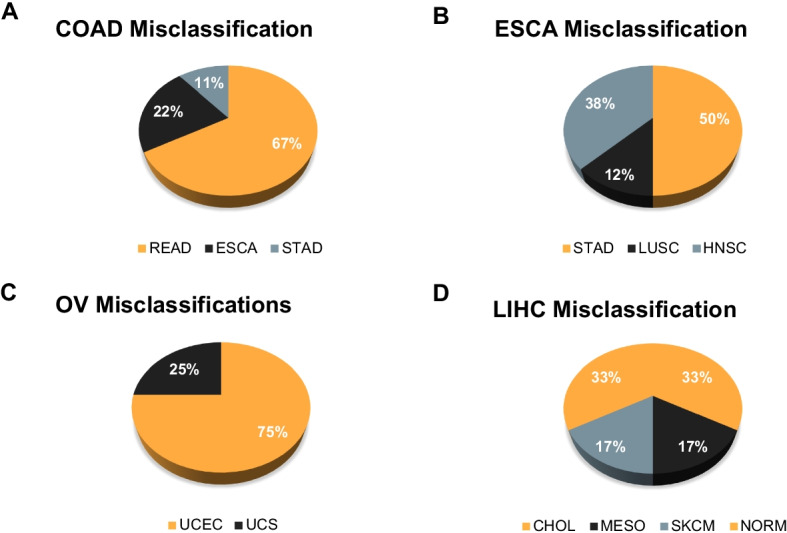
Misclassification rates for 4 cancer types selected to illustrate trends observed in CancerNet. **A** COAD misclassifies primarily to READ with fewer misclassifications in ESCA and STAD. **B** ESCA misclassifies to HNSC, LUSC and STAD. Lung misclassifications occur often among some sample types. **C** OV samples misclassify as the two uterine cancer types considered in CancerNet: UCEC and UCS. **D** LIHC misclassifies as CHOL, MESO, SKCM and NORM. Refer to Abbreviations for cancer types indicated

**Fig. 3:**
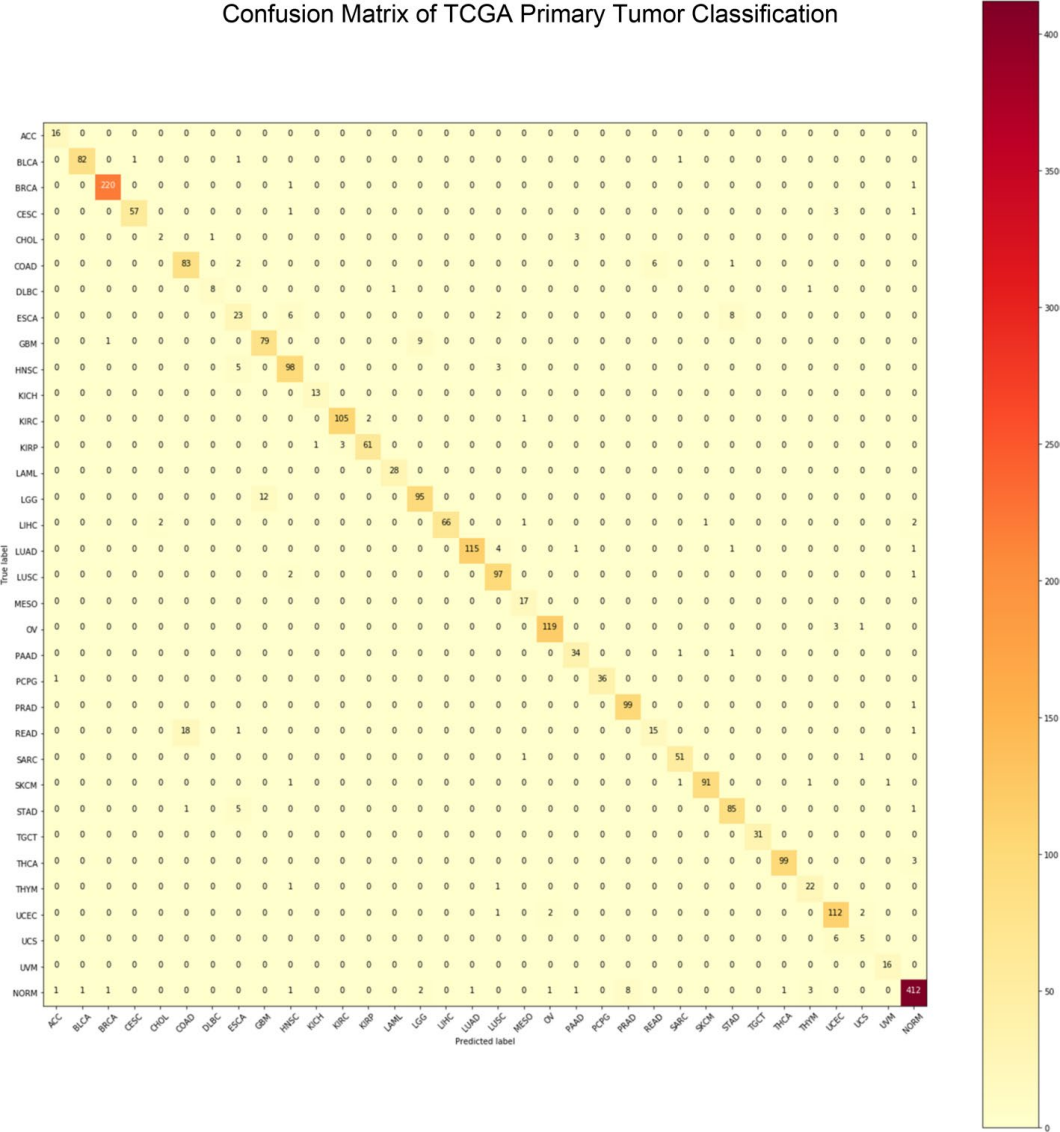
Confusion matrix of TCGA primary tumor classification. Primary tumors across 33 TCGA cancer types were classified. The correct class is shown by the Y-axis and the predicted class is shown by the X-axis (refer to Abbreviations for different cancers indicated on the X-axis; normal is abbreviated NORM)

**Fig. 4:**
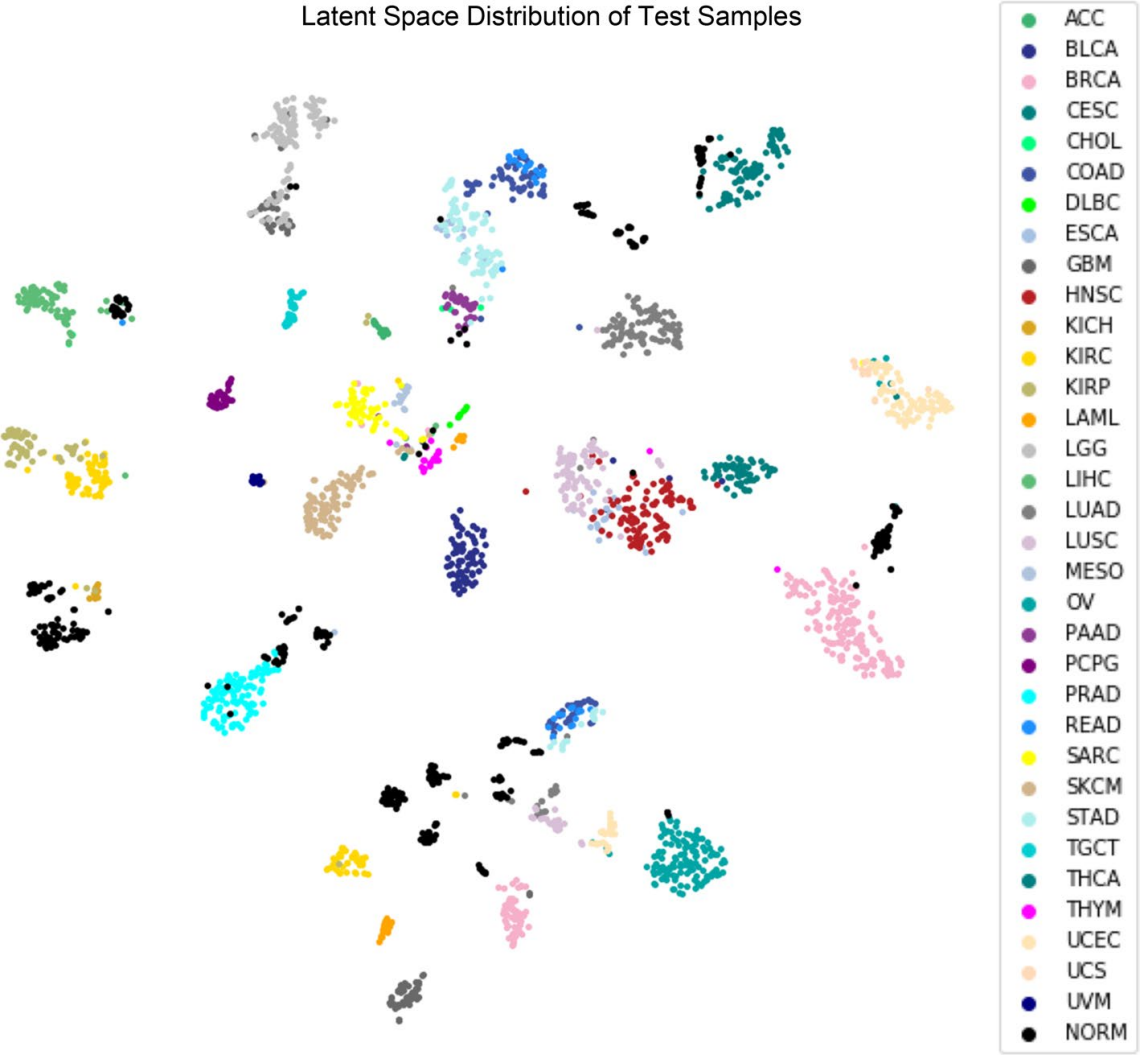
Visualization of test samples in the latent space. T-SNE was used to reduce the latent space dimension from 100 to 2. Samples originating from the same tissue form cluster(s) and are close to sample groups of similar tissues. For abbreviations, refer to the full abbreviation list. Normal samples are abbreviated NORM and are displayed in gray

The overall performance of CancerNet, as quantified through F-measure (see Methods), is ~ 99.6% (Table 1). Many of the misclassifications occurred among cancers from the same or similar organs and tissue classes that share developmental lineages (Fig. 2, Additional file 1: Fig. S1–S28). Where this did not hold true, we found a pattern of misclassification among adenocarcinomas and squamous carcinomas (Fig. 3, Additional file 1: Fig. S1–S28). Examination of the latent space (Fig. 4), along with misclassification rates, shows that misclassifications occurred among closely neighboring classes (Fig. 2) or for individual samples of a class that are singletons and very far from the rest of the class (Fig. 4, Additional file 1: Fig. S29–S63).

**Table 1:** CancerNet’s performance in detecting the tissue of origin of 33 cancers

Class	Precision	Recall	F1
ACC	0.99925	0.999249	0.999227
BLCA	0.998525	0.998498	0.998507
BRCA	0.998498	0.998498	0.998498
CESC	0.997868	0.997748	0.997784
CHOL	0.998248	0.997748	0.997973
COAD	0.98915	0.989489	0.989229
DLBC	0.998949	0.998874	0.998903
ESCA	0.989043	0.988739	0.988885
GBM	0.991661	0.991742	0.991697
HNSC	0.991985	0.992117	0.99203
KICH	0.999625	0.999625	0.999618
KIRC	0.997748	0.997748	0.997748
KIRP	0.997793	0.997748	0.997765
LAML	0.999625	0.999625	0.999621
LGG	0.991407	0.991366	0.991386
LIHC	0.997935	0.997748	0.997795
LUAD	0.997125	0.996997	0.997033
LUSC	0.994668	0.994745	0.994648
MESO	0.998875	0.998874	0.998829
OV	0.997384	0.997372	0.997377
PAAD	0.997312	0.997372	0.997321
PCPG	0.999635	0.999625	0.999627
PRAD	0.996604	0.996622	0.996567
READ	0.993231	0.99024	0.991447
SARC	0.998109	0.998123	0.998115
SKCM	0.99817	0.998123	0.998138
STAD	0.993135	0.993243	0.993174
TGCT	1	1	1
THCA	0.99852	0.998498	0.998506
THYM	0.997281	0.997372	0.997296
UCEC	0.993519	0.993619	0.99353
UCS	0.996655	0.996246	0.996433
UVM	0.999625	0.999625	0.999619
NORM	0.987758	0.987613	0.987665

A normal class (NORM) is also included. The performance was assessed using the accuracy metrics precision, recall and F1-measure

ACC—Adrenocortical carcinoma, BLCA—Bladder urothelial carcinoma, BRCA—Breast invasive carcinoma, CESC—Cervical squamous cell carcinoma and endocervical adenocarcinoma, CHOL—Cholangiocarcinoma, COAD—Colon adenocarcinoma, DLBC—Lymphoid neoplasm diffuse large B-cell lymphoma, ESCA—Esophageal carcinoma, GBM—Glioblastoma multiforme, HNSC—Head and neck squamous cell carcinoma, KICH—Kidney chromophobe, KIRC—Kidney renal clear cell carcinoma, KIRP—Kidney renal papillary cell carcinoma, LAML—Acute myeloid leukemia, LGG—Brain lower grade glioma, LIHC—Liver hepatocellular carcinoma, LUAD—Lung adenocarcinoma, LUSC—Lung squamous cell carcinoma, MESO—Mesothelioma, OV—Ovarian serous cystadenocarcinoma, PAAD—Pancreatic adenocarcinoma, PCPG—Pheochromocytoma and paraganglioma, PRAD—Prostate adenocarcinoma, READ—Rectum adenocarcinoma, SARC—Sarcoma, SKCM—Skin cutaneous melanoma, STAD—Stomach adenocarcinoma, TGCT—Testicular germ cell tumors, THCA—Thyroid carcinoma, THYM—Thymoma, UCEC—Uterine corpus endometrial carcinoma, UCS—Uterine carcinosarcoma, UVM—Uveal melanoma, NORM—Normal (non-cancer)

### Latent space evaluation

We confirmed that the latent space of CancerNet maintains the natural distribution of the sample data by comparing it to the latent space generated through a multi-omic clustering algorithm in a flagship paper from TCGA consortium [17]. The latent space of CancerNet shows high concordance with the latent space of TCGA data presented in Hoadley et al. study [17] (Fig. 4). Similar to this study, we observed clustering of the samples by tissue of origin and position in a specific organ in the CancerNet’s latent space (Fig. 4). Similar distributions of various subtypes of cancers were also observed. These observations suggest that CancerNet’s latent space maintains the natural distribution of the sample data. Note that Hoadley et al. used a highly curated set of methylation sites, devoid of any tissue specific promoter sites and chosen based on hypomethylation status, in order to perform unsupervised clustering of methylation data samples to establish that cancer type specific signatures are present in the tumor samples [17]. In contrast, CancerNet obtains similar results but with far less preprocessing of the data and in a manner that facilitates integration with other data types such as those used in the Hoadley et al. study (e.g. mRNA, aneuploidy, miRNA, and RPPA) by way of a latent space that is vector encoded.

**Fig. 5:**
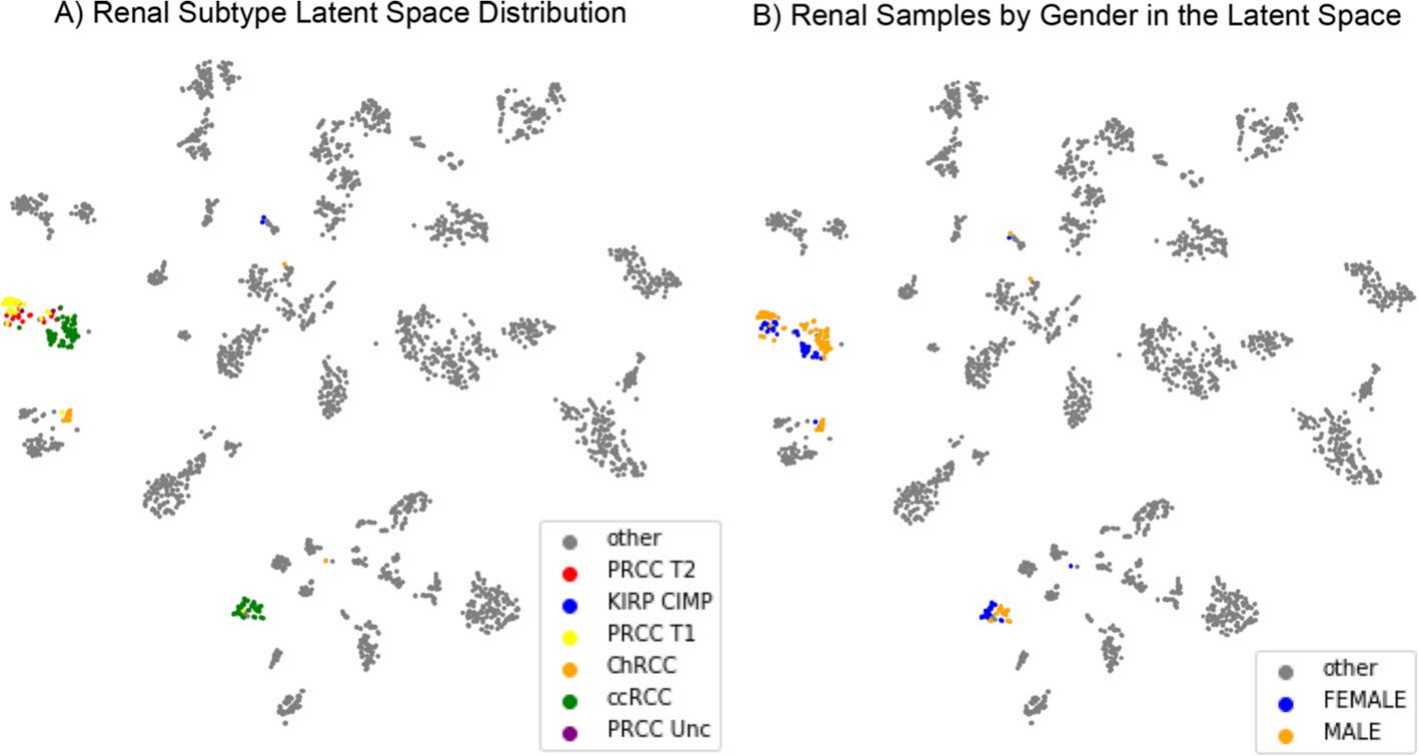
Renal subtype latent space distribution. **A** Samples representing different renal subtypes, as determined by the TCGA analysis of renal cancers, are mapped onto the latent space. Clear separation of subtypes PRCC T1 and T2 and ChRCC indicates that the neural network has learned features for discriminating between these renal subtypes. **B** Separation of renal samples in the latent space by gender

Renal cancer samples (KIRC, KICH, and KIRP) were apportioned into 3 clusters in the latent space (Fig. 4), very similar to those described in Hoadley et al. study [17]. The largest cluster consists of two distinct sub-clusters connected by a streak (Fig. 5A); while one sub-cluster is primarily composed of clear cell renal cell carcinoma (ccRCC) samples, the other is populated with papillary renal clear cell type1 (PRCC T1) samples with type 2 (PRCC T2) connecting these two (Fig. 5A). The remaining clusters are primarily composed of ccRCC and chromophobe renal cell carcinoma (chRCC) samples, respectively (Fig. 5A); chRCC is a rare subtype of renal cell carcinoma (RCC) found in only 5% of all renal cancer patients with a distinct etiology [33]. The presence of this RCC subtype as a distinct cluster in the latent space is encouraging as it could indicate the presence of detectable and therapeutically important features in the network. Among renal cancers, a distinct separation of samples by gender was also observed in the latent space (Fig. 5B).

**Fig. 6:**
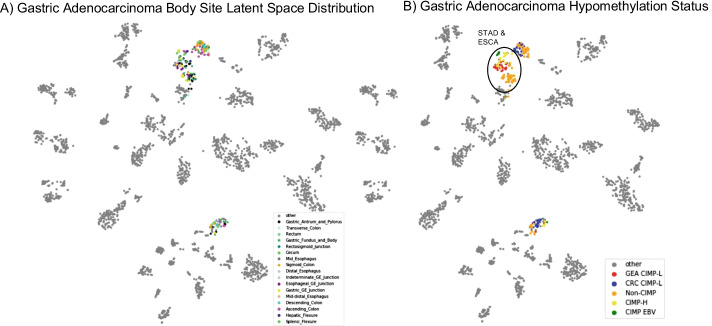
Gastric adenocarcinoma latent space distribution. Samples representing different gastric adenocarcinomas cluster in the latent space by **A** body site of tumor and **B** hypomethylation status

**Fig. 7:**
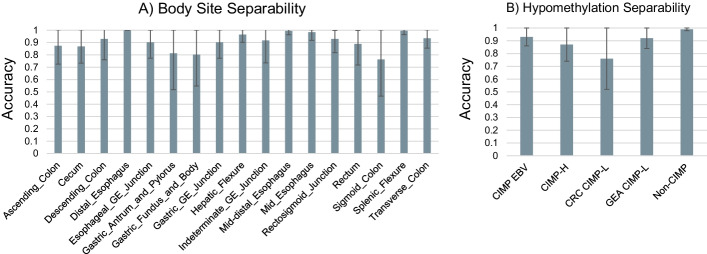
The tenfold cross-validation accuracy of a binary SVM with a linear kernel trained on the 100-dimensional latent space for each body site (**A**) and methylation status (**B**) for gastric adenocarcinoma. The linear kernel is used to test the separability of each in the full 100-dimensional latent space. The high performance of these models indicates that the body sites and methylation statuses are not overlapping in the higher dimensional latent space even though it may appear so in lower dimension representations (Fig. 6)

Gastrointestinal adenocarcinoma samples also arrange in a similar way as in the latent space of Hoadley et al. study [17]. Esophageal samples are split among the larger gastrointestinal cluster and a cluster of HNSC in the latent space (Fig. 4). Gastrointestinal adenocarcinomas show strong organ site signatures (Fig. 6A) and are best explained by hypomethylation status (Fig. 6B). Groupings correspond to CpG island methylator phenotype (CIMP) status as described by Ang et al. [34]. Non-CIMP separates from CIMP-high (CIMP-H) and CIMP-low (CIMP-L) (Fig. 6B). Stomach adenocarcinoma (STAD) and Esophageal carcinoma (ESCA) group together with CIMP-H and gastroesophogeal (GEA) CIMP-L status. Epstein-Barr virus (EBV) positive samples form their own cluster. Similarly, molecular subtypes follow the same pattern as in Hoadley et al. study [17] (Fig. 6B). Note that the latent space has been projected from 100 dimensions onto a 2-dimensional plane for visualization purposes (Fig. 6). To verify that distinct clusters do form by body sites and hypomethylation status in gastrointestinal adenocarcinomas, we trained a linear SVM on these classes. These models achieve high accuracy (tenfold cross-validation accuracy, Fig. 7), demonstrating the robust separability of the gastrointestinal adenocarcinoma samples by these classes in the latent space.

**Fig. 8:**
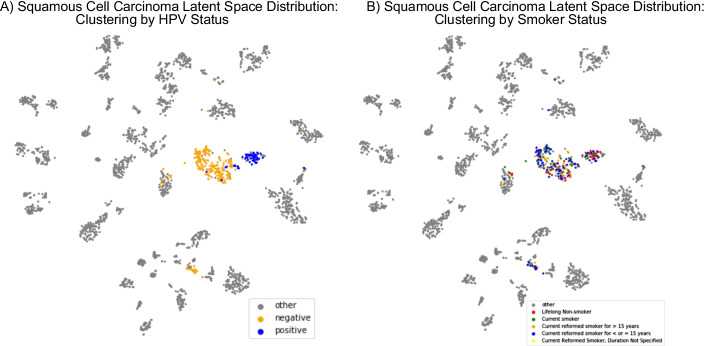
Squamous cell carcinoma latent space distribution. Squamous cell carcinoma samples tend to cluster in the latent space by their tissues of origin and by **A** HPV status but not by **B** smoker status

Squamous cell carcinoma samples (CESC, ESCA, HNSC, and LUSC) segregate by human papillomavirus (HPV) status in the latent space (Fig. 8A), which is in concordance with Campbell et al. study [35]. However, CancerNet did not show sensitivity to smoking status (Fig. 8B).

### Assessment on metastatic cancers, precancerous lesions, and age-related methylation drift

When trained on a narrow range of data neural networks may catastrophically fail on unseen conditions; these models are said to be brittle. To assess how brittle CancerNet is, we evaluated across 3 “untrained” conditions: metastatic tumors, precancerous lesions, and age stratified data. Our results demonstrate that CancerNet performs well across all stages of cancer and is robust to age related epigenetic drift.

#### Metastasis and precancerous lesions

Metastatic cancer is the cause of death in 66% of solid tumor cases [36]. Identification of a second cancer occurrence as a metastatic or second primary tumor is important to inform treatment. In 3–5% of all cases, cancers of unknown primaries (CUPs) are also found [37]; these tumors arise as the metastasis of previously undiscovered primary tumors and are the fourth most deadly cancer [37, 38]. Detecting the tissues of origin in both of these scenarios can assist in critical treatment decisions. We demonstrate that CancerNet is capable of robust and highly accurate metastatic tissue of origin classification and this performance is maintained in early cancer samples as well.

To assess CancerNet’s performance on metastatic cancer datasets, we first predicted tissue of origin for metastatic samples available in TCGA for BRCA, CESC, COAD, HNSC, PAAD, PCPG, PRAD, SARC, SKCM, THCA. The tissues of origin for all these metastatic cancers were predicted with an overall unweighted F-measure of 91%.

TCGA data were processed by the different labs and so it is possible that uninformative variance in noise could be introduced due to small but predictable variance in human error, reagent preparation or some other part of the sample processing pipeline. This is known as batch effect. Batch effect can provide a source of information about sample classes that, if learned, could make the model brittle in real world applications where the same effect is not present. We used several Gene Expression Omnibus (GEO) datasets to assess whether this was the case and further validate the model on non-TCGA derived data. These datasets also gave us the opportunity to test CancerNet’s performance on cancer stages that were not represented in TCGA, such as precancerous lesions. These, along with primary, metastatic, and recurrent samples, made possible the assessment of CancerNet’s performance across all stages of cancer for several tissue and cancer types.

The first dataset (GEO accession: GSE58999) contained paired metastatic and primary tumors in breast cancer patients. CancerNet achieved an unweighted F-measure of 99% on this dataset. The second dataset (GEO accession: GSE113019) contained triplets of liver samples from each patient, namely, non-tumorous, primary tumor and recurrent samples, respectively. CancerNet achieved an unweighted F-measure of 100% for all primary tumors, 100% for metastatic samples, and 85% for the normal samples. The third dataset (GEO accession: GSE38240) contained 4 normal samples and 8 PRAD metastatic samples; CancerNet attained unweighted F-measure of 88% and 93% for these classes, respectively.

The final dataset (GEO accession: GSE67116) consisted of 96 uterine samples that were stratified across cancer stages with precancerous endometrial hyperplasia, primary tumor and metastasis represented in addition to two cell lines. Samples were harvested from various tissue sites within the uterus. Because endometrial hyperplasia increases a patient’s risk of developing uterine cancer by 30% [39], we used these hyperplasia samples as putative cancer samples. We then labeled them as uterine cancer and checked the CancerNet output. CancerNet achieved an unweighted F-measure of 85% on this dataset. On all other sites, CancerNet achieved an unweighted F-measure of 92%. CancerNet produced an unweighted F-measure of 66% on hyperplasia samples, predicting them as uterine cancer in most cases. This indicates that CancerNet may be capable of cancer detection even when just precancerous lesions are present. However, cancer progression for the hyperplasia patients was not documented in the database and therefore, the fidelity of these predictions couldn’t be unambiguously established.

To further assess the predictive capability of CancerNet on precancerous samples, we used a dataset derived from 55 precancerous ductal carcinoma in situ samples (GEO accession: GSE66313). 40 of these samples later developed malignant forms of breast cancer. CancerNet identified the “future” cancer samples (40 of 55) with an unweighted F-measure of ~ 91% and “non-future” cancer samples (15 of 55) with an unweighted F-measure of ~ 66%, demonstrating that the model is capable of not only detecting cancer and its tissue of origin but has a reasonably high level of predictive capacity for pre-cancers as well without being explicitly trained to do so.

#### Age-related methylation drift

Age-related CpG methylation drift is the normal global hypomethylation associated with aging [40]. Some cancer etiologies may be associated with age-related methylation drift [40, 41]. CancerNet may be classifying based on background age-related methylation drift rather than methylation changes relevant to carcinogenesis. To verify that this was not the case, we used a dataset (GEO accession: GSE113904) with 232 age-stratified normal colon tissue samples. Samples were from individuals of age ranging from 29 to 81 years. CancerNet classified all of these samples correctly as normal regardless of age.

## Discussion

Here we developed and validated an end-to-end unified model for diagnosing multiple cancers. We achieved high performance, 99.6% overall accuracy (F-measure), through all cancer stages with robustness to possible confounding factors such as age. Previously published neural network classifiers performed approximately as well [18], ~ 96% overall accuracy (F-measure), however, they lack a latent space that can encode complex features present in the data. Incorporation of latent space architecture lends robustness to our model and allows for its possible extensions beyond the initial use demonstrated here. In addition, the model presented by Zeng and Xu [18] considers fewer features and predicts fewer cancer types. While we lack the tools to fully characterize the trained latent space, it can serve as a foundation for future research to develop explainability methods and potentially for the discovery of novel combinations of features that may be important for cancer etiology.

The overall performance of CancerNet on metastatic samples exceeds that of pathologists; the correct tissue was the first choice 49% of the time by pathologists [42], in contrast, correct tissue was the first choice 91% of the time by CancerNet when evaluated on TCGA metastatic cancer samples. CancerNet also substantially outperformed other models that perform cancer tissue of origin classification based on DNA methylation [15, 16] (for all 3 cancer types and a normal type investigated by CancerLocator [16] and 12 of 14 cancer types investigated by a model based on random forests [15], Additional file 1: Table S2). Strong results in both the metastatic and normal categories demonstrate that the model has learned reliable cancer signatures and is capable of tissue of origin detection in cancers that have undergone metastasis. Precancerous lesion classification does not fall neatly under the classification task for which CancerNet was trained. Due to the transitional nature of precancerous lesions, they could be classified as normal tissue, or predicted as cancerous, which they may become. The performance of CancerNet on precancerous samples is promising and is likely the result of the latent space prior for the classification task. If more precancerous samples for which the progression is known are made available, it may then be possible to add a predictive task to the model and train the model for that specific task. Together, the performance across the cancer spectrum is consistent and demonstrates the robustness of CancerNet.

Where efforts have been made to focus on cancer tissue-of-origin detection, some studies, surprisingly, have done so without determining whether a sample is cancerous or not. Without non-cancerous classes incorporated within a model framework, the model may actually learn tissue specific signatures due to the retention of cell specific methylation signatures even in carcinogenesis. This approach may thus lead to a model learning normal tissue signatures rather than cancer signatures. Therefore, it is pertinent to include normal samples to allow the model to learn to discriminate between normal tissue specific signatures and tumor tissue specific signatures. We, therefore, included normal samples in CancerNet training and classification and ensured that CancerNet’s performance is not an artifact of tissue specific signatures. Assessment on different datasets demonstrates that CancerNet is able to robustly diagnose cancer and detect the cancer tissue of origin as well.

Robust tissue level classification is a huge step forward for early cancer detection. Indeed, many cancers have no early diagnostic whatsoever. The clinical use of such a model will benefit from inclusion of information about tumor evolution and tumor subtypes. Such information would aid in treatment decisions and prognosis determination. It is our belief that clinical diagnostic is not the only significant use of such a model. Research in cancer biology may be aided by investigating the learned features in the model’s latent space. Such features may illuminate complex interactions between multiple mutations and methylation dysregulation in a given cellular context. This could provide valuable information about new drug targets. The value of this information coming from a unified model cannot be understated as it provides the opportunity to find potential targets present in multiple cancers and subdivide tumors in feature space rather than in anatomical space allowing discernment of yet unknown aspects of the tumor microenvironment and its effects on oncogenic pathways by way of epigenetics.

Detecting cancer in asymptomatic patients or screening population for cancers requires minimally invasive procedures. Current methods of screening body fluids for biomarkers have been proposed for use with circulating cell-free DNA, cfDNA [43–46]. Several studies have shown that methylation persists on the fragments of circulating tumor DNA (ctDNA) and is stable enough to provide cancer diagnosis and tissue of origin classification [47–51]. Several key steps must be taken to adapt CancerNet for use with ctDNA. Primarily the number of CpG islands present in a sample at different stages must be assessed. If the model relies on far more features than can feasibly be found in a typical sample, then the model must be adapted to that reality. Additionally, circulating cfDNA may come from multiple sources. Presumably the majority of DNA fragments could come from cells such as macrophages or other normal tissues with good access to the blood that are turned over at a fair rate. Filtering samples to identify the ctDNA fragments of interest is a necessary preprocessing step. We expect technological advances in cfDNA processing will make possible non-invasive, robust early diagnosis of cancers and tissue of origin determination using emerging tools from the field of artificial intelligence such as CancerNet.

## Supplementary Information

**Additional file 1.** Supplementary figures.

**Additional file 2.** CpG feature assignement and sample assignment to test, train and validation sets.

## Data Availability

The software and associated datasets are available at the Open Science Framework (OSF) site: https://osf.io/j6gcv/?view_only=d18c6831b9e24002937ab1028fc0d418. All other datasets are provided with the article and supplementary materials. We also declare that no biomolecular data (e.g. proteomics data and protein sequences, DNA and RNA sequences, genetic polymorphisms, linked genotype and phenotype data, macromolecular structure, gene expression data, crystallographic data for small molecules) were generated from this work. The GEO datasets used in this study were obtained from NCBI GEO repository (https://www.ncbi.nlm.nih.gov/geo/), with accession numbers GSE113019, GSE113019, GSE38240, GSE58999, GSE66313, GSE67116.

## Abbreviations

ACC: Adrenocortical carcinoma
BLCA: Bladder urothelial carcinoma
BRCA: Breast invasive carcinoma
CESC: Cervical squamous cell carcinoma and endocervical adenocarcinoma
CHOL: Cholangiocarcinoma
CIMP: CpG island methylator phenotype
COAD: Colon adenocarcinoma
DLBC: Lymphoid neoplasm diffuse large B-cell lymphoma
ESCA: Esophageal carcinoma
GBM: Glioblastoma multiforme
HNSC: Head and neck squamous cell carcinoma
HPV: Human papillomavirus
KICH: Kidney chromophobe
KIRC: Kidney renal clear cell carcinoma
KIRP: Kidney renal papillary cell carcinoma
LAML: Acute myeloid leukemia
LGG: Brain lower grade glioma
LIHC: Liver hepatocellular carcinoma
LUAD: Lung adenocarcinoma
LUSC: Lung squamous cell carcinoma
MESO: Mesothelioma
NORM: Normal (non-cancer)
OV: Ovarian serous cystadenocarcinoma
PAAD: Pancreatic adenocarcinoma
PCPG: Pheochromocytoma and paraganglioma
PRAD: Prostate adenocarcinoma
READ: Rectum adenocarcinoma
SARC: Sarcoma
SKCM: Skin cutaneous melanoma
STAD: Stomach adenocarcinoma
TGCT: Testicular germ cell tumors
THCA: Thyroid carcinoma
THYM: Thymoma
UCEC: Uterine corpus endometrial carcinoma
UCS: Uterine carcinosarcoma
UVM: Uveal melanoma
