# Supplementary material for: CancerNet: a unified deep learning network for pan-cancer diagnostics
Source: BMC Bioinformatics. 2022 Jun 13;23:229. doi: 10.1186/s12859-022-04783-y (PMC9195411; doi:10.1186/s12859-022-04783-y)
Supplement: Supplementary file 1 — Additional file 1. Supplementary figures. [file 12859_2022_4783_MOESM1_ESM.docx]

**Supplementary Materials**

Supplementary Table 1: GEO dataset information

| GEO accession number | Total number of samples | Primary site and comment |
| --- | --- | --- |
| GSE113019 | 55 | Liver- primary, tumor adjacent and metastasis |
| GSE113904 | 232 | COAD- age related drift study. no cancer data, all normal |
| GSE38240 | 12 | Prostate- metastatic |
| GSE58999 | 88 | Metastatic breast |
| GSE66313 | 55 | Ductal carcinoma breast |
| GSE67116 | 96 | Uterine metastasis |

Supplementary Table 2: Performance of CancerNet, CancerLocator, and a model based on random forest (RF model) in classifying 14 cancers encompassing hard tumors of the abdominal and thoracic cavities that were investigated in CancerLocator or RF model or both these studies. Performance was assessed using the overall accuracy metric F-measure. Highest F-measure value among the three methods is shown in boldface for each cancer type, as well as for the normal class. “x” denotes no value for a cancer (or normal) that was not included in the classification by a method.

| Type | RF model | CancerLocator | CancerNet |
| --- | --- | --- | --- |
| Urothelial Bladder Carcinoma | 0.673 | x | **0.999** |
| Breast Invasive Carcinoma | 0.979 | 0.476 | **0.998** |
| Colon Adenocarcinoma | **1.000** | x | 0.989 |
| Esophagial Carcinoma | 0.336 | x | **0.989** |
| Head and Neck Squamous Cell Carcinoma | 0.720 | x | **0.992** |
| Kidney Renal Clear Cell | 0.739 | x | **0.998** |
| Kidney Renal Papillary Cell | 0.695 | x | **0.998** |
| Liver Hepatocellular carcinoma | 0.965 | 0.847 | **0.998** |
| Lung Squamous Cell Carcinoma | 0.406 | x | **0.995** |
| Lung Adenocarcinoma | 0.773 | 0.612 | **0.997** |
| Pancreatic Adenocarcinoma | 0.712 | x | **0.997** |
| Prostate Adenocarcinoma | **1.000** | x | 0.997 |
| Thyroid carcinoma | 0.992 | x | **0.999** |
| Uterine Corpus Endometrial Carcinoma | 0.918 | x | **0.994** |
| Normal | x | 0.795 | **0.988** |

Supplementary Figures 1 – 38: Misclassification rates for cancers that were misclassified to one or more other cancer(s).


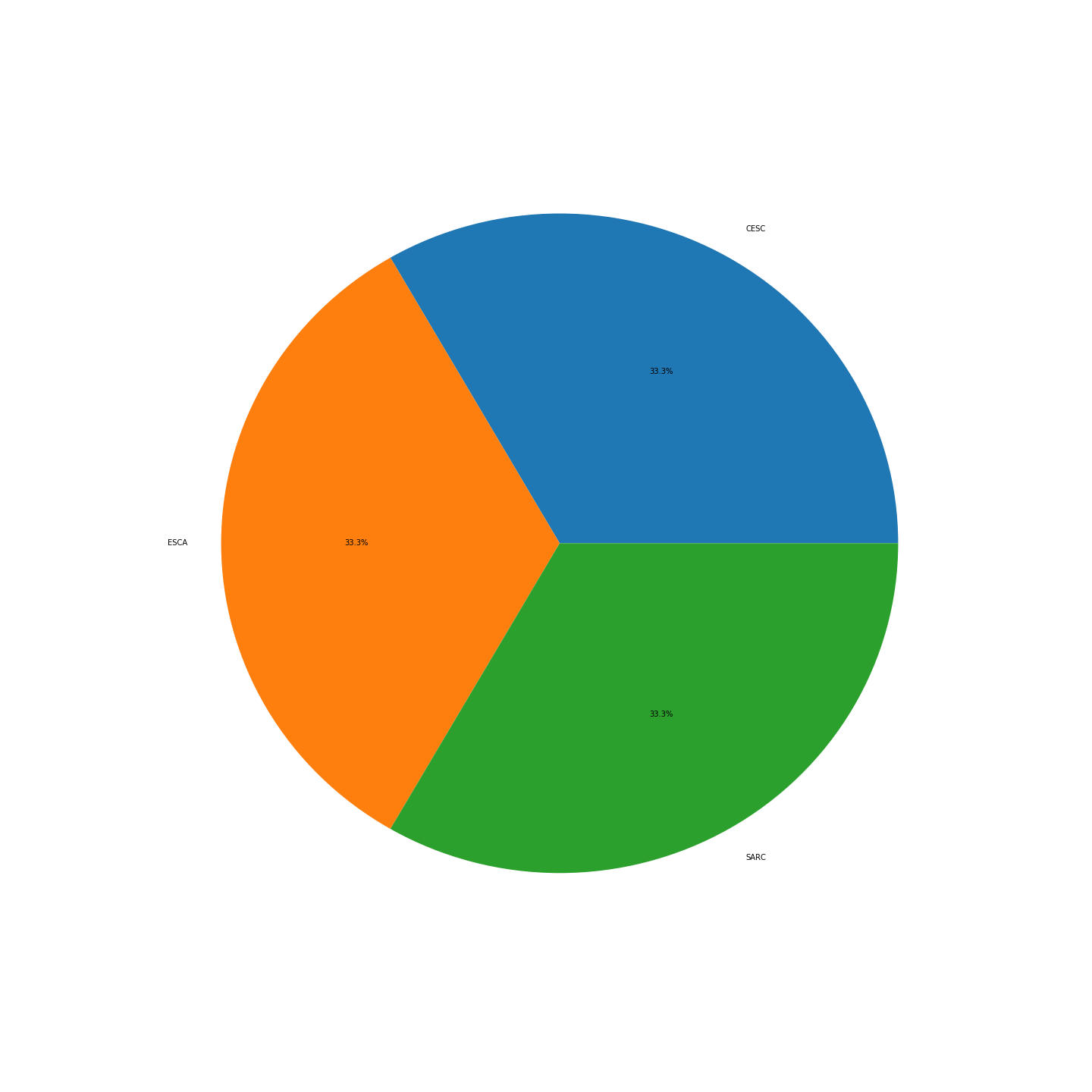


Supplementary Figure 1: BLCA


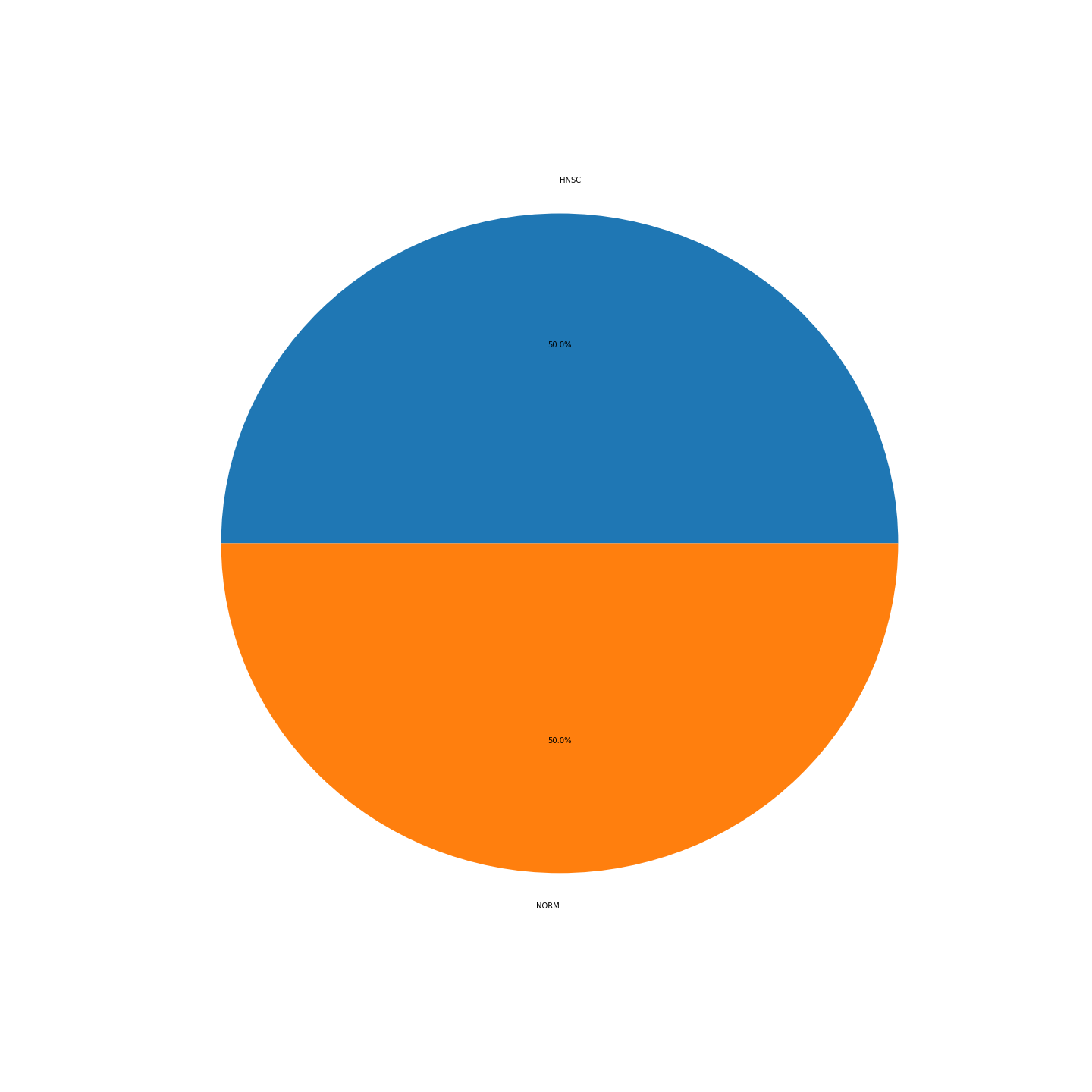


Supplementary Figure 2: BRCA


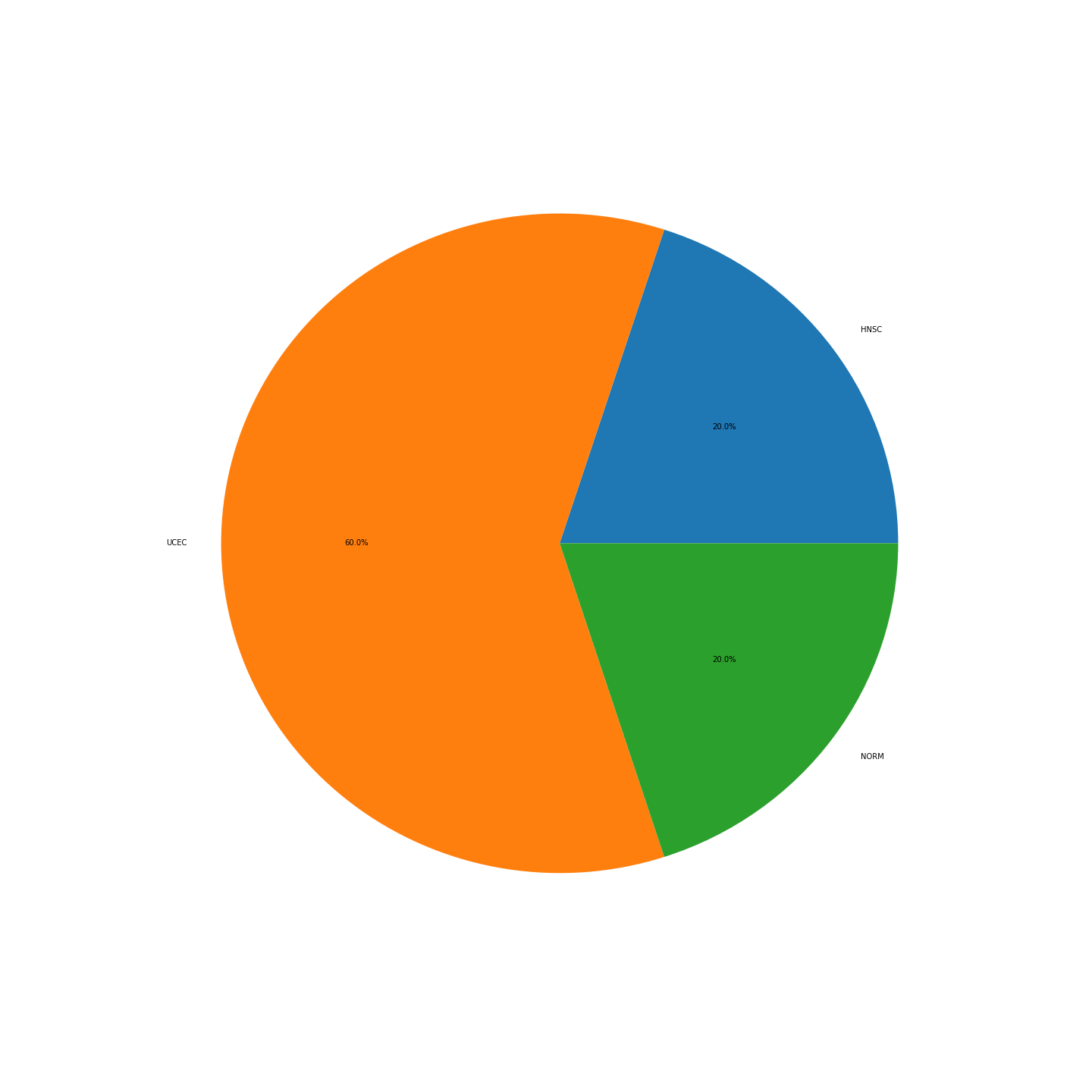


Supplementary Figure 3:CESC


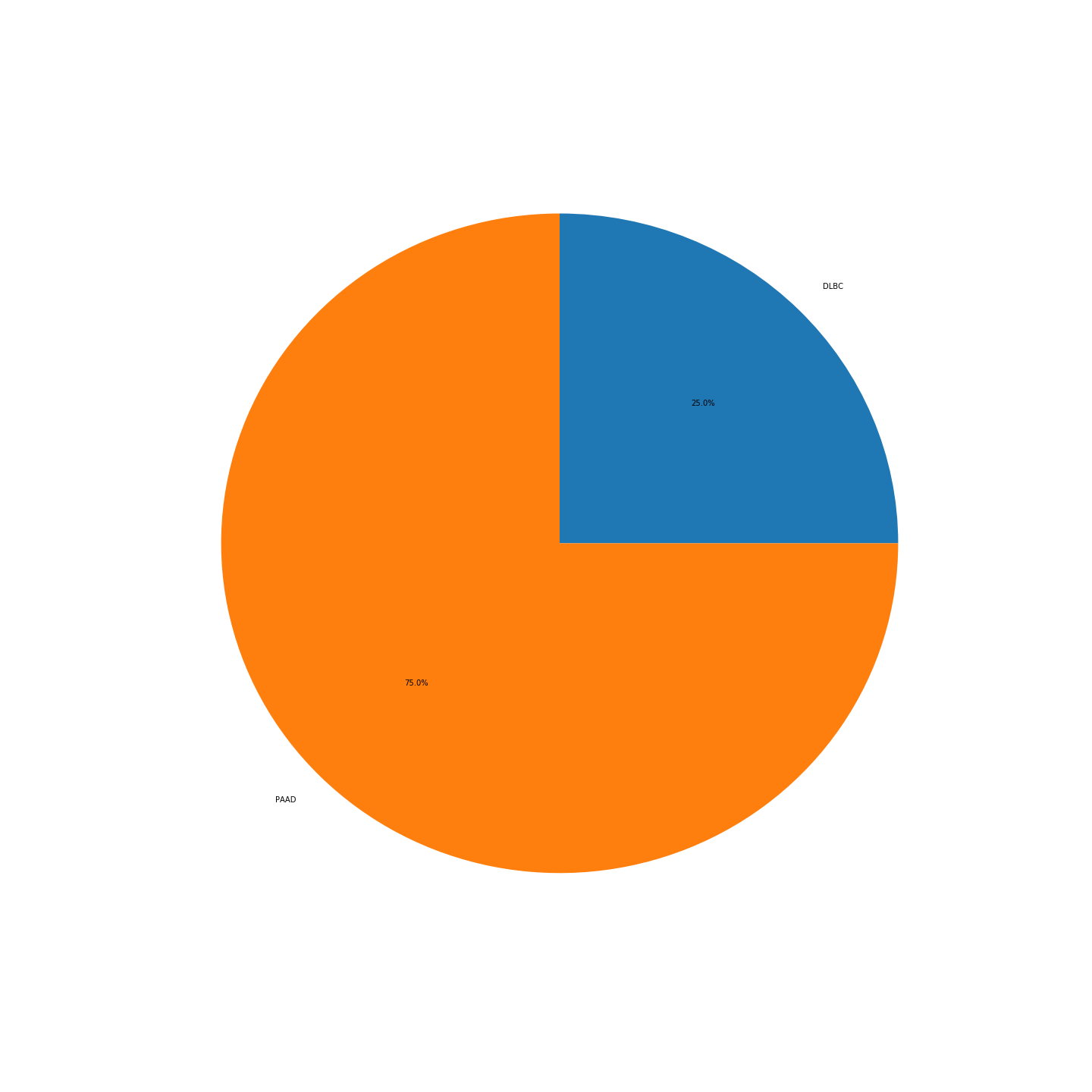


Supplementary Figure 4: CHOL


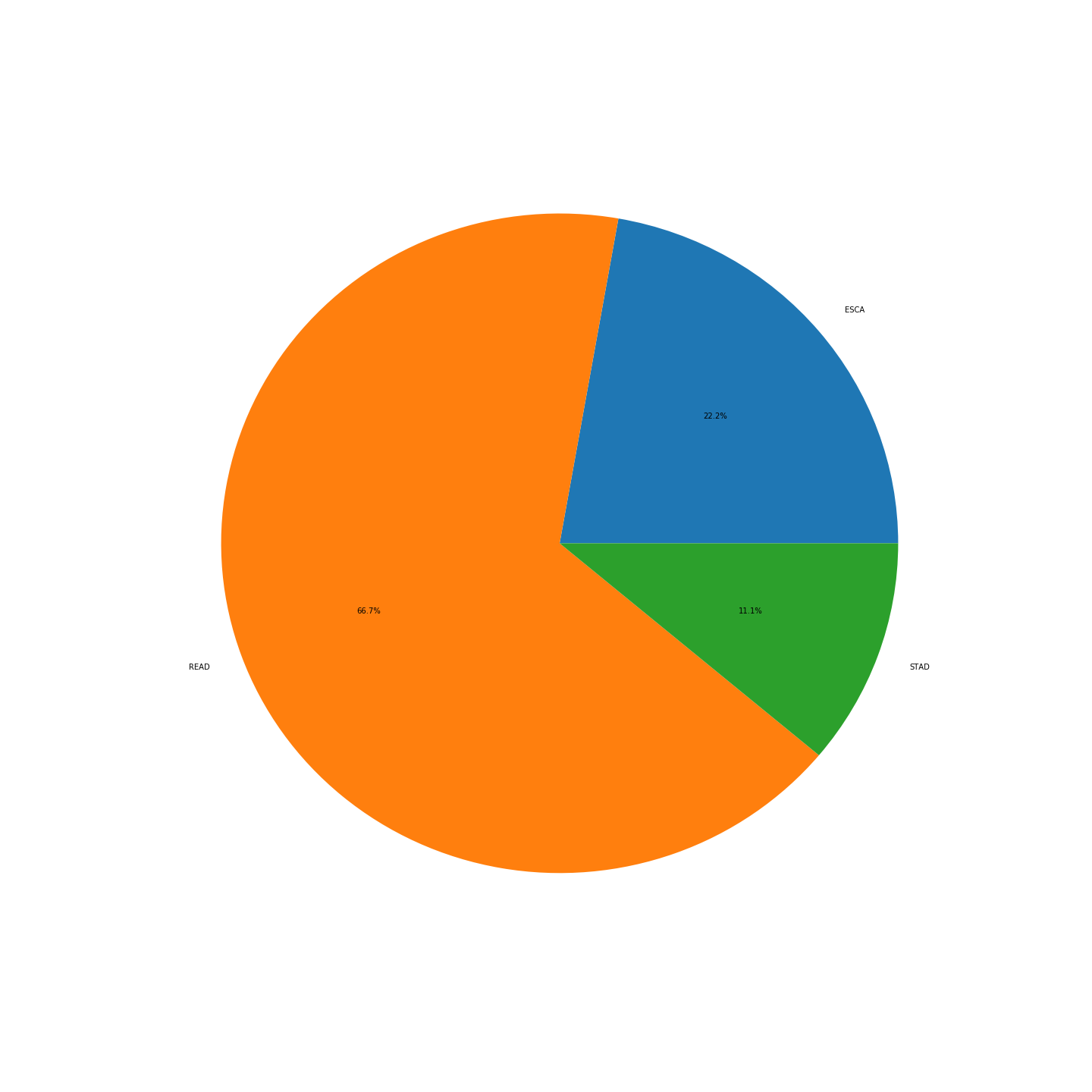


Supplementary Figure 5: COAD


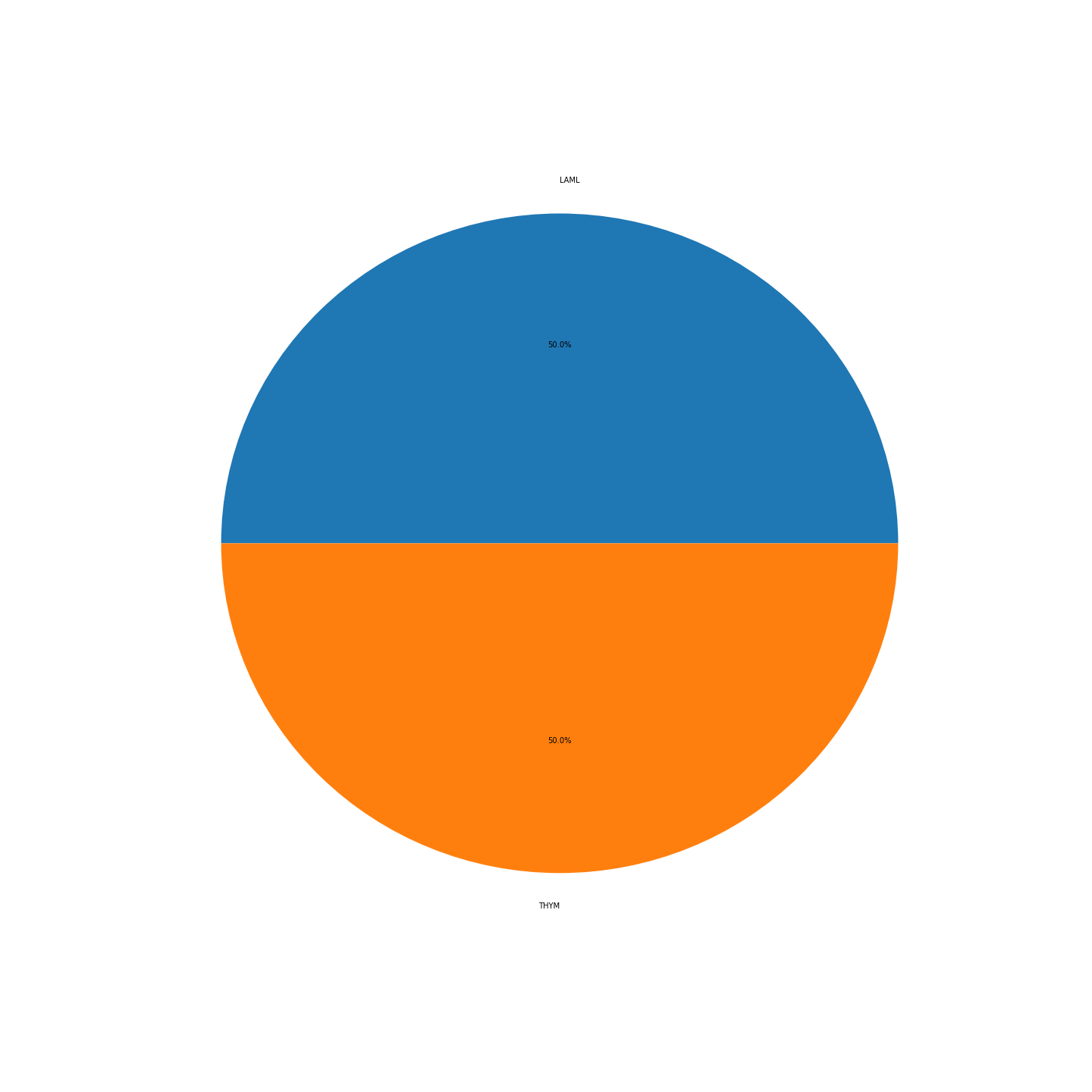


Supplementary Figure 6: DLBC


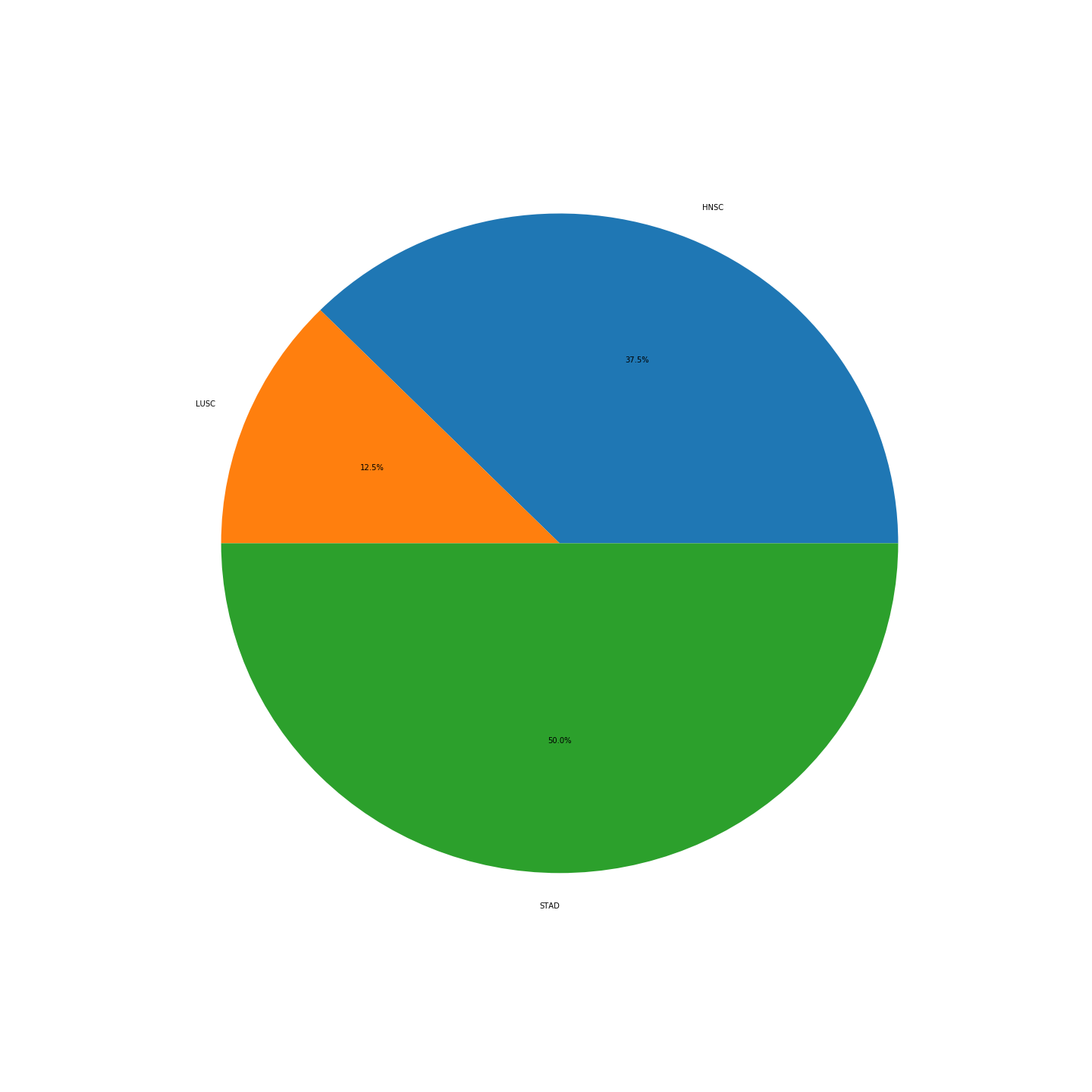


Supplementary Figure 7: ESCA


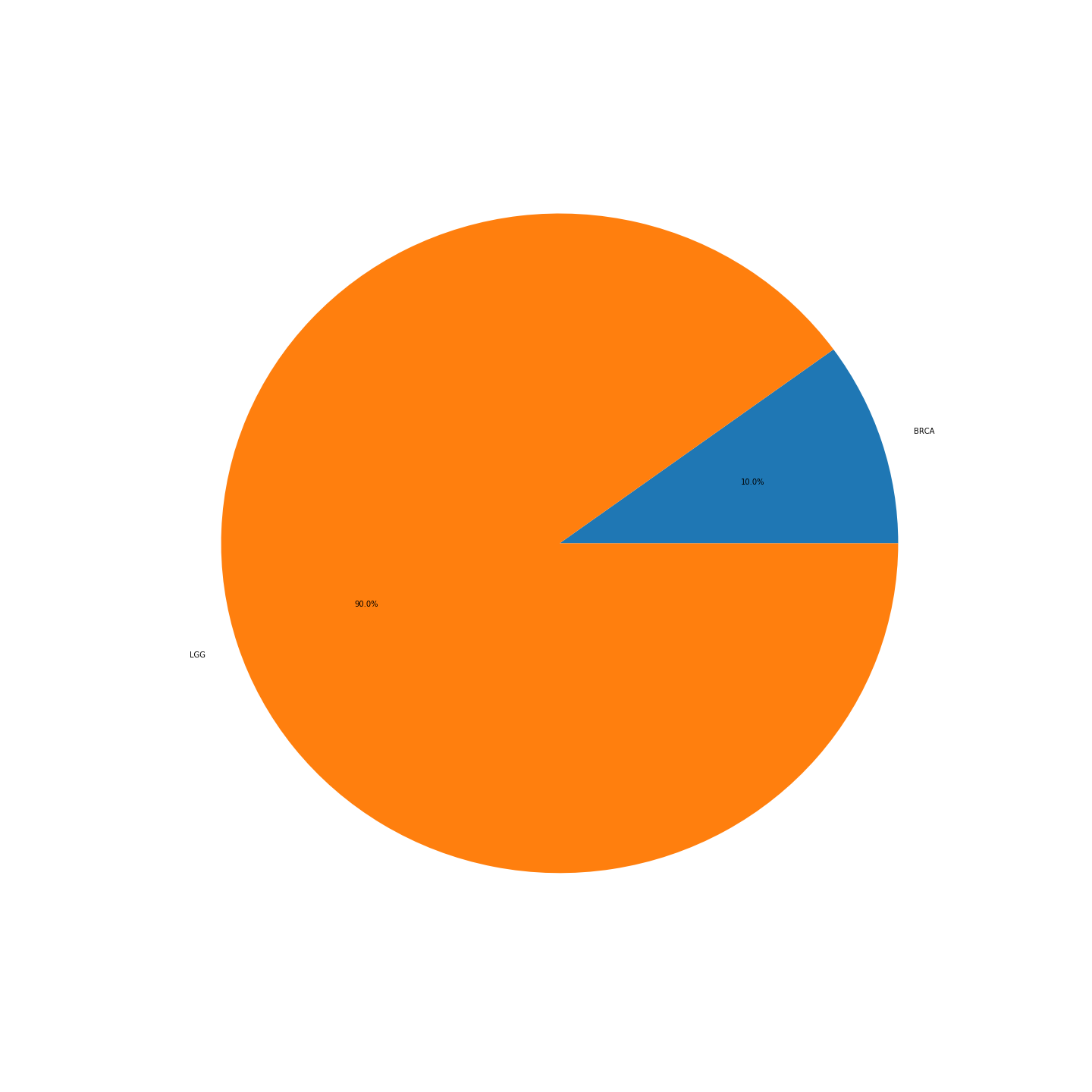


Supplementary Figure 8: GBM


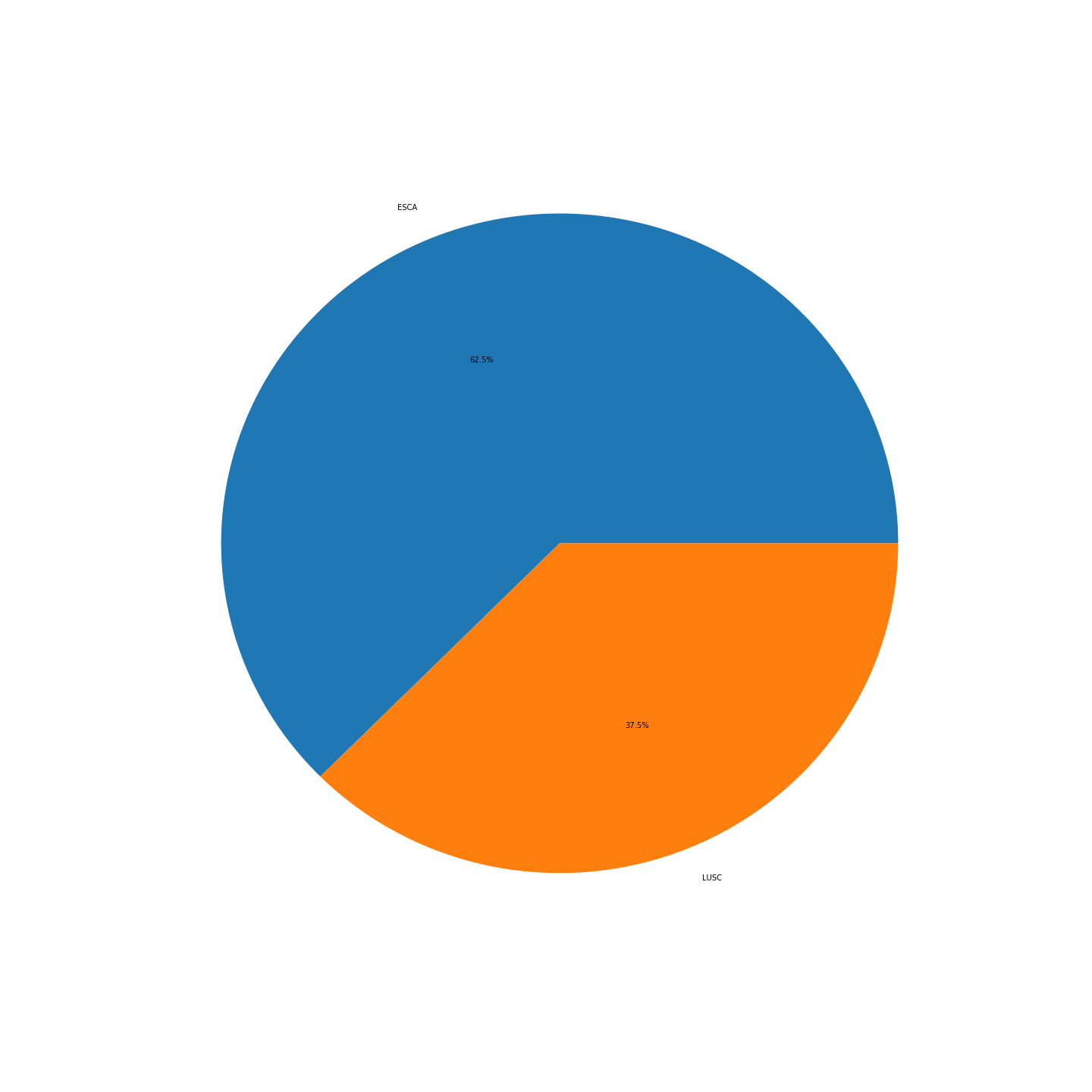


Supplementary Figure 9: HNSC


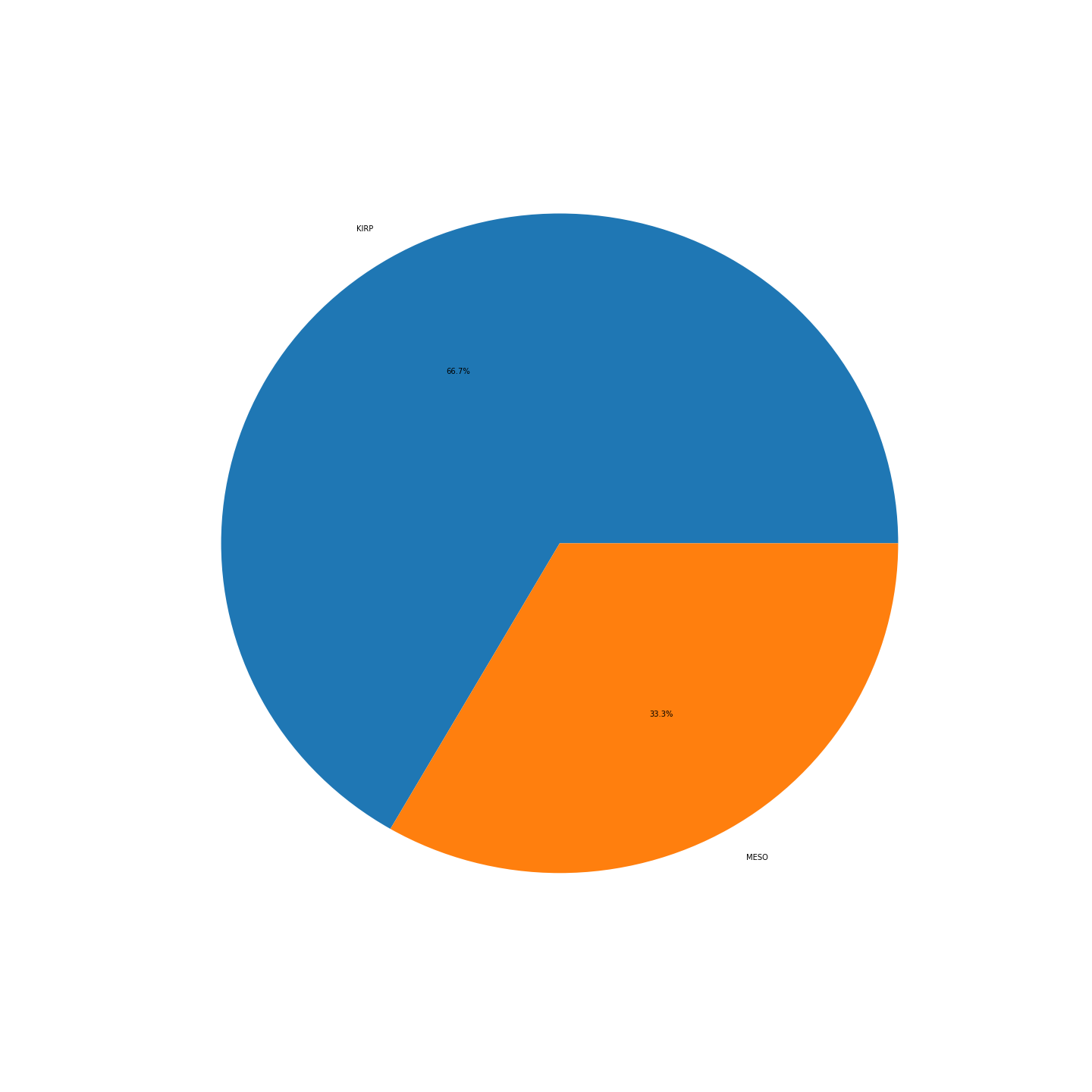


Supplementary Figure 10: KIRC


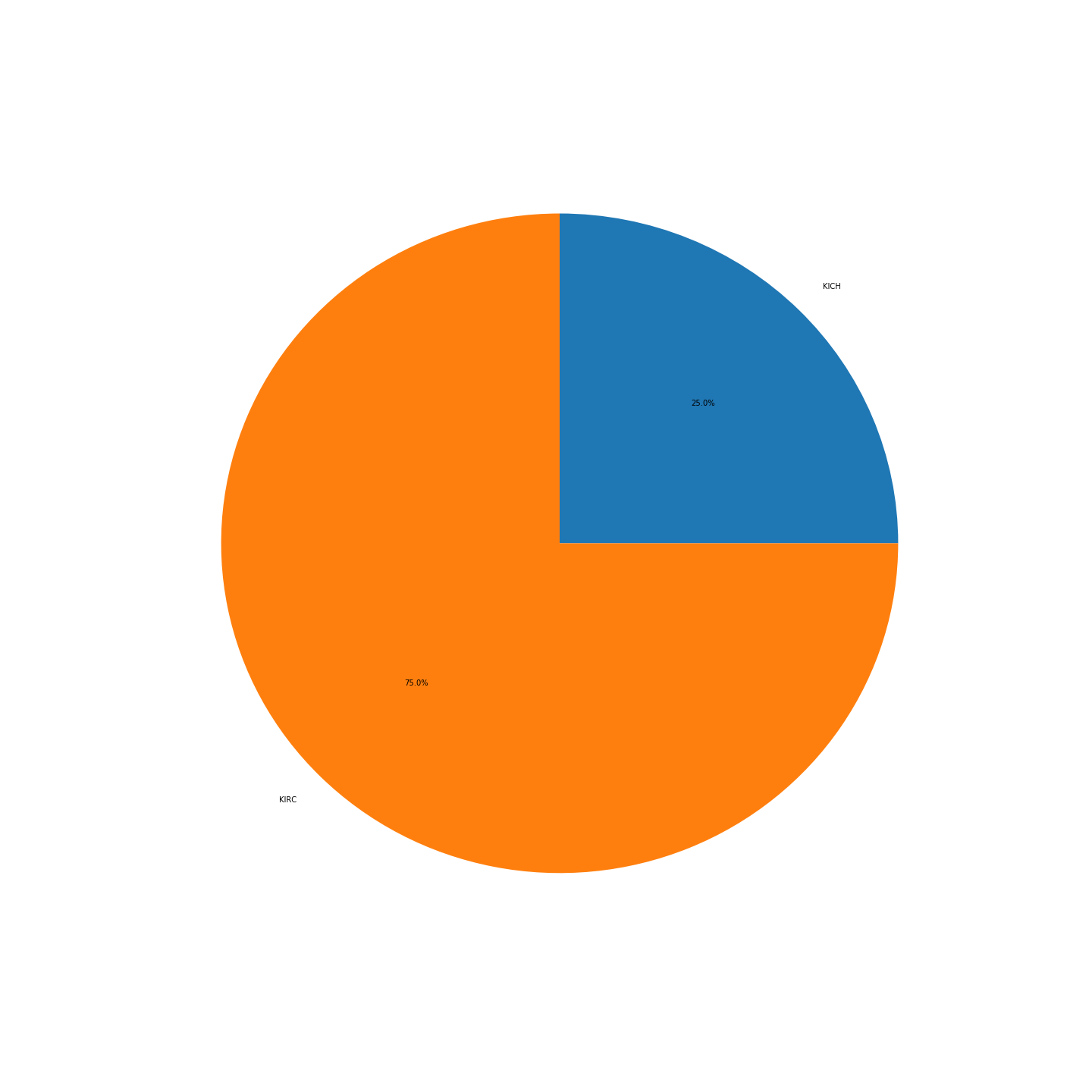


Supplementary Figure 11: KIRP


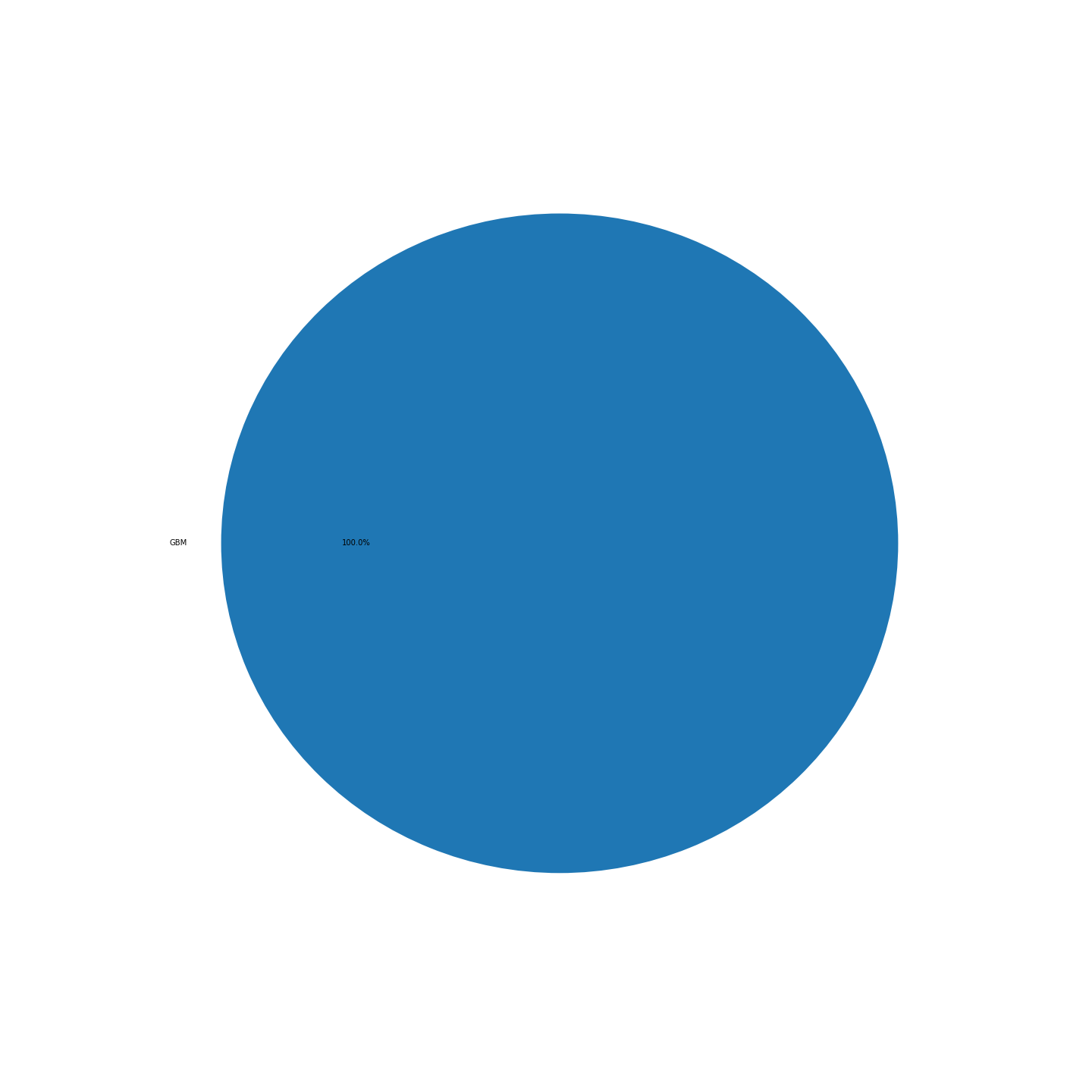


Supplementary Figure 12:LGG


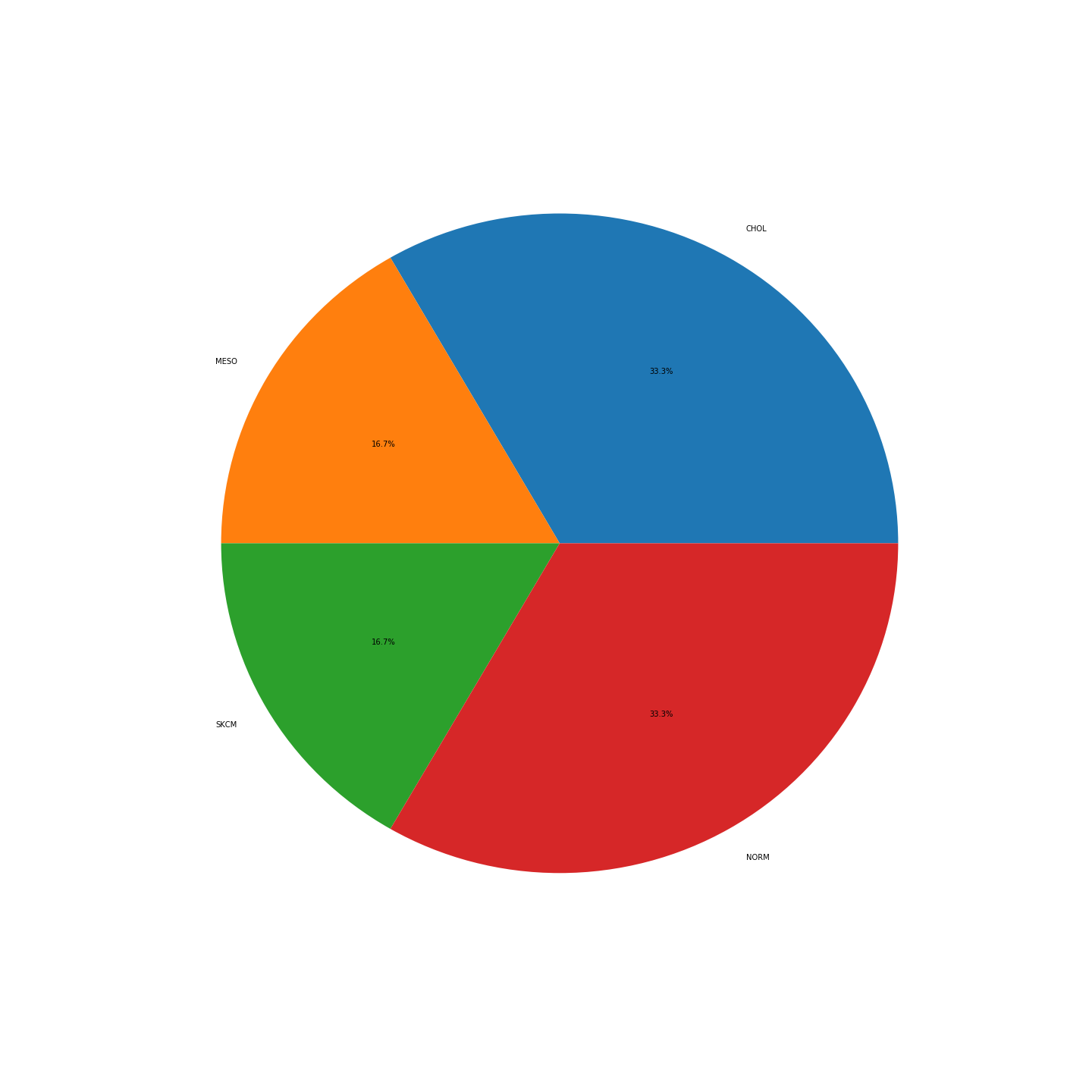


Supplementary Figure 13: LIHC


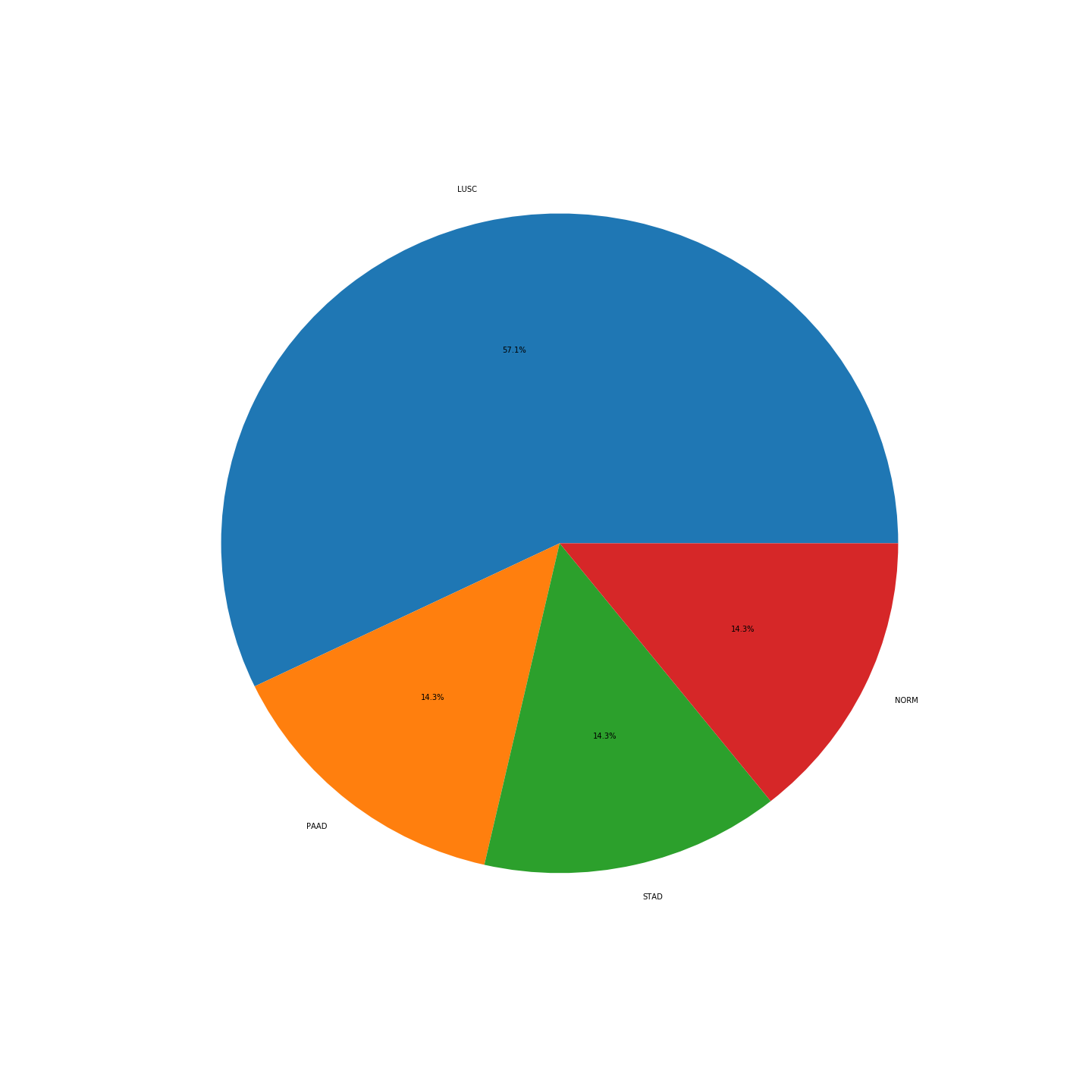


Supplementary Figure 14:LUAD


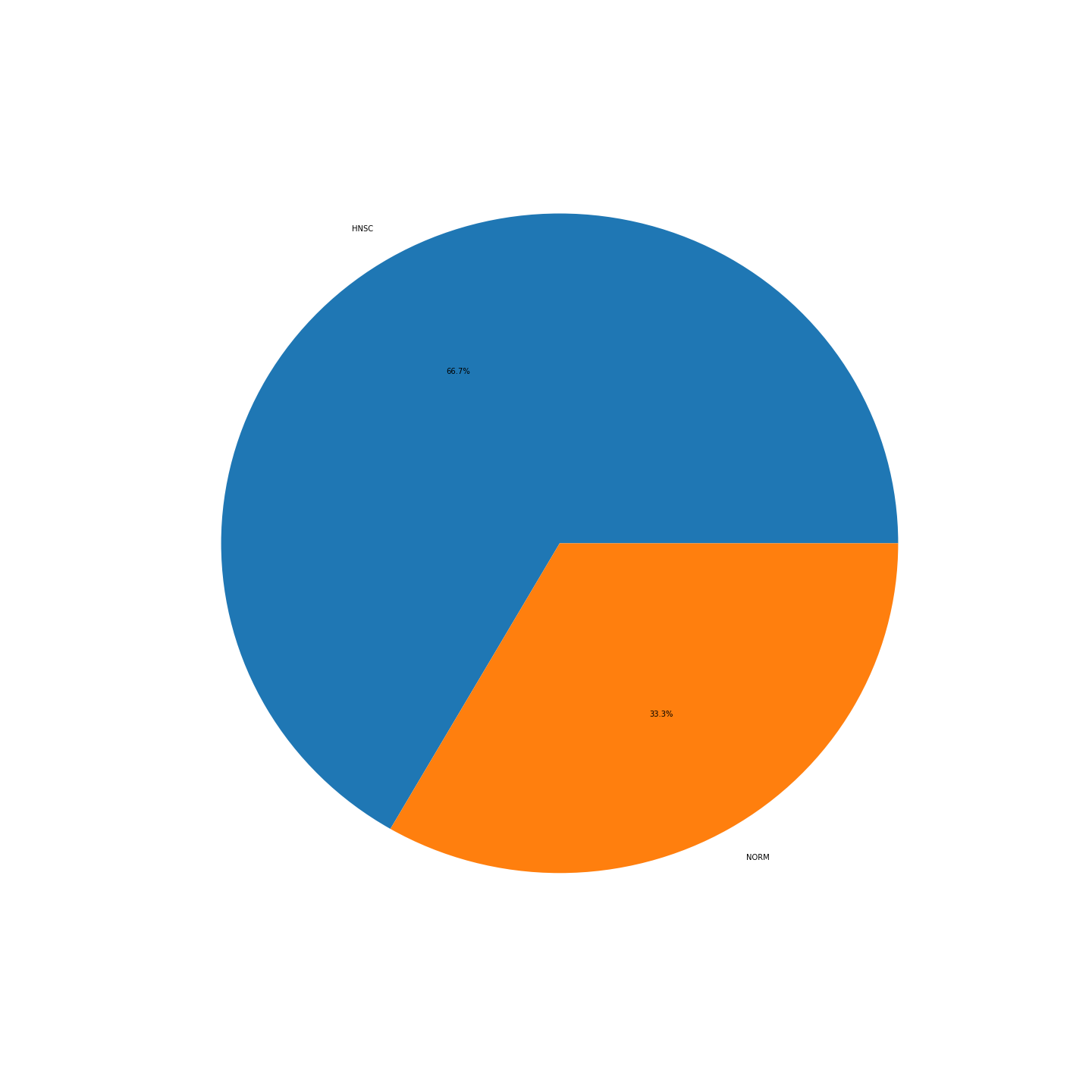


Supplementary Figure 15: LUSC


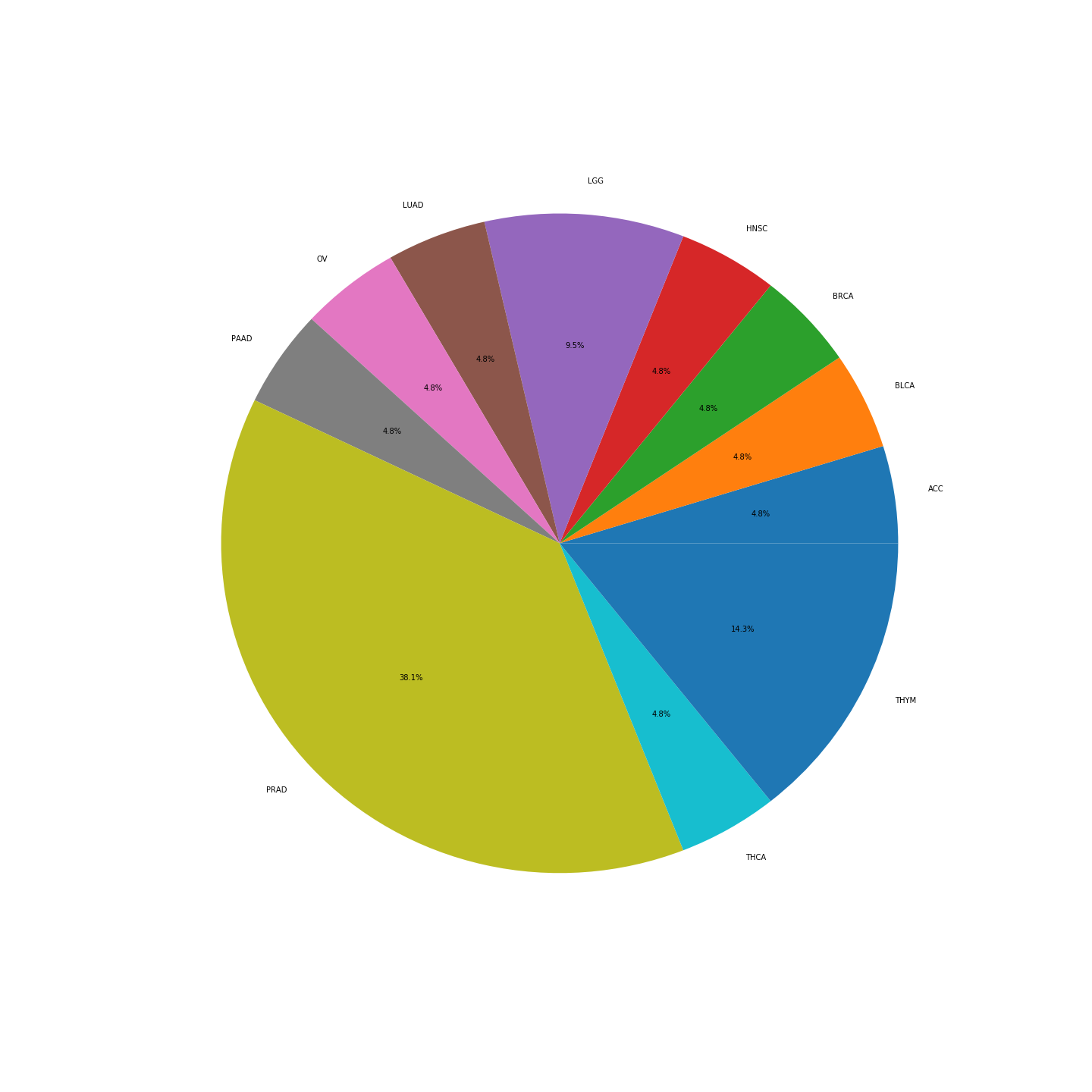


Supplementary Figure 16: NORM


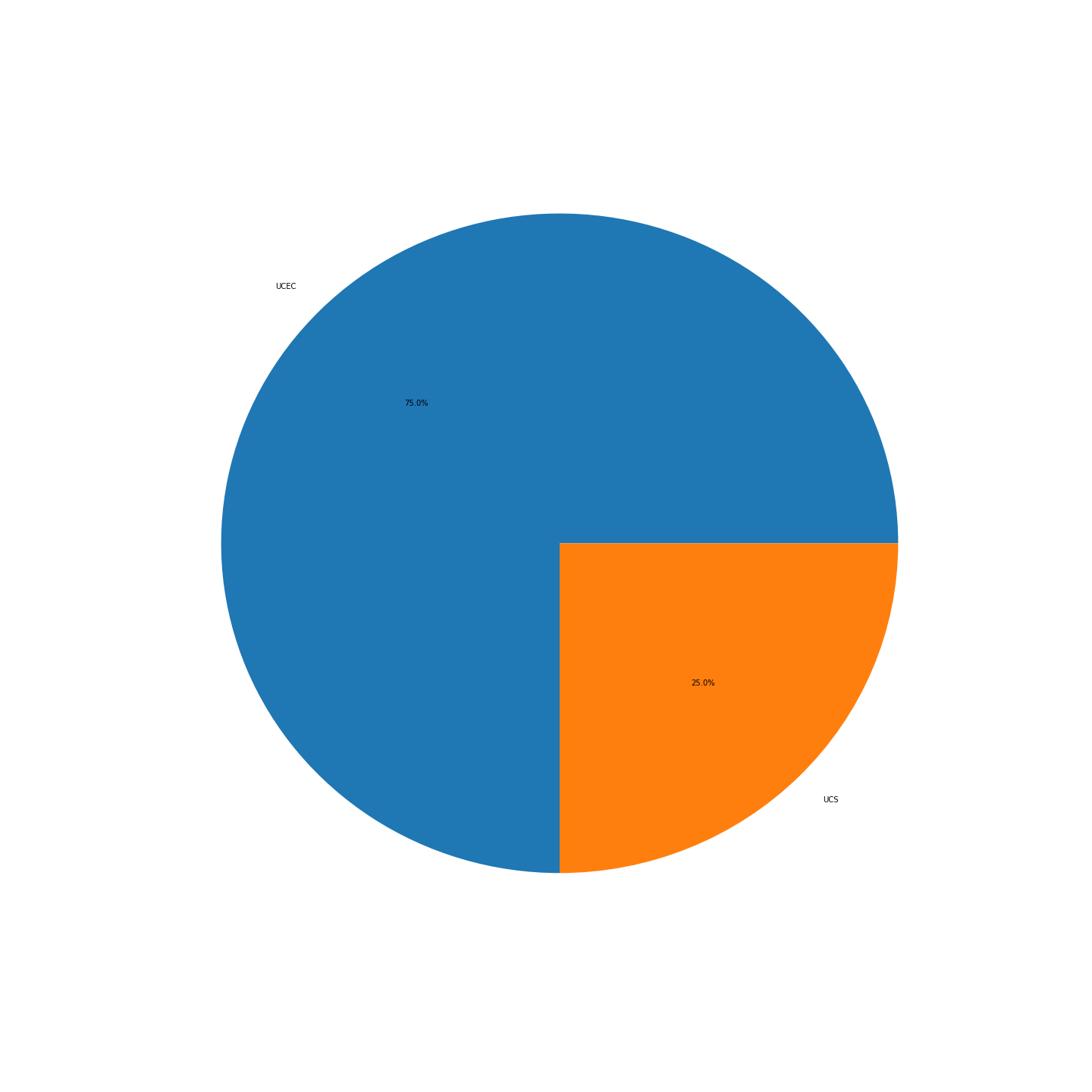


Supplementary Figure 17: OV


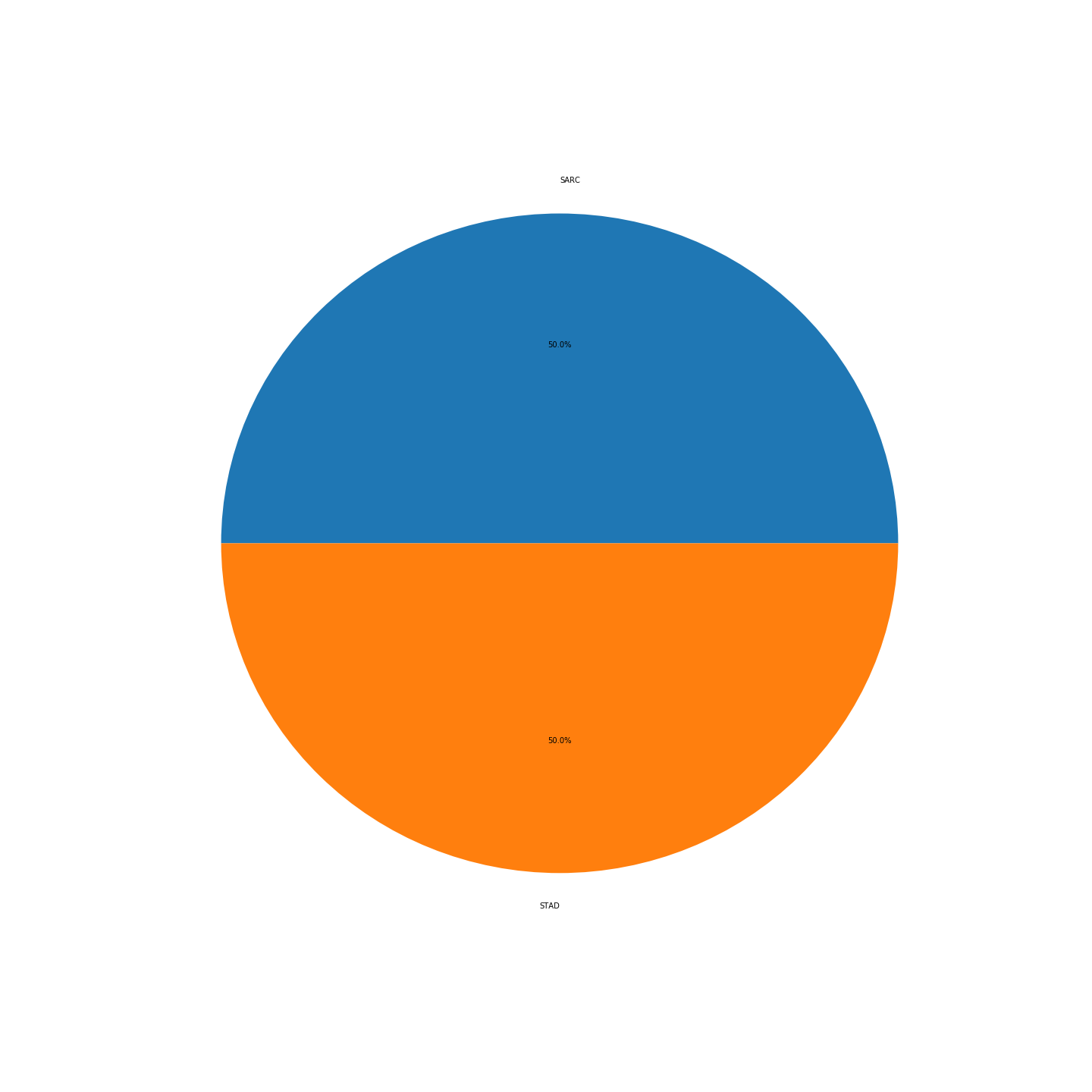


Supplementary Figure 18: PAAD


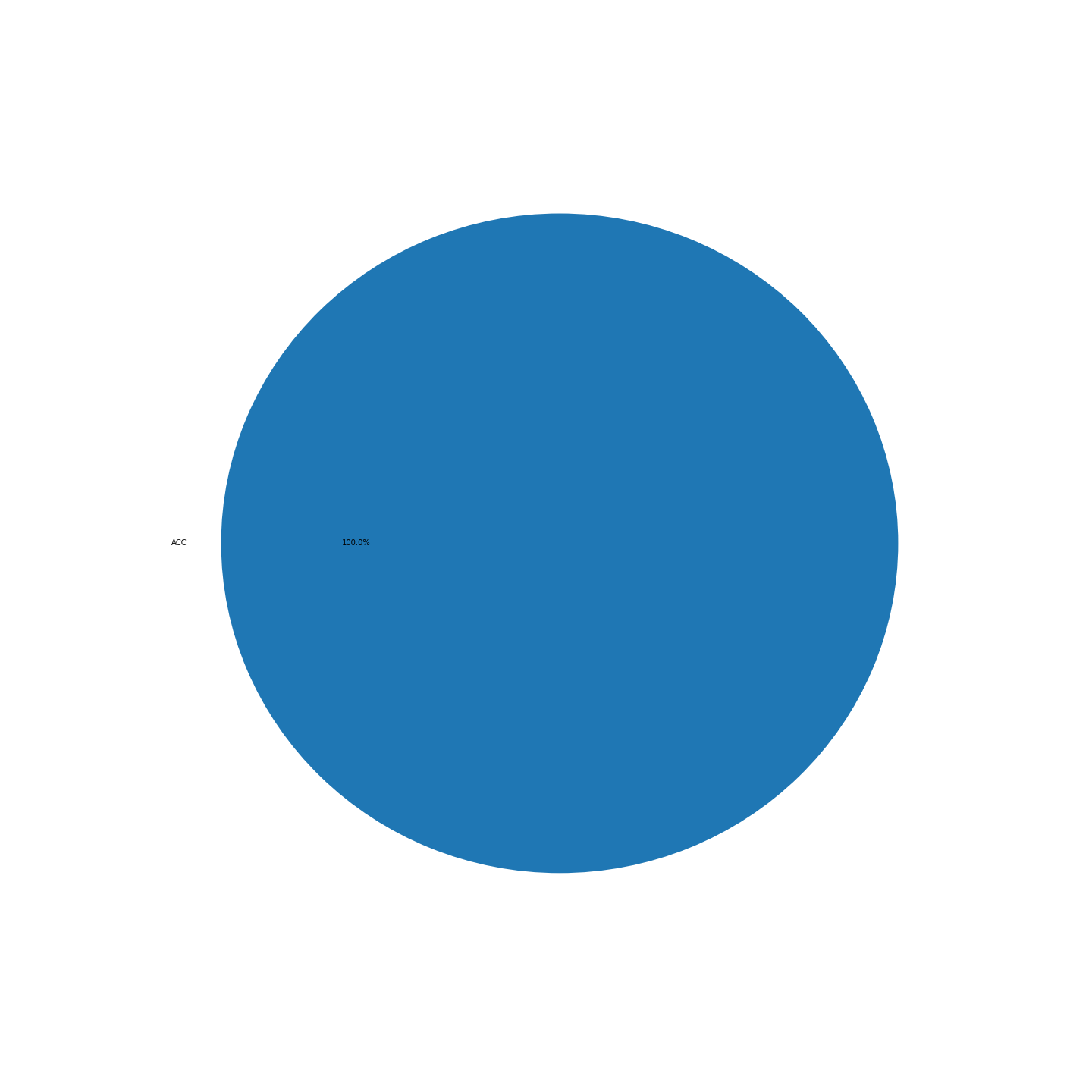


Supplementary Figure 19:PCPG


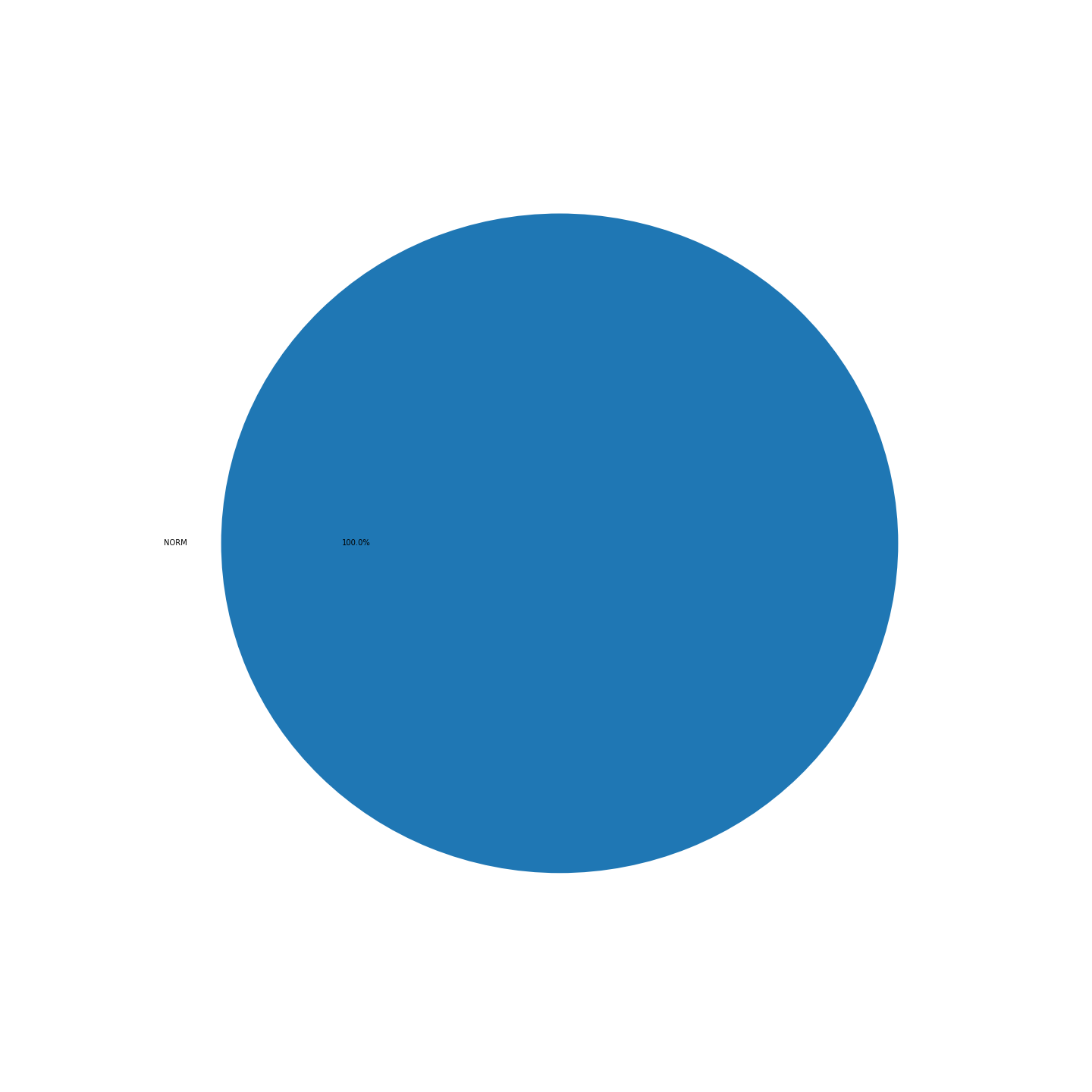


Supplementary Figure 20:PRAD


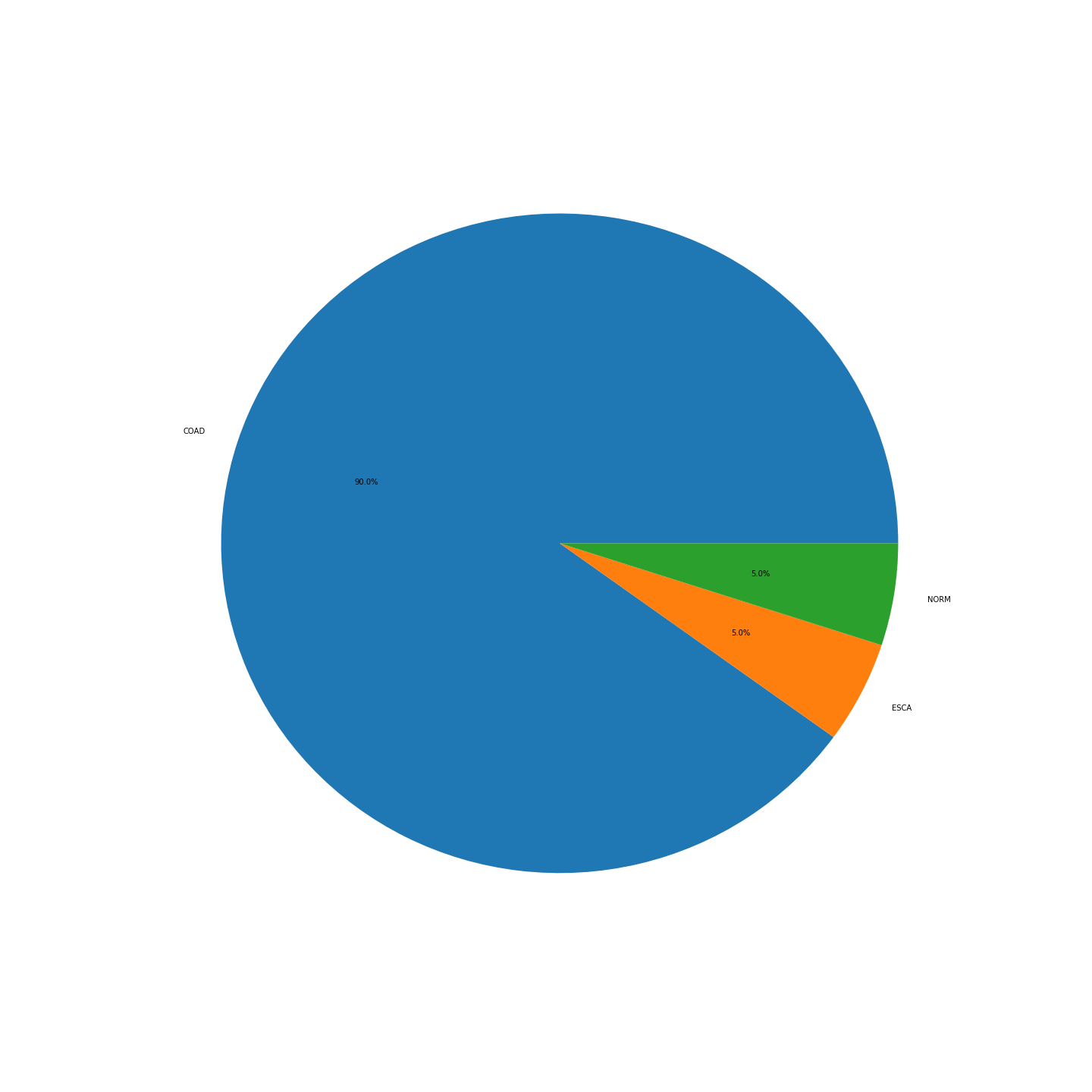


Supplementary Figure 21: READ


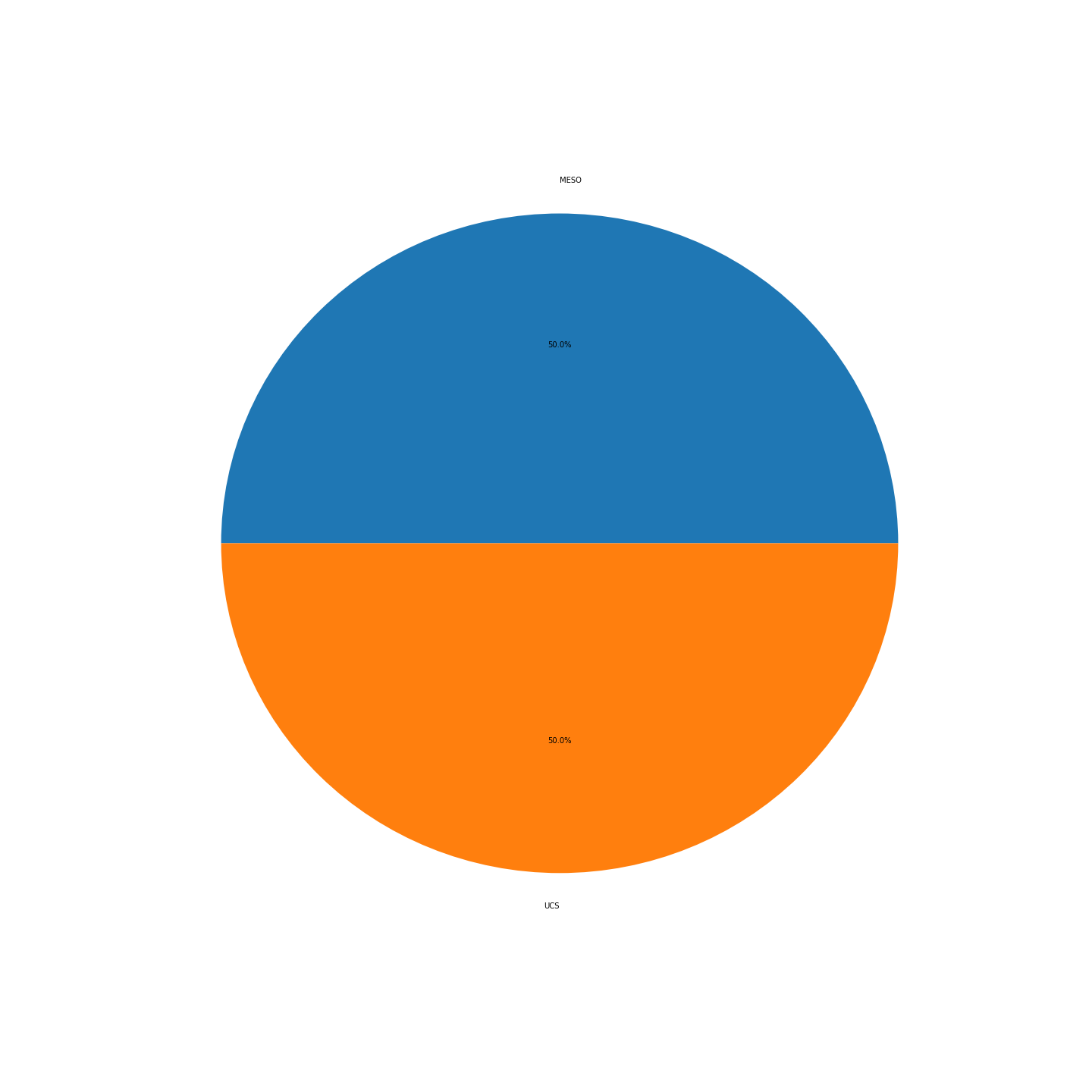


Supplementary Figure 22:SARC


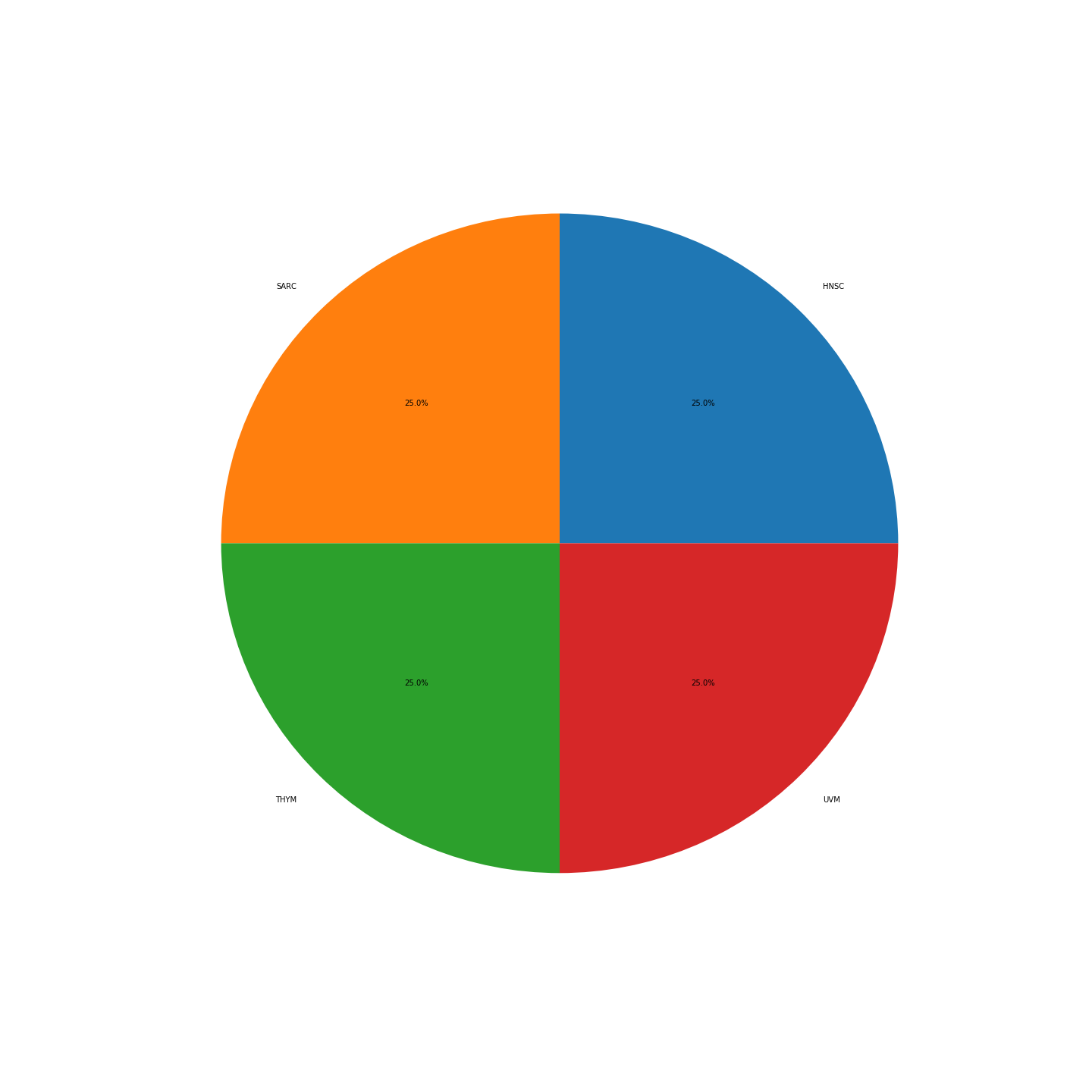


Supplementary Figure 23:SKCM


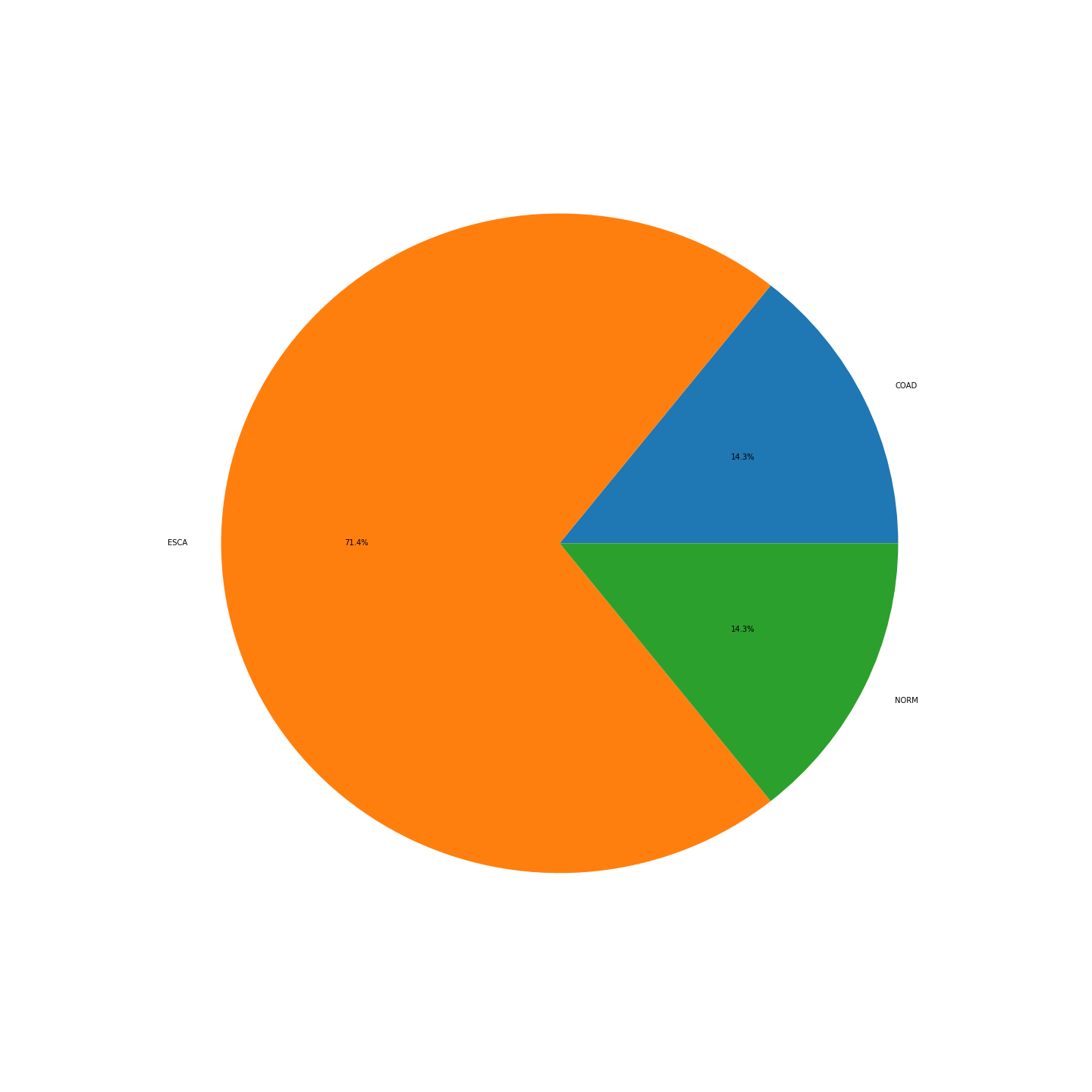


Supplementary Figure 24:STAD


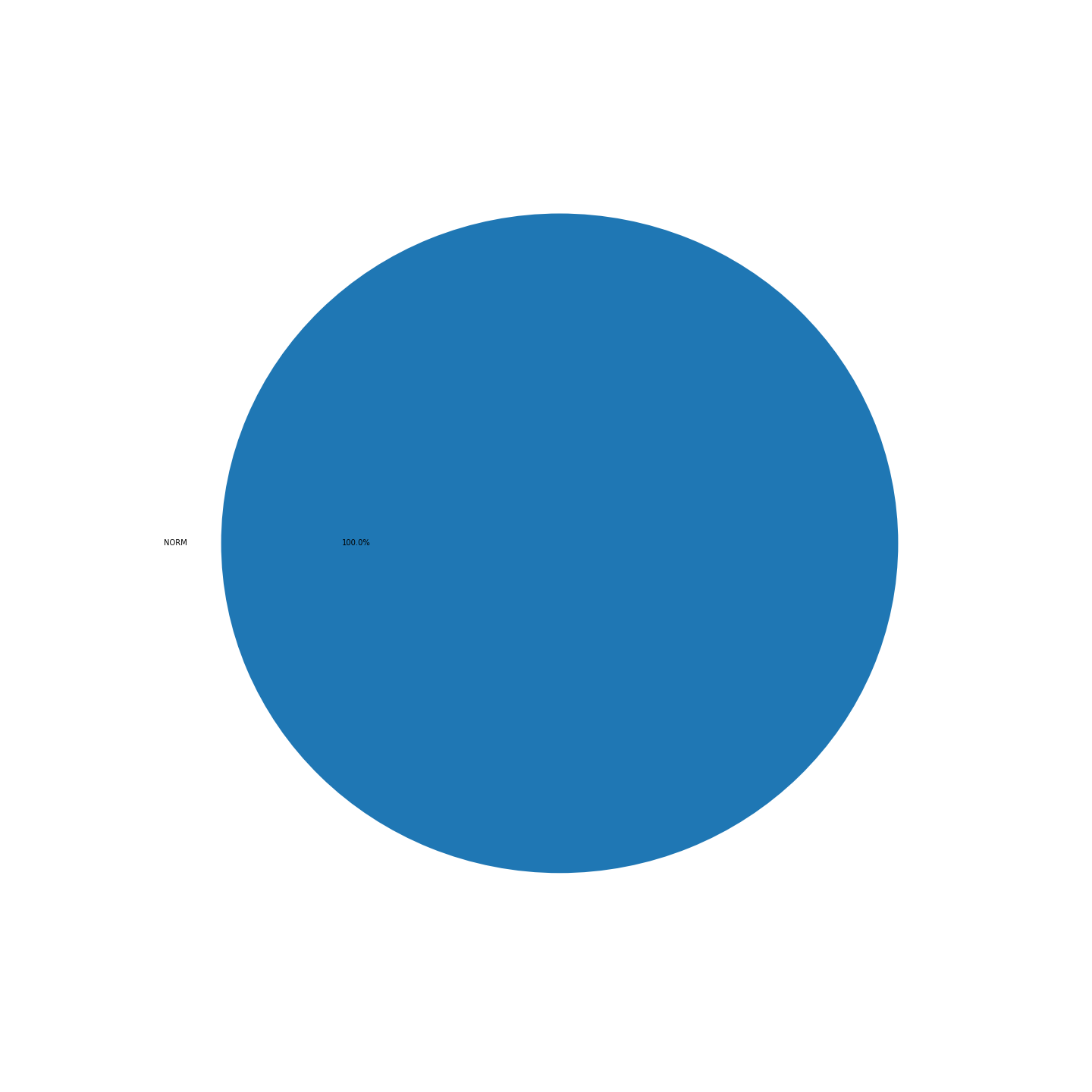


Supplementary Figure 25:THCA


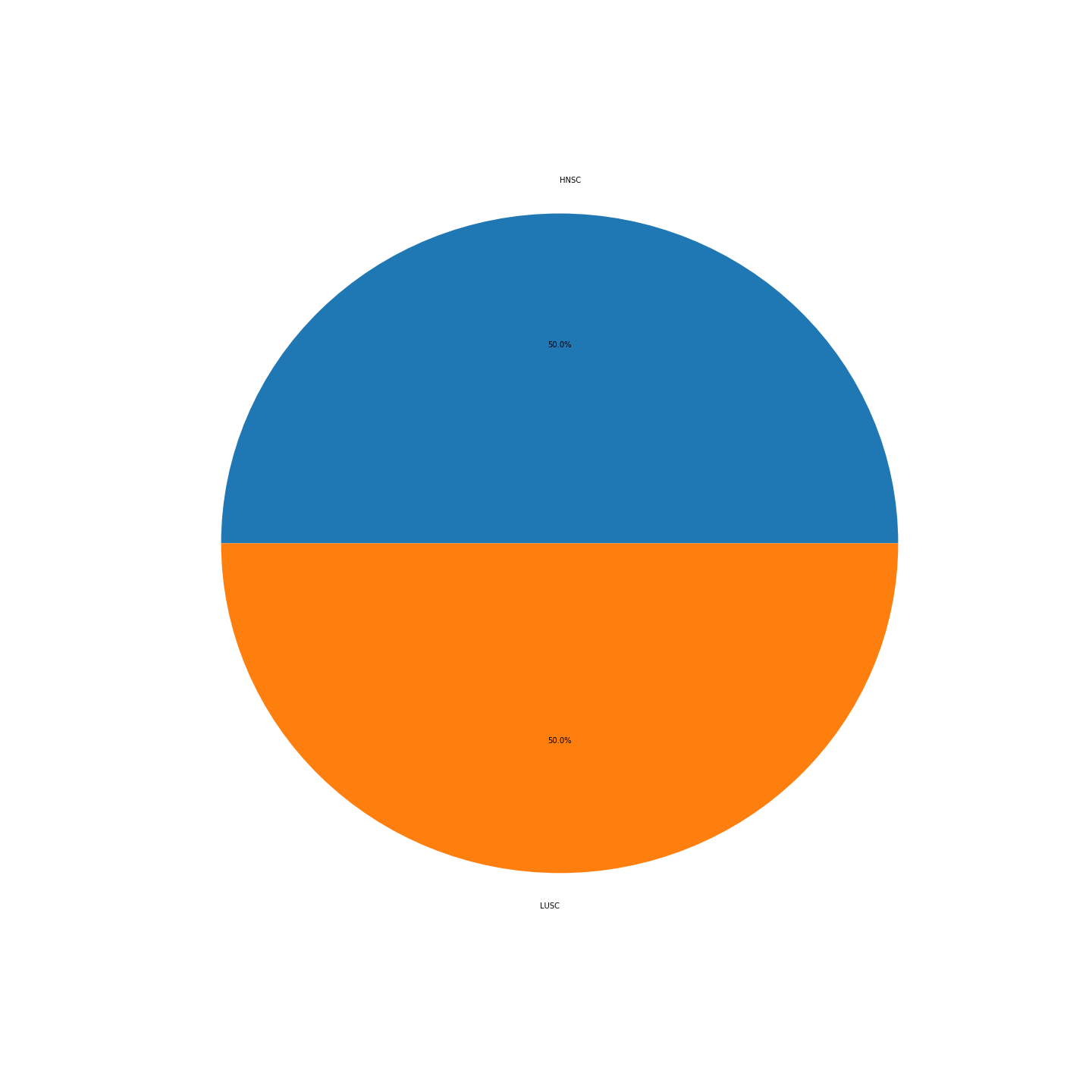


Supplementary Figure 26: THYM


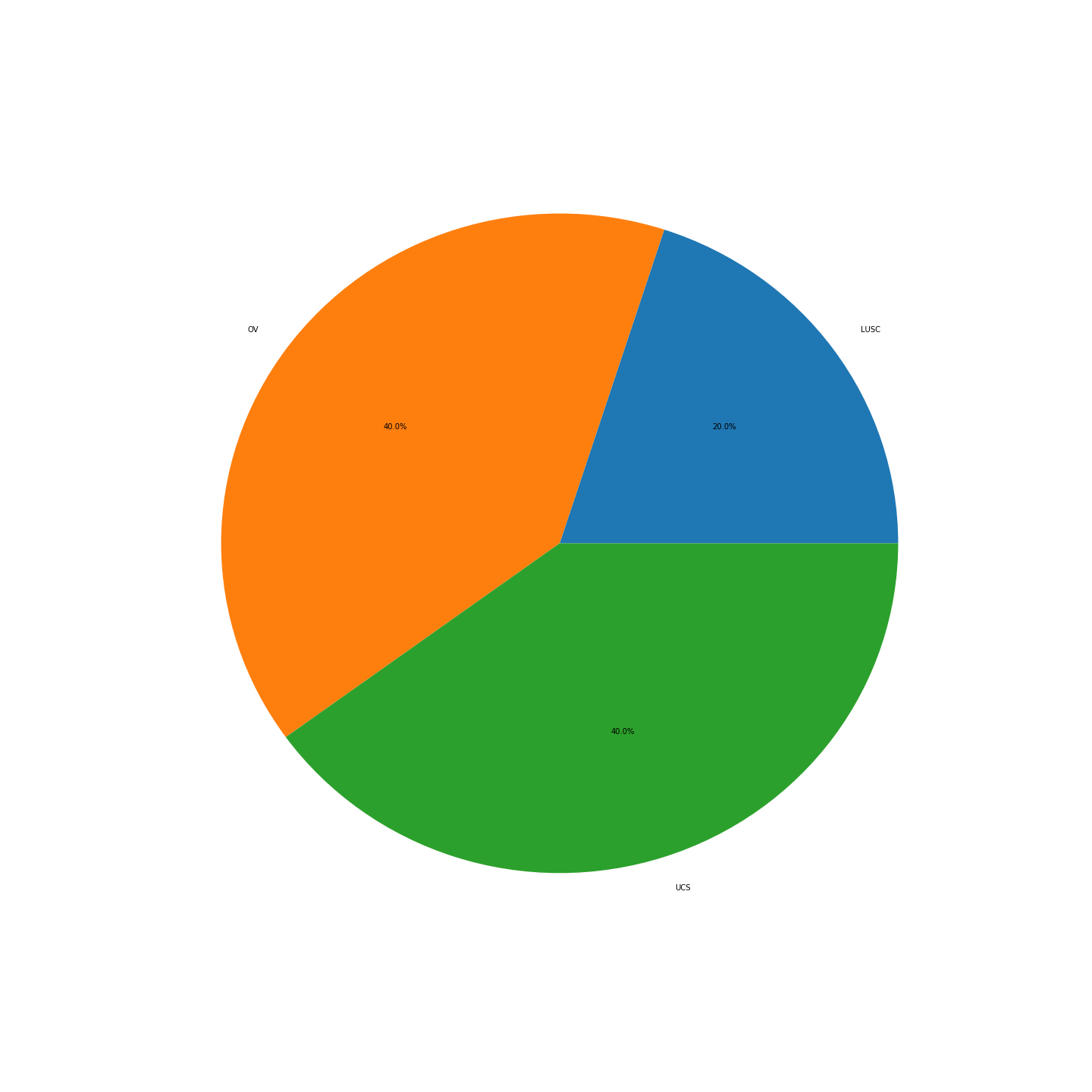


Supplementary Figure 27:UCEC


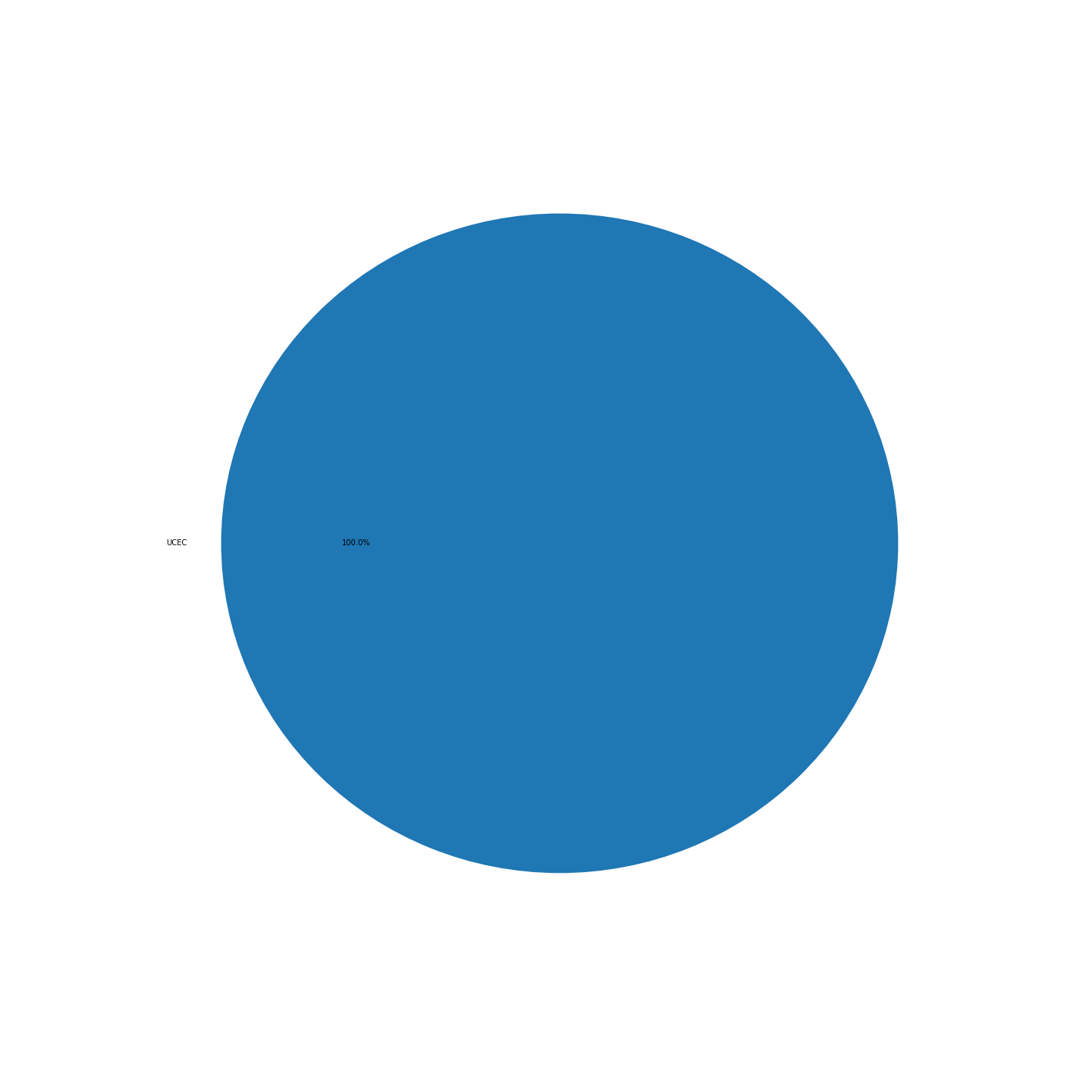


Supplementary Figure 28: UCS

Supplementary Figures 29 – 63: Latent space locations of each sample by class


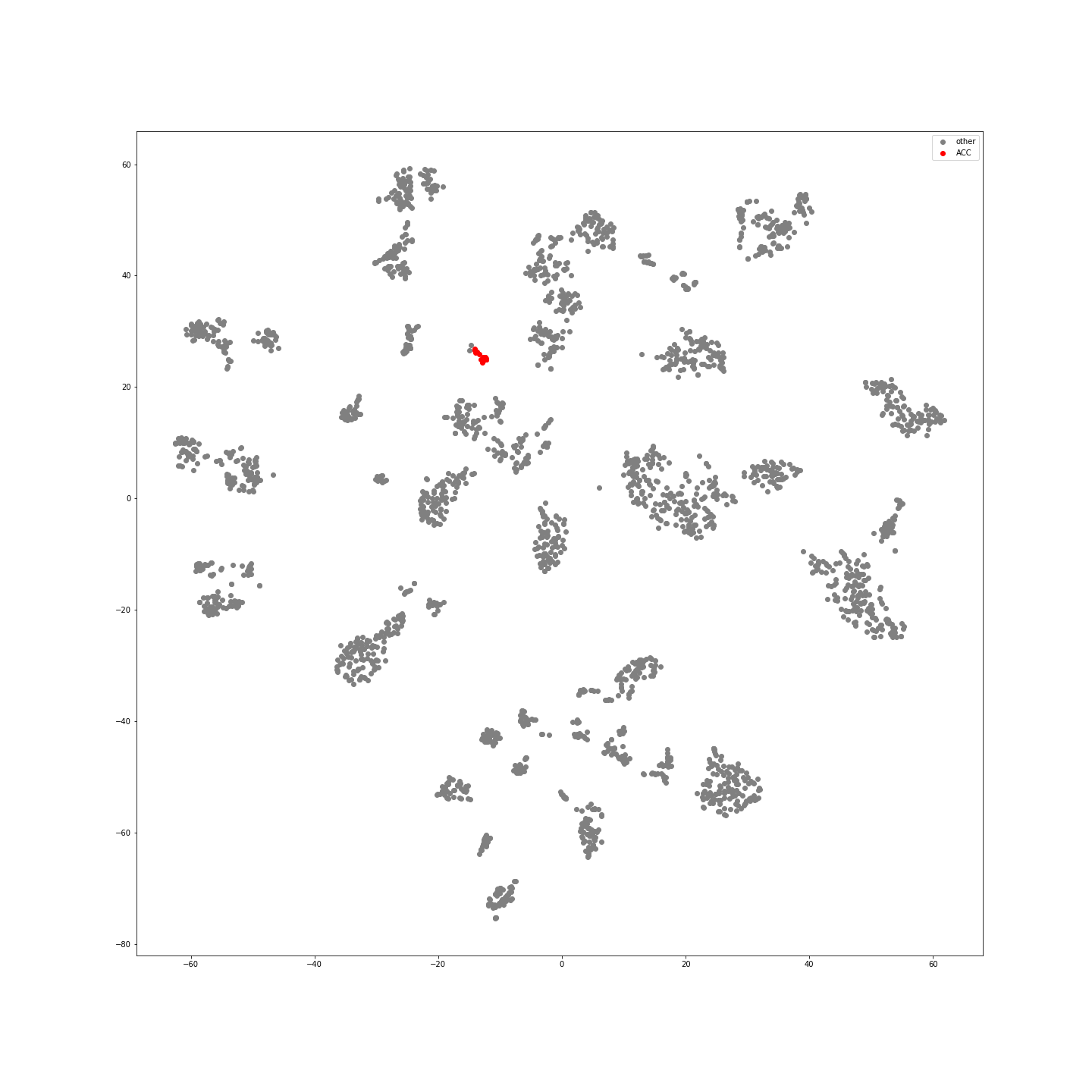


Supplementary Figure 29: ACC Samples in Latent Space


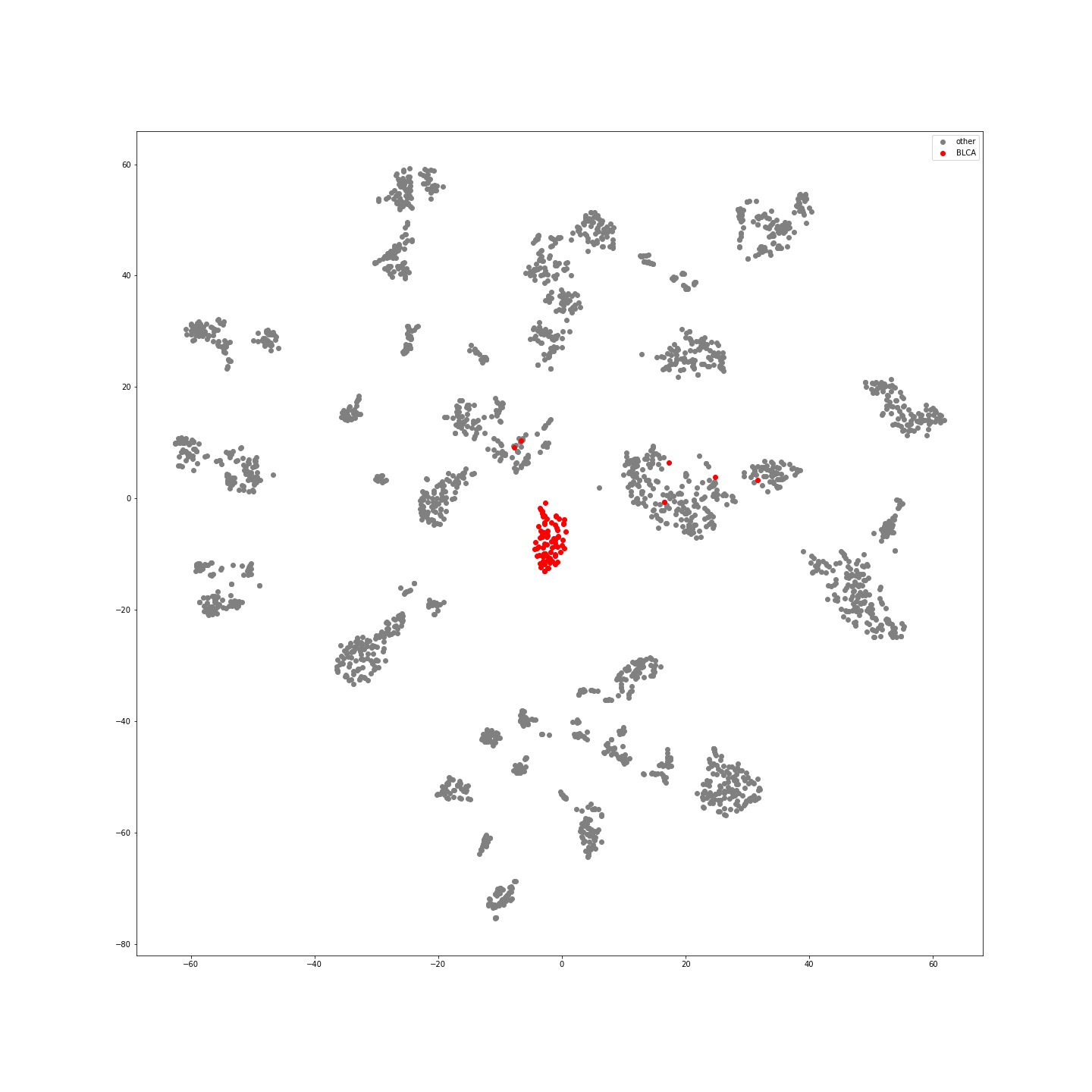


Supplementary Figure 30: BLCA Samples in Latent Space


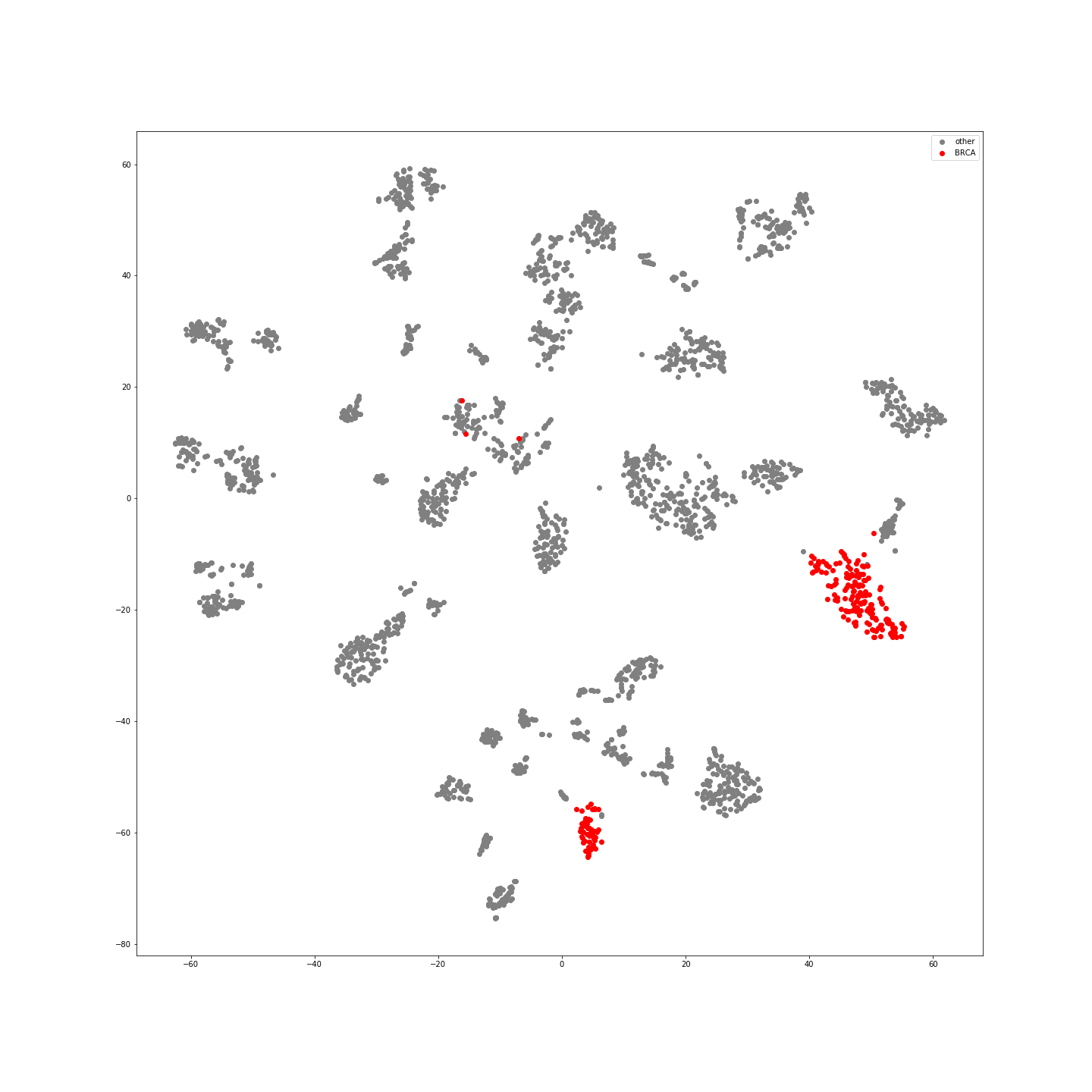


Supplementary Figure 31: BLCA Samples in Latent Space


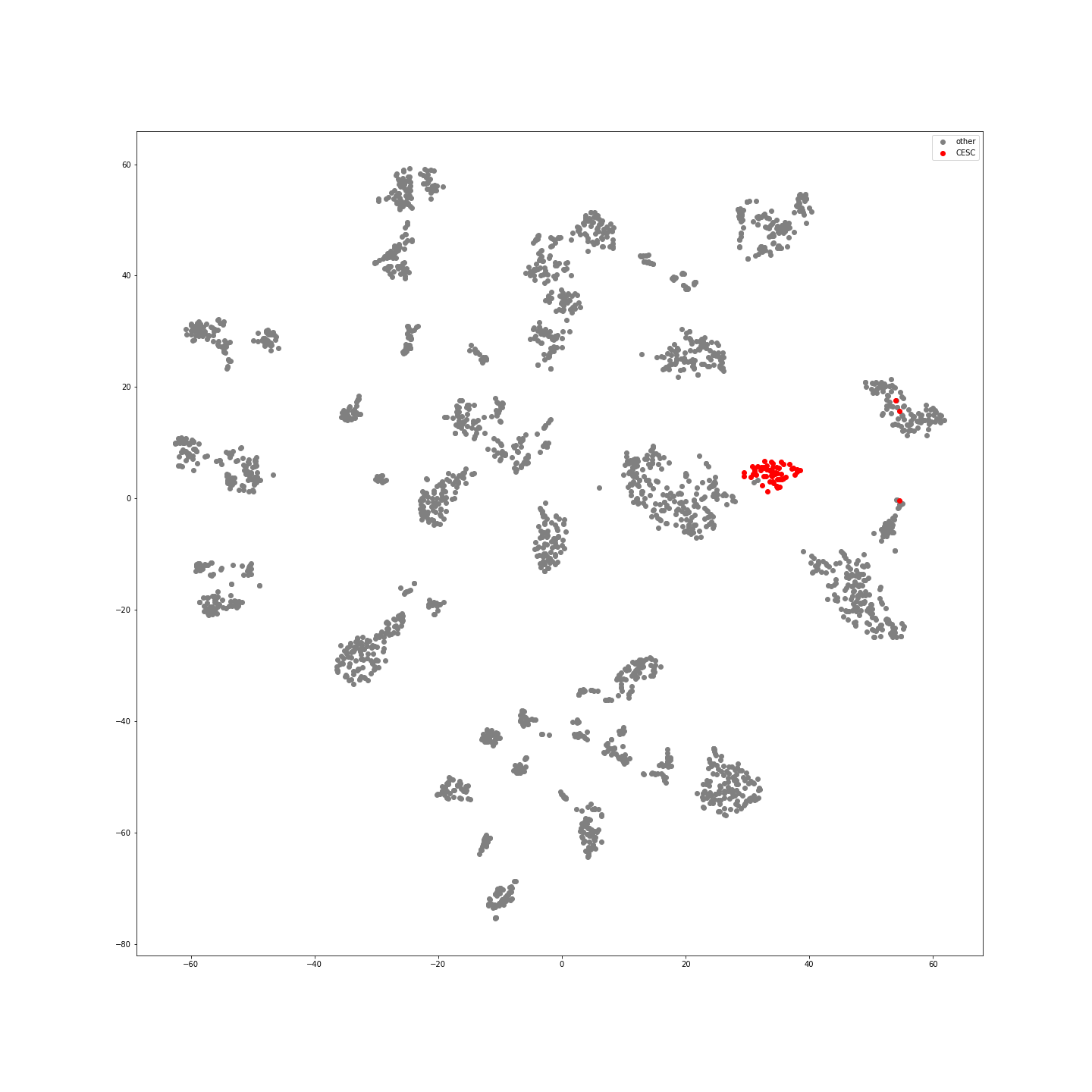


Supplementary Figure 32: CESC Samples in Latent Space


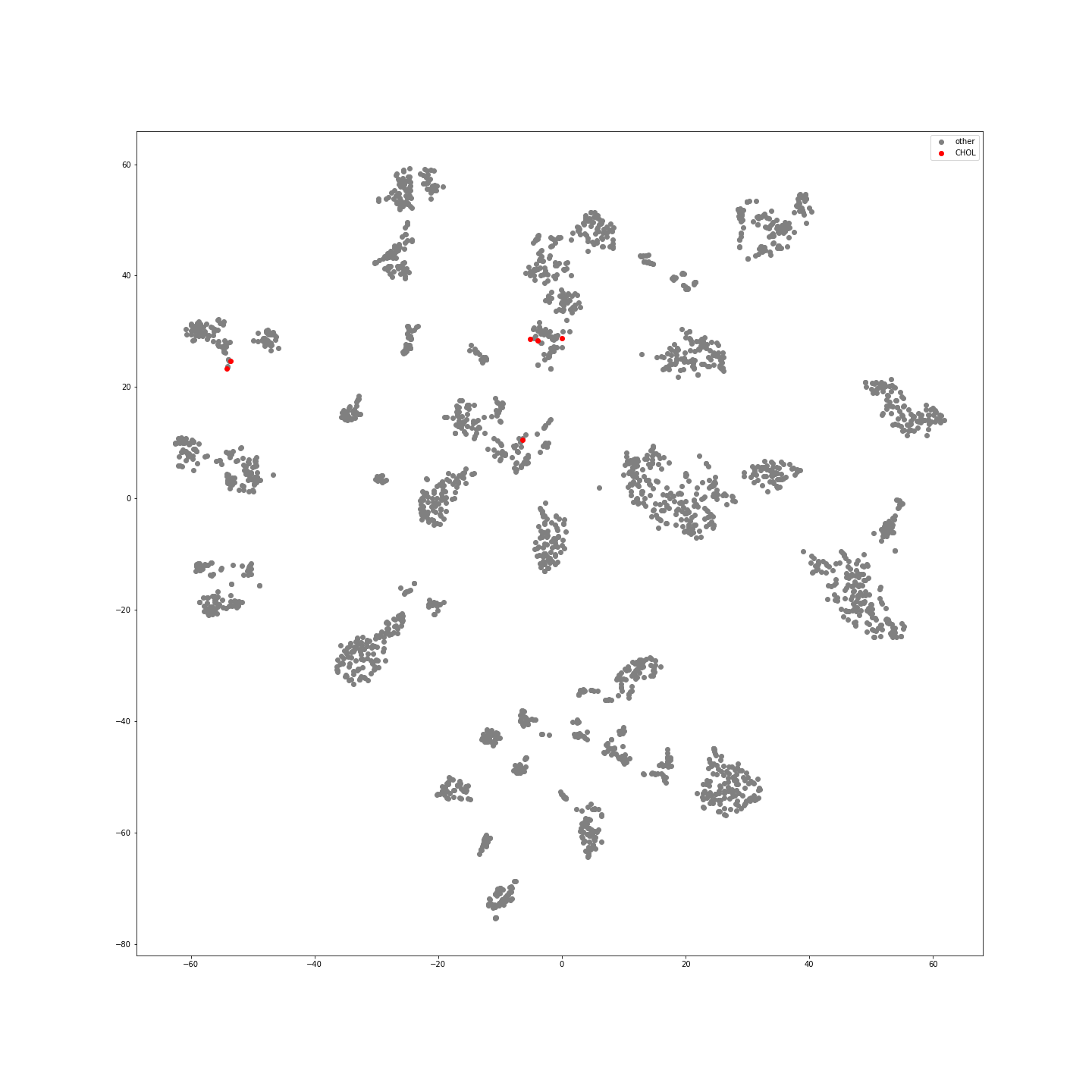


Supplementary Figure 33: CHOL Samples in Latent Space


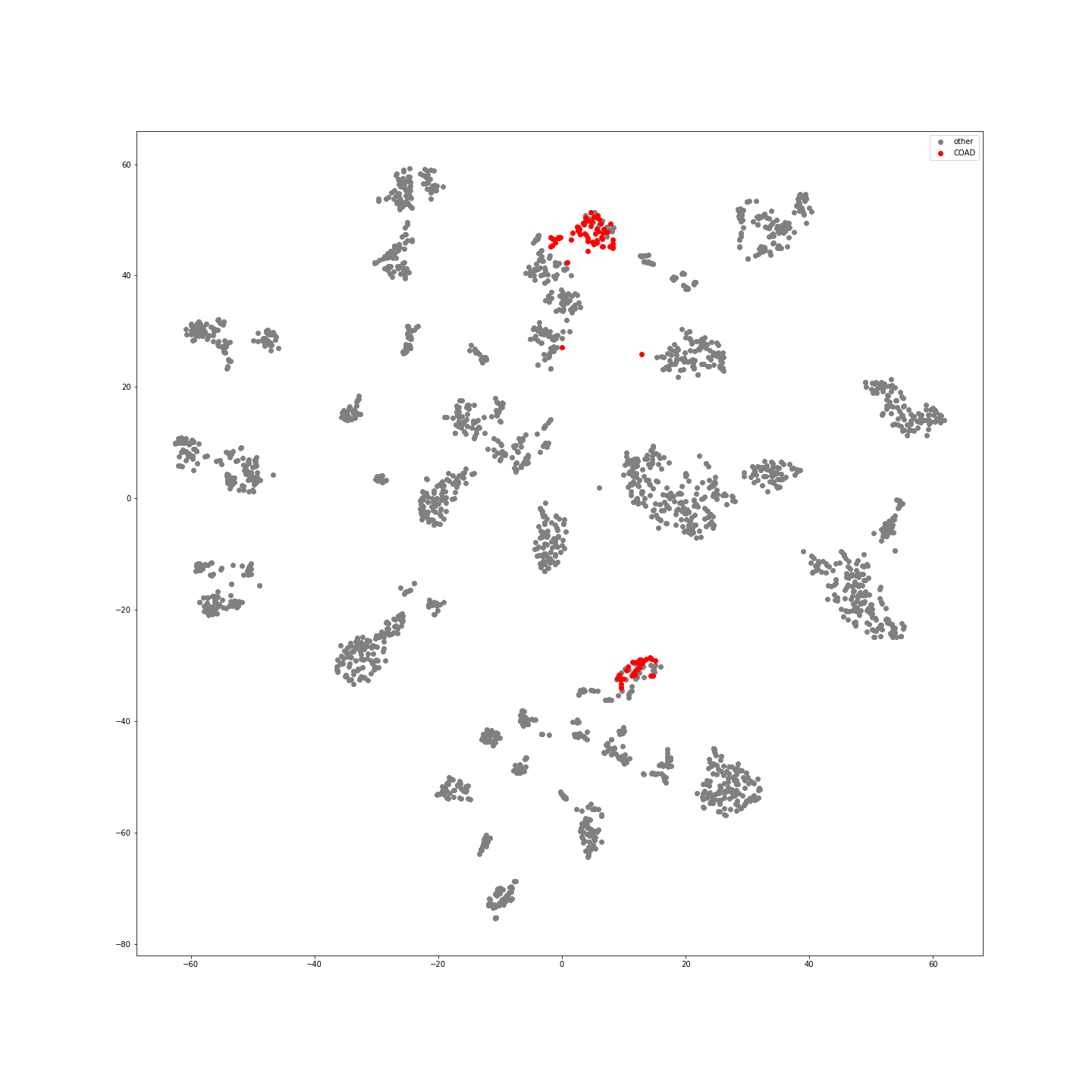


Supplementary Figure 34: COAD Samples in Latent Space


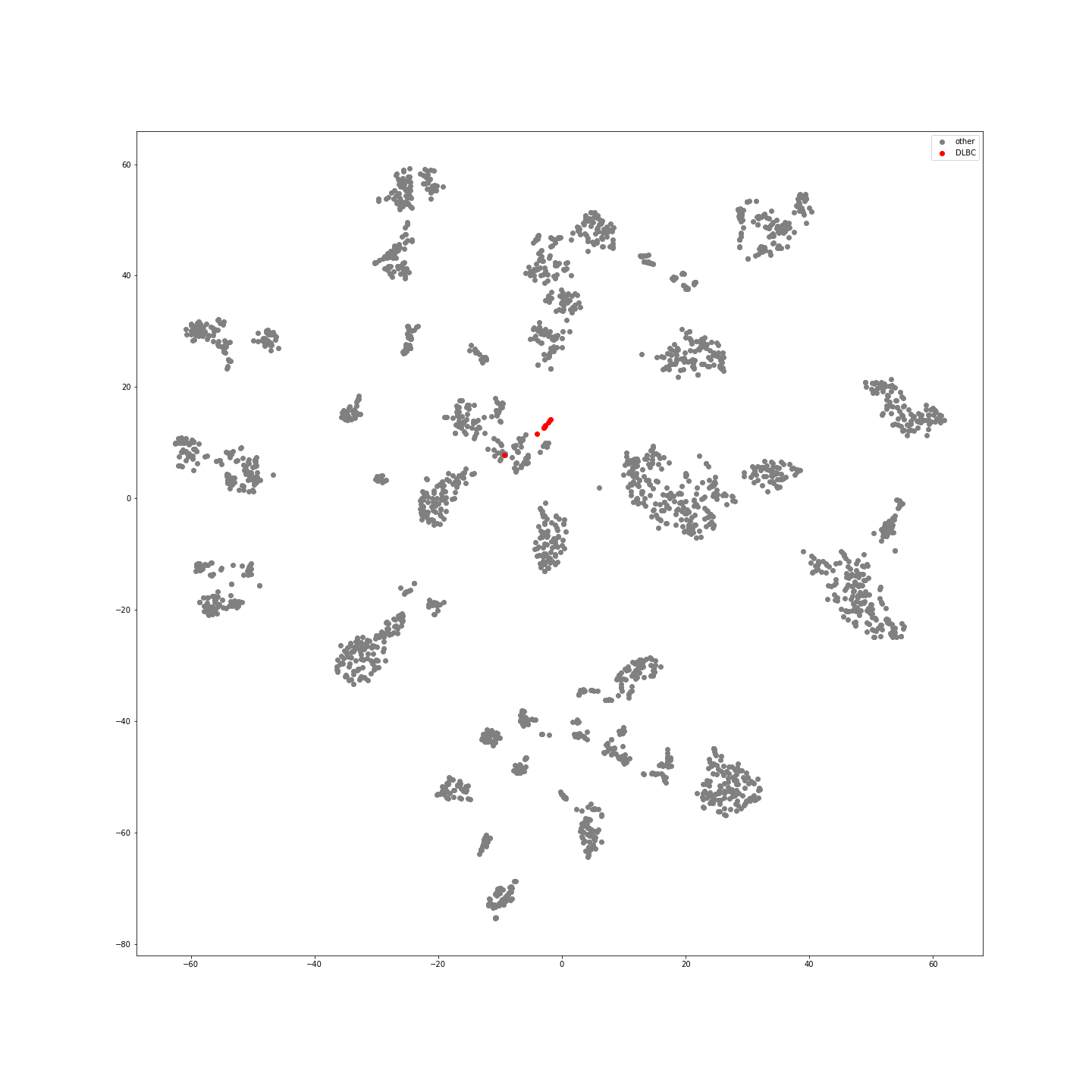


Supplementary Figure 35: DLBC Samples in Latent Space


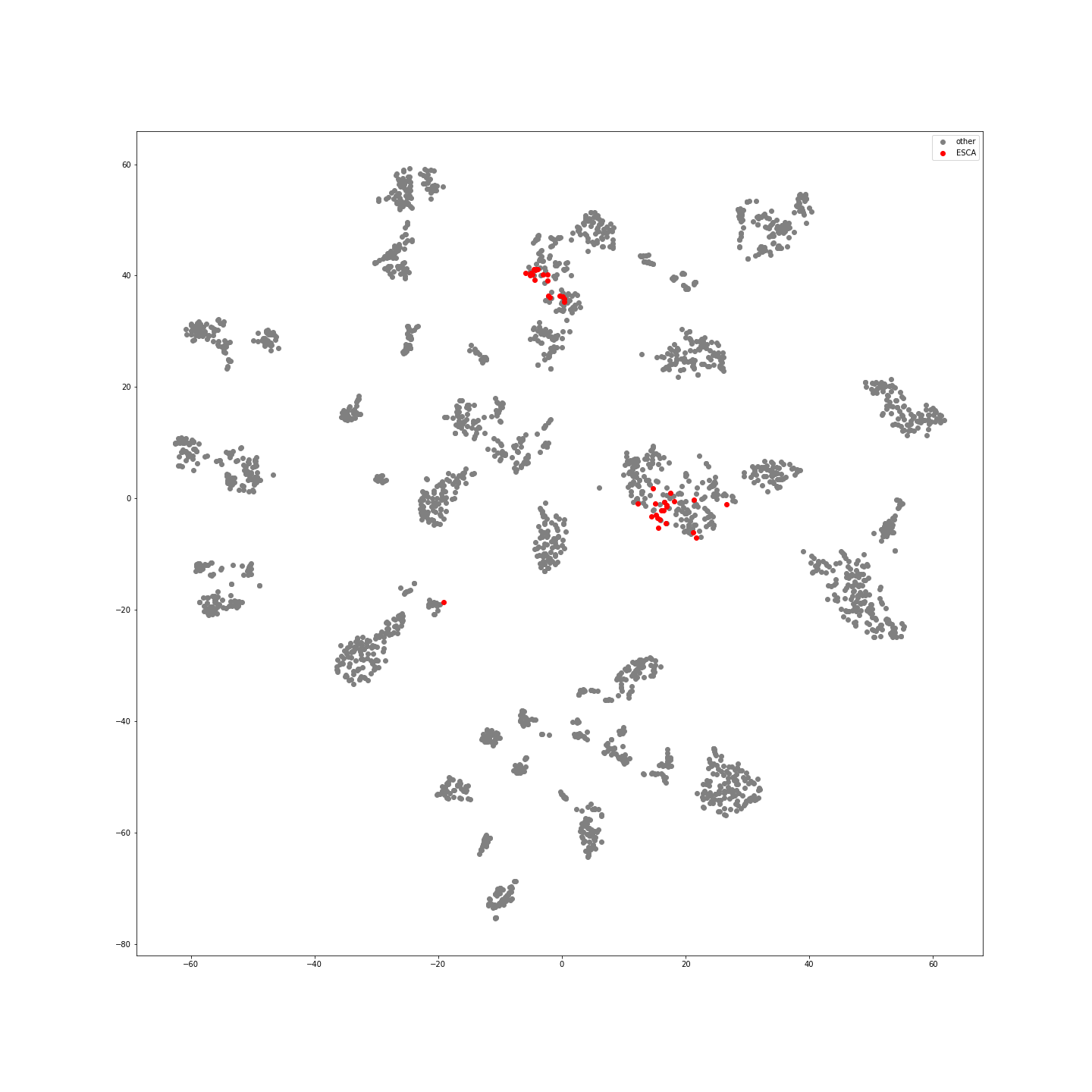


Supplementary Figure 36: ESCA Samples in Latent Space


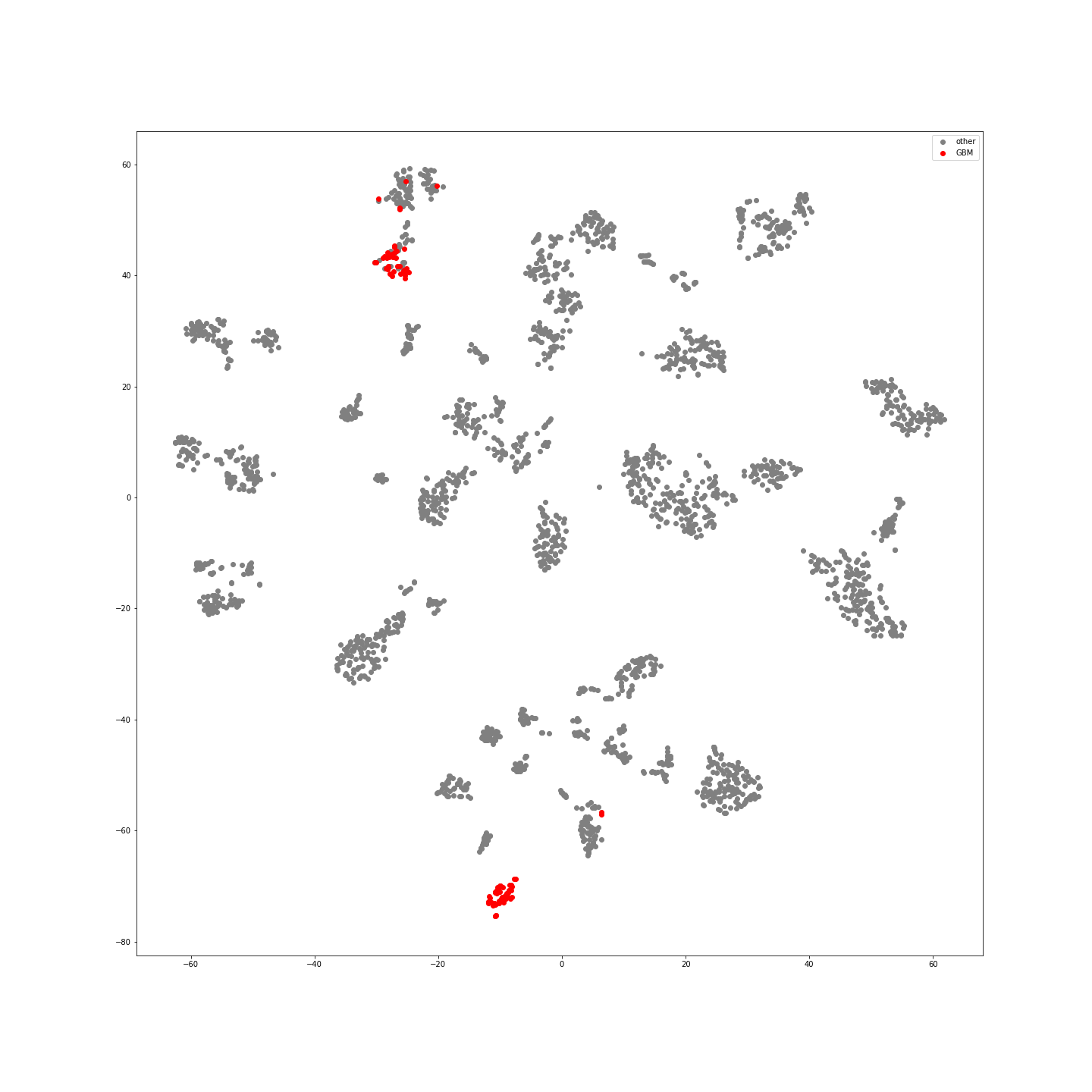


Supplementary Figure 37:GBM Samples in Latent Space


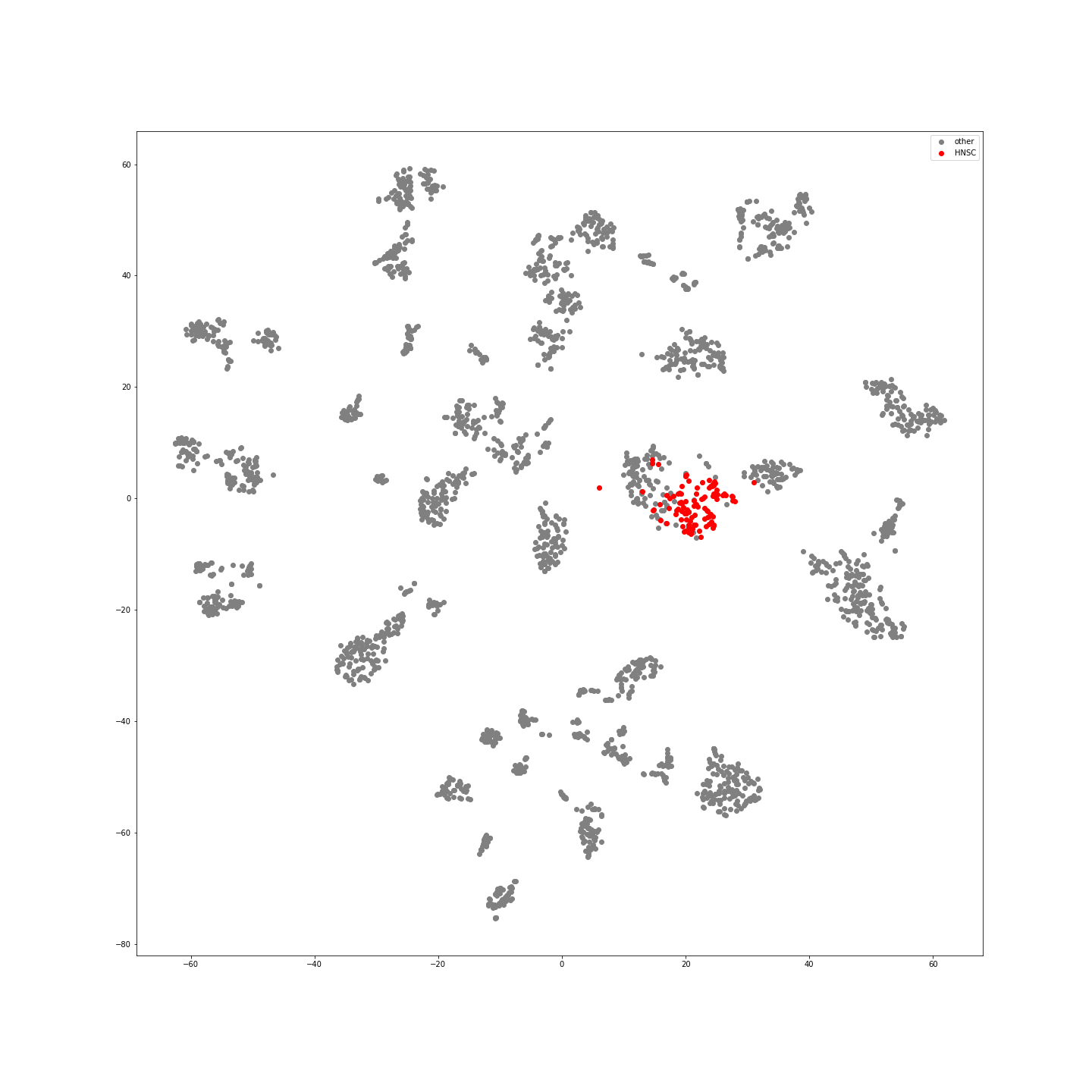


Supplementary Figure 38: HNSC Samples in Latent Space


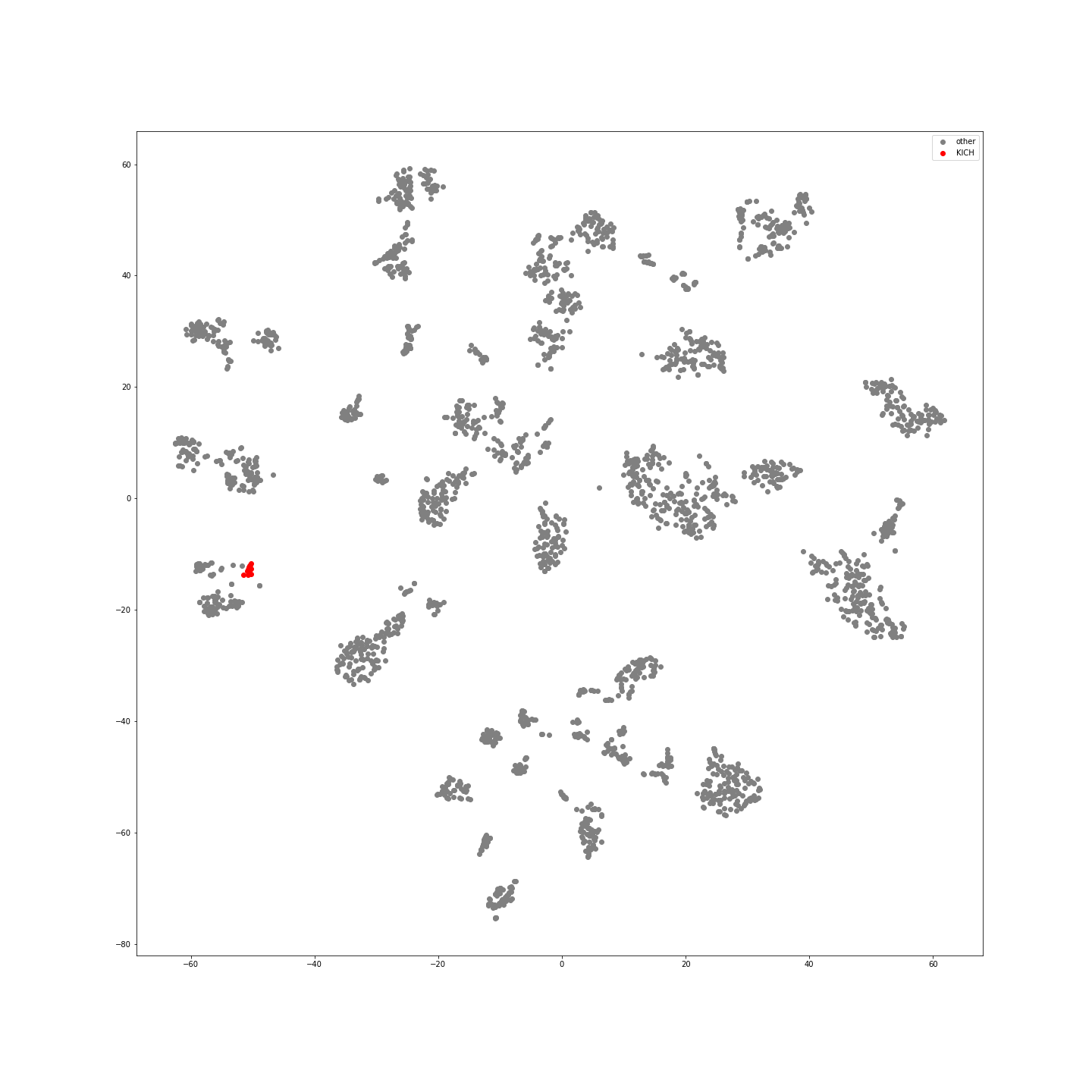


Supplementary Figure 39:KCH Samples in Latent Space


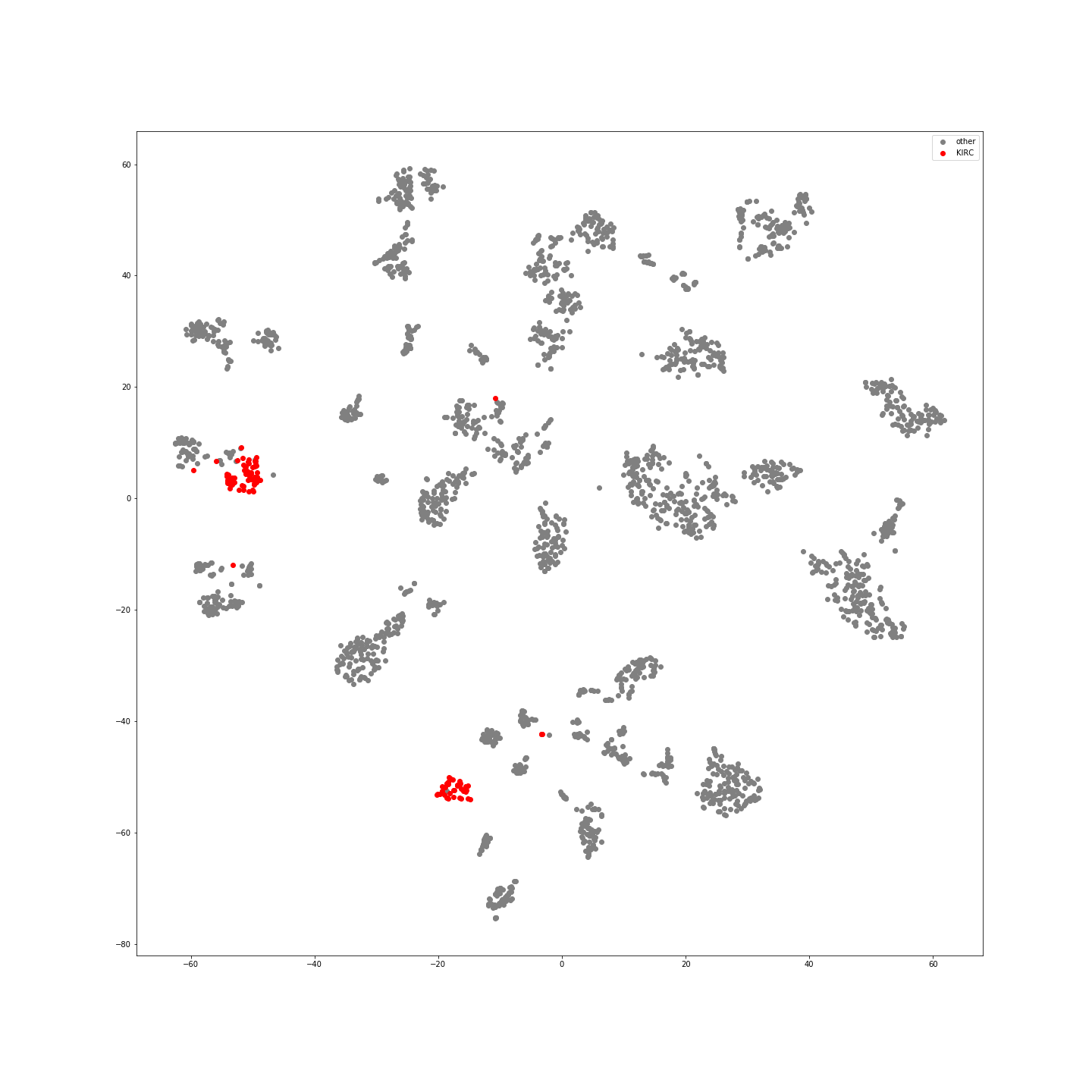


Supplementary Figure 40:KIRC Samples in Latent Space


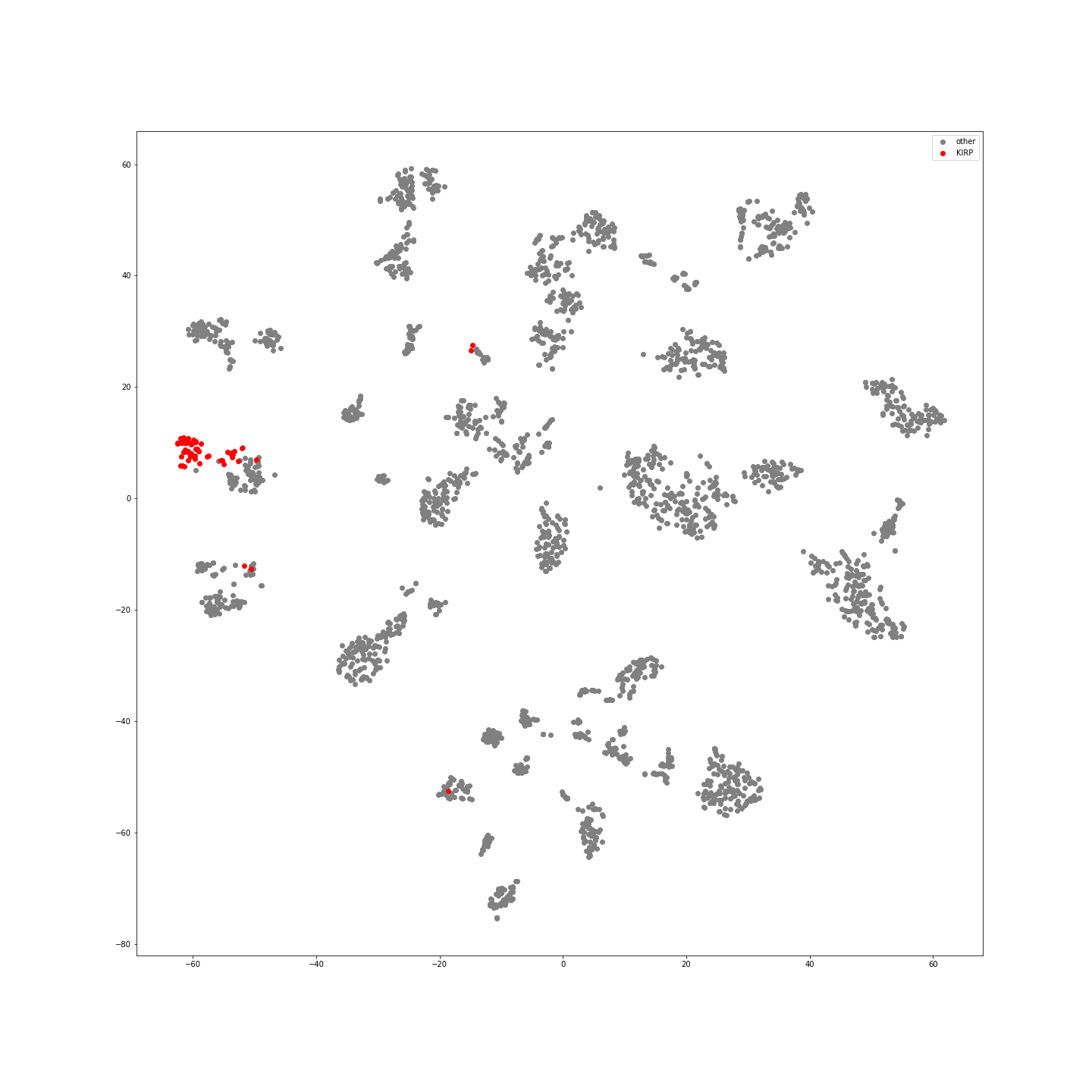


Supplementary Figure 41: KIRP Samples in Latent Space


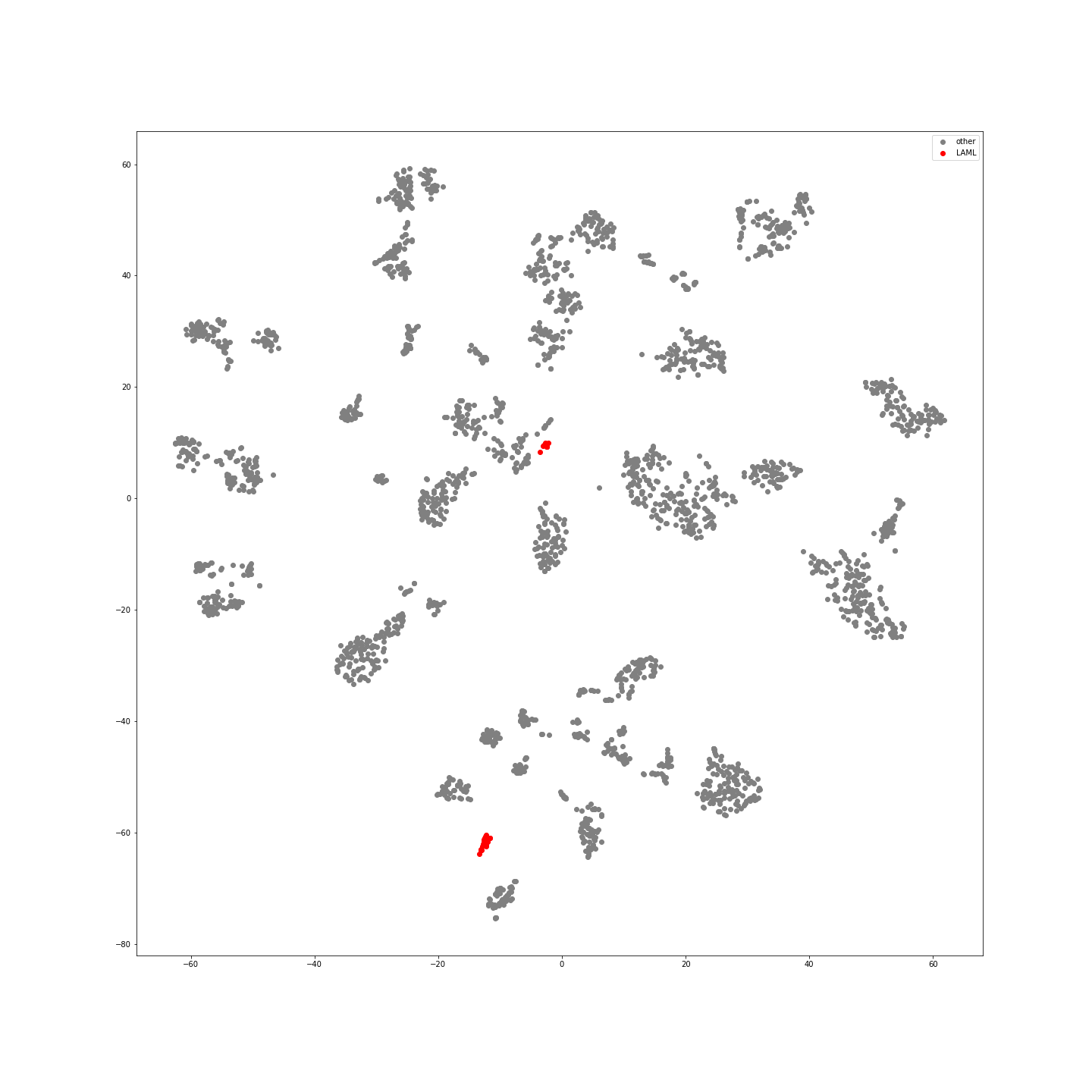


Supplementary Figure 42: LAML Samples in Latent Space


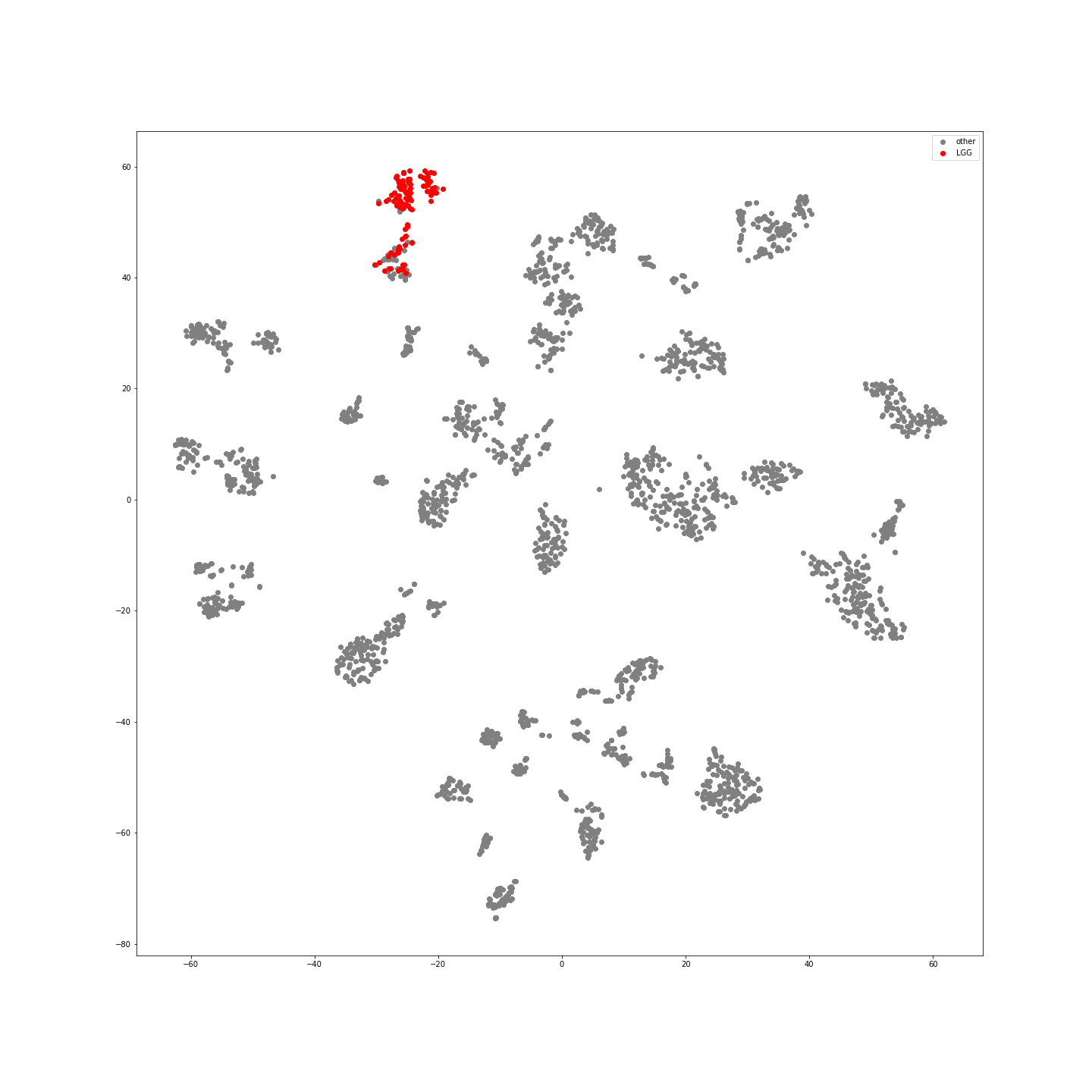


Supplementary Figure 43: LGG Samples in Latent Space


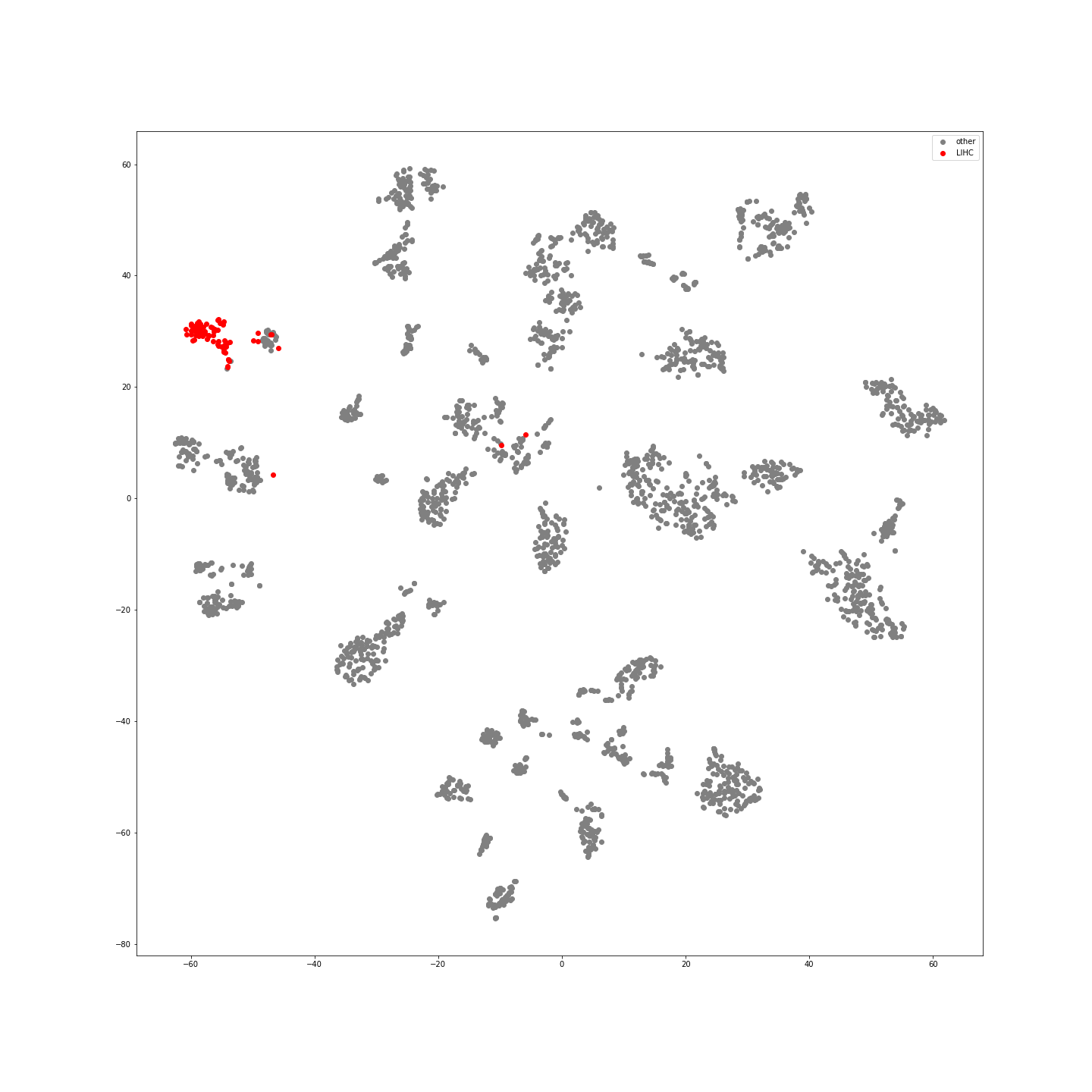


Supplementary Figure 44: LIHC Samples in Latent Space


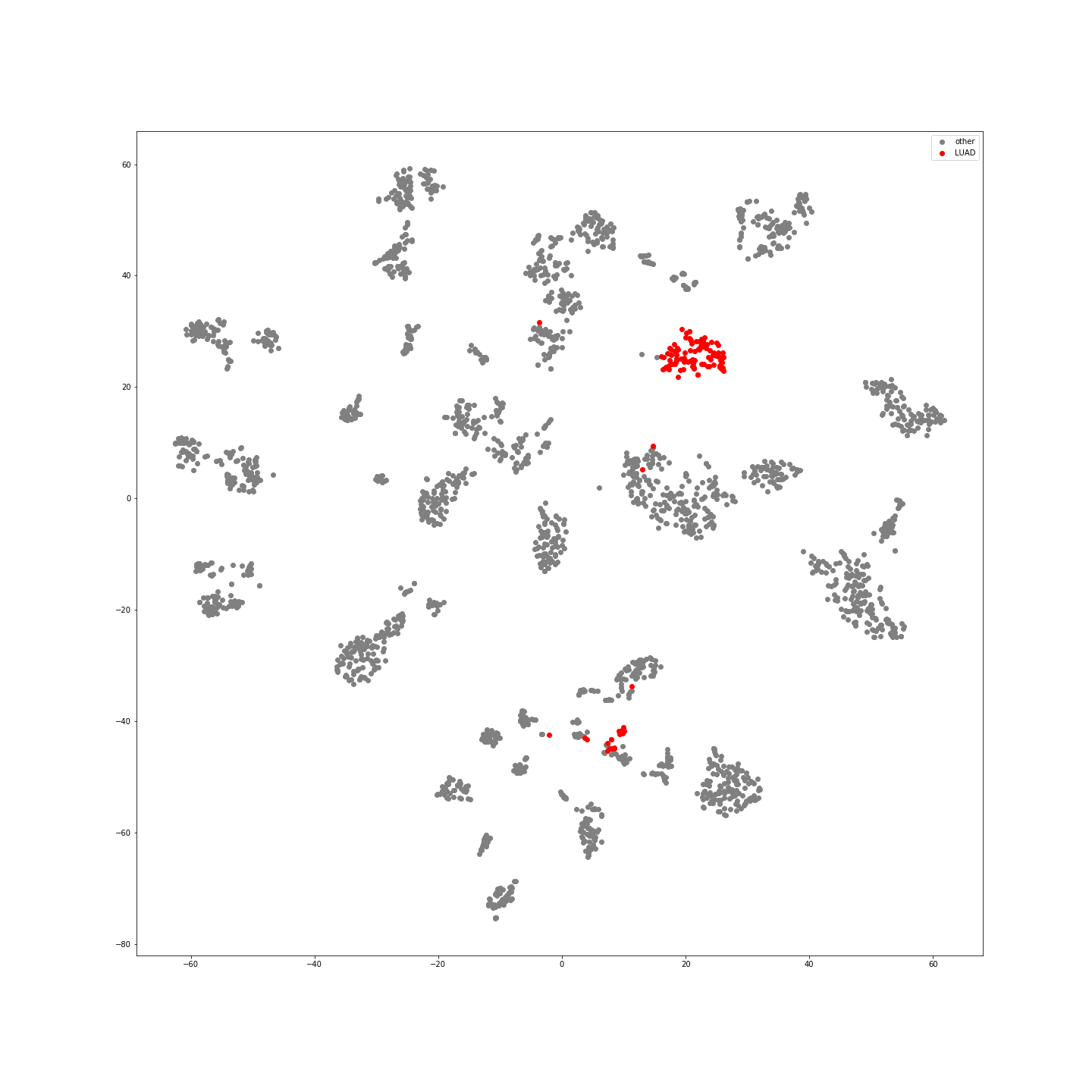


Supplementary Figure 45: LUAD Samples in Latent Space


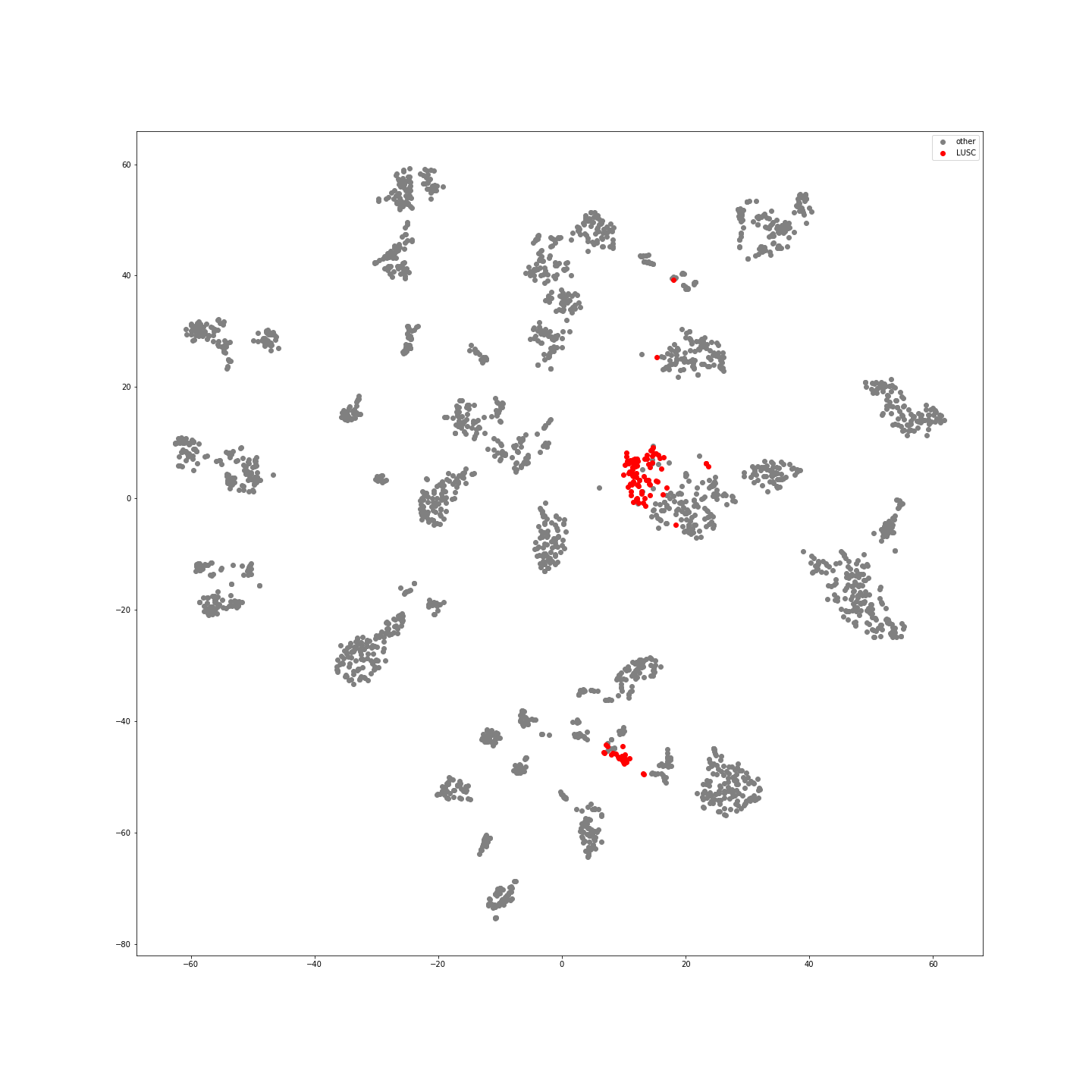


Supplementary Figure 46: LUSC Samples in Latent Space


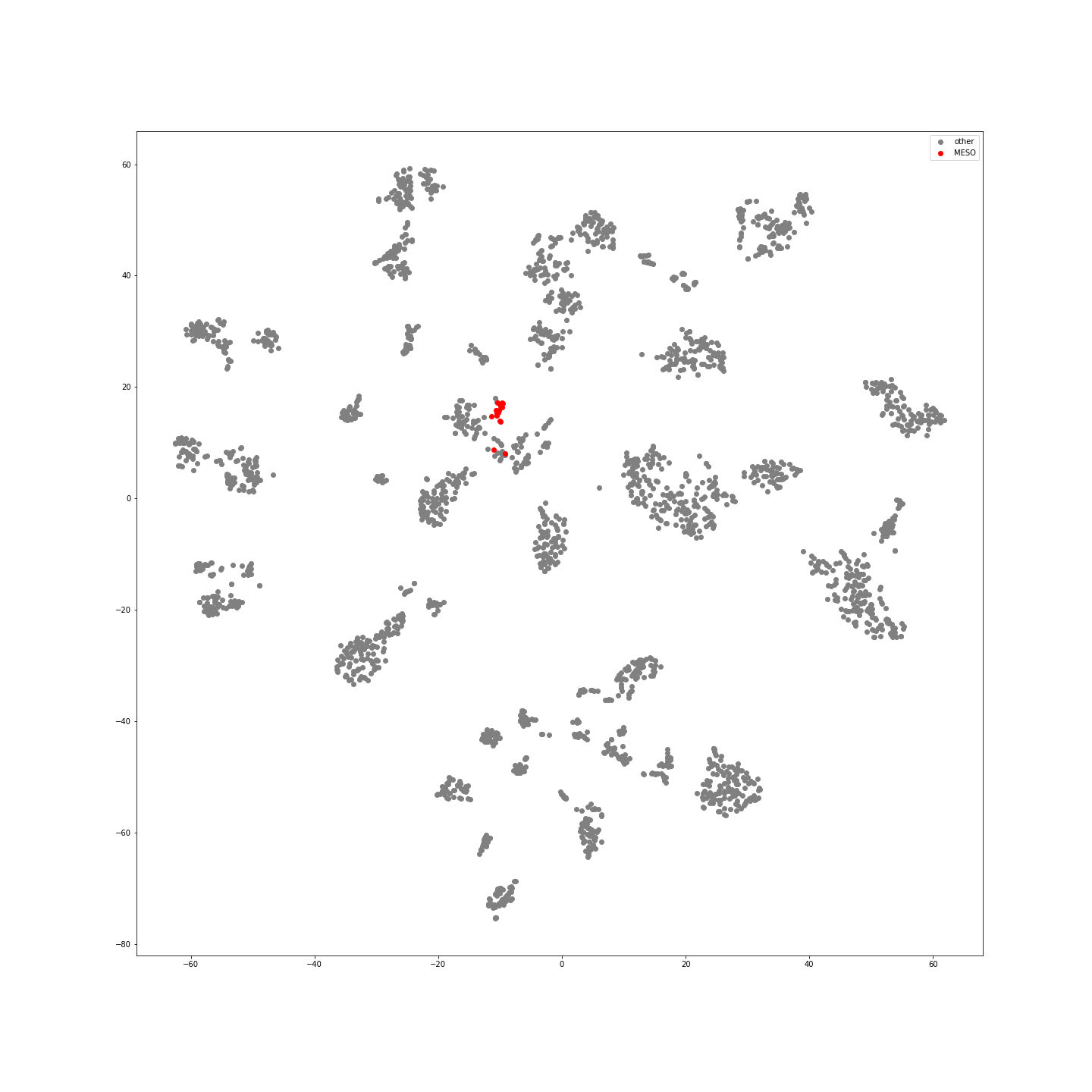


Supplementary Figure 47: MESO Samples in Latent Space


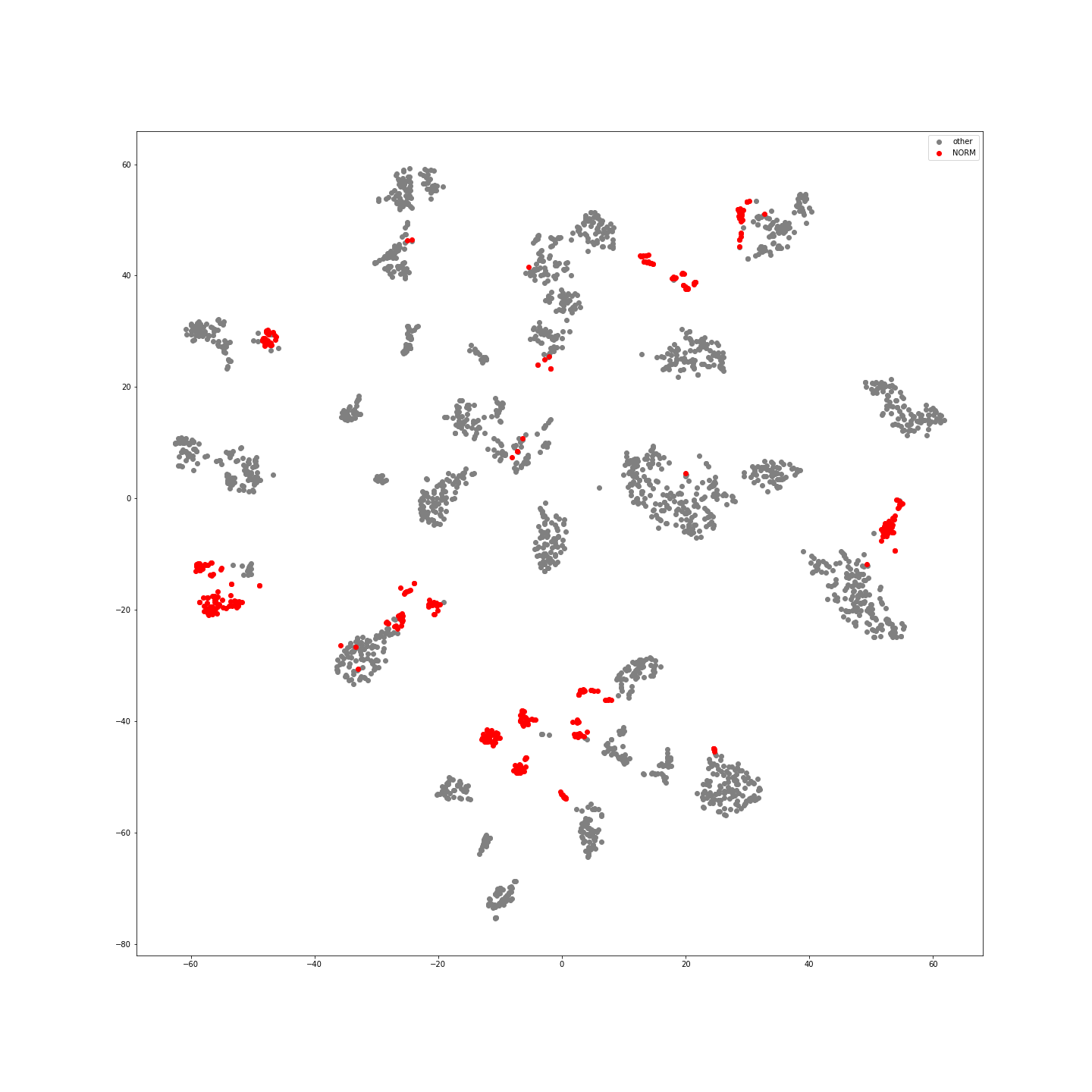


Supplementary Figure 48: NORM Samples in Latent Space


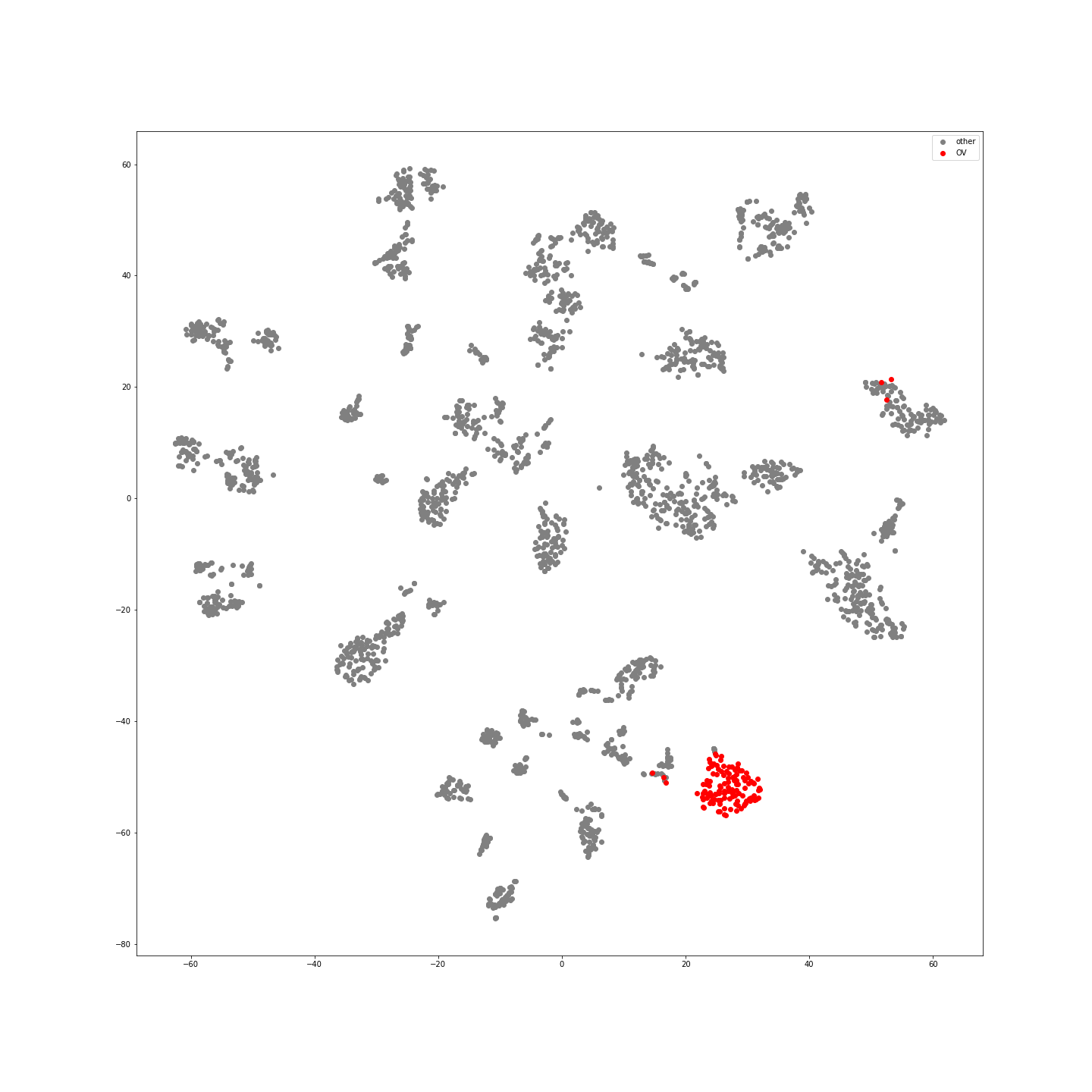


Supplementary Figure 49: OV Samples in Latent Space


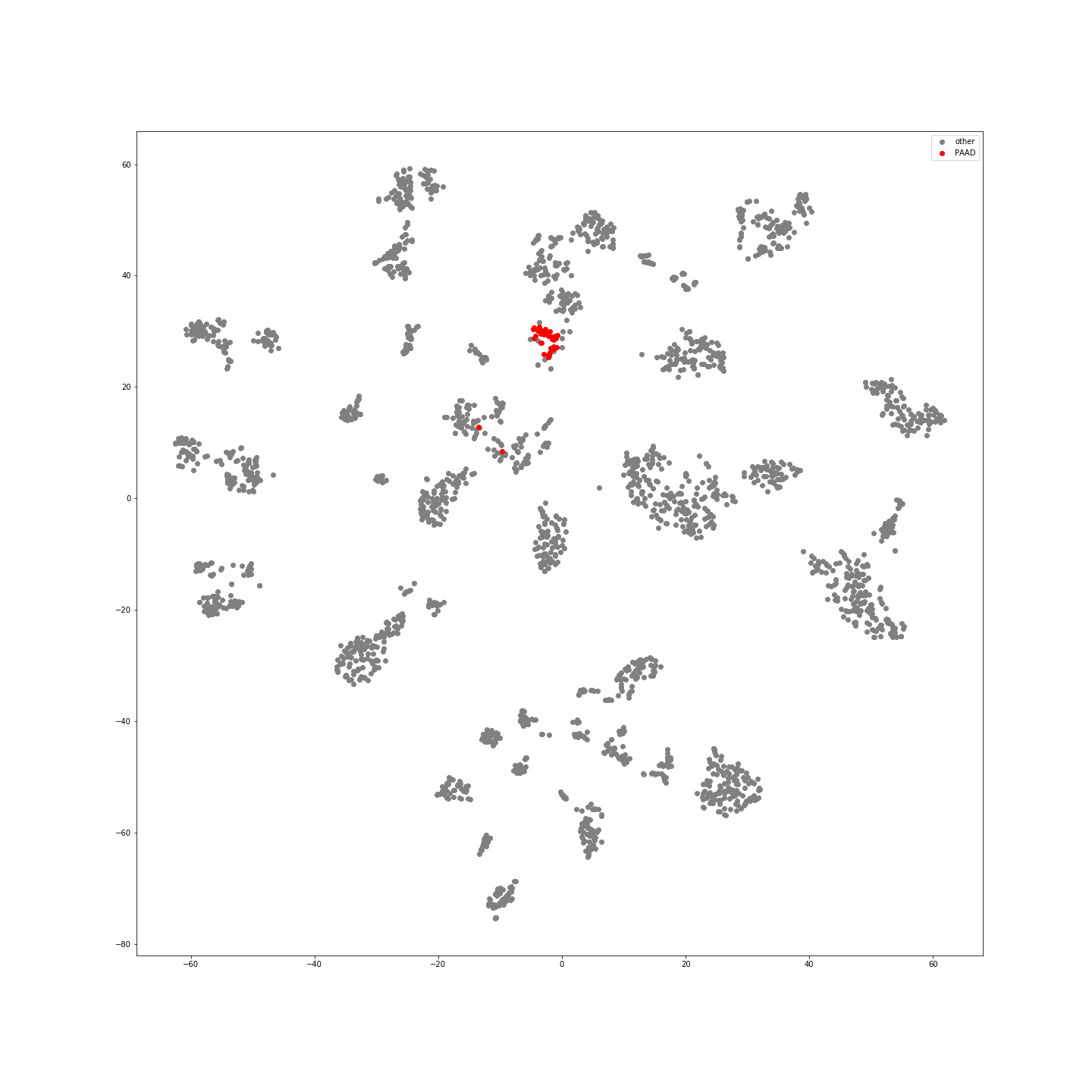


Supplementary Figure 50: PAAD Samples in Latent Space


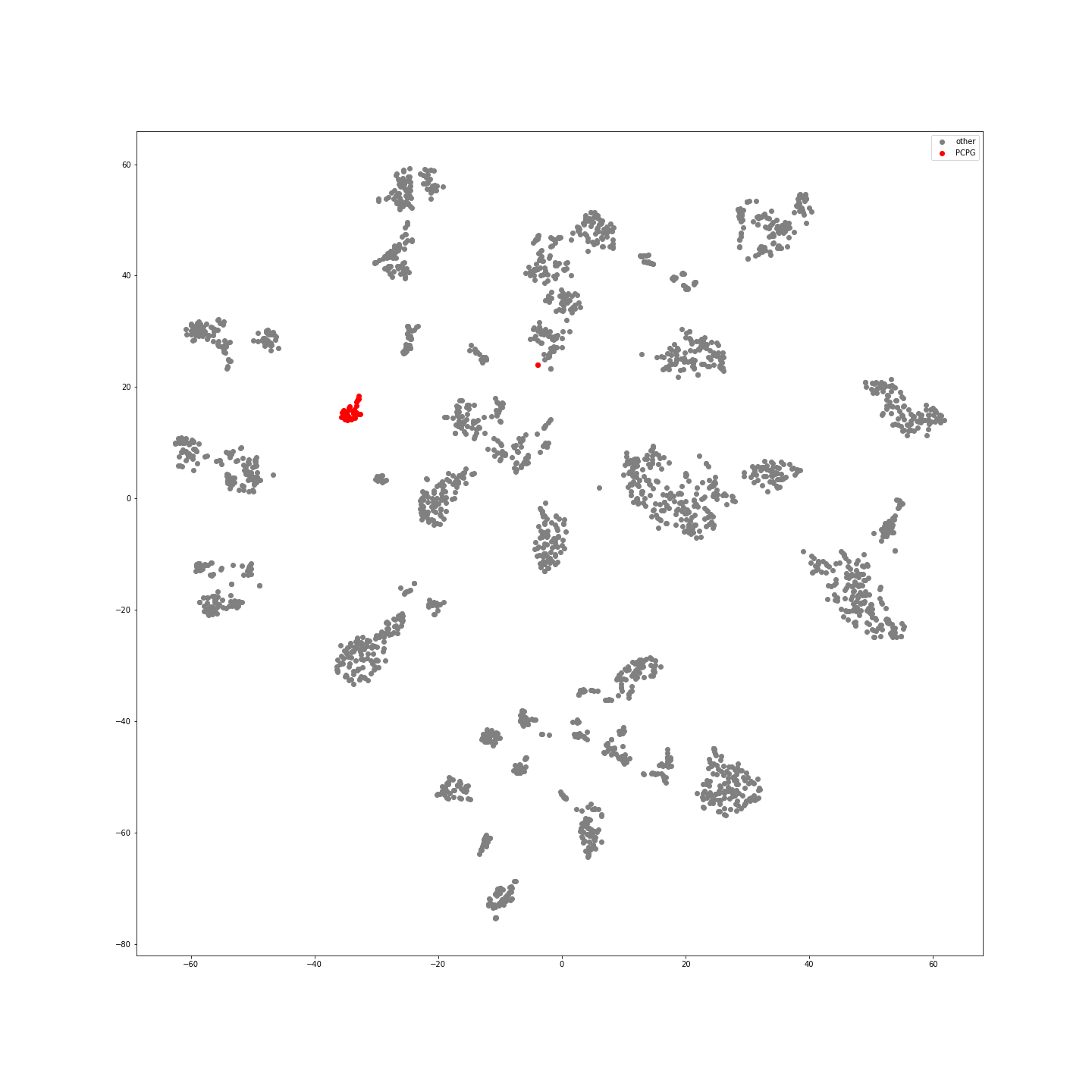


Supplementary Figure 51:PCPG Samples in Latent Space


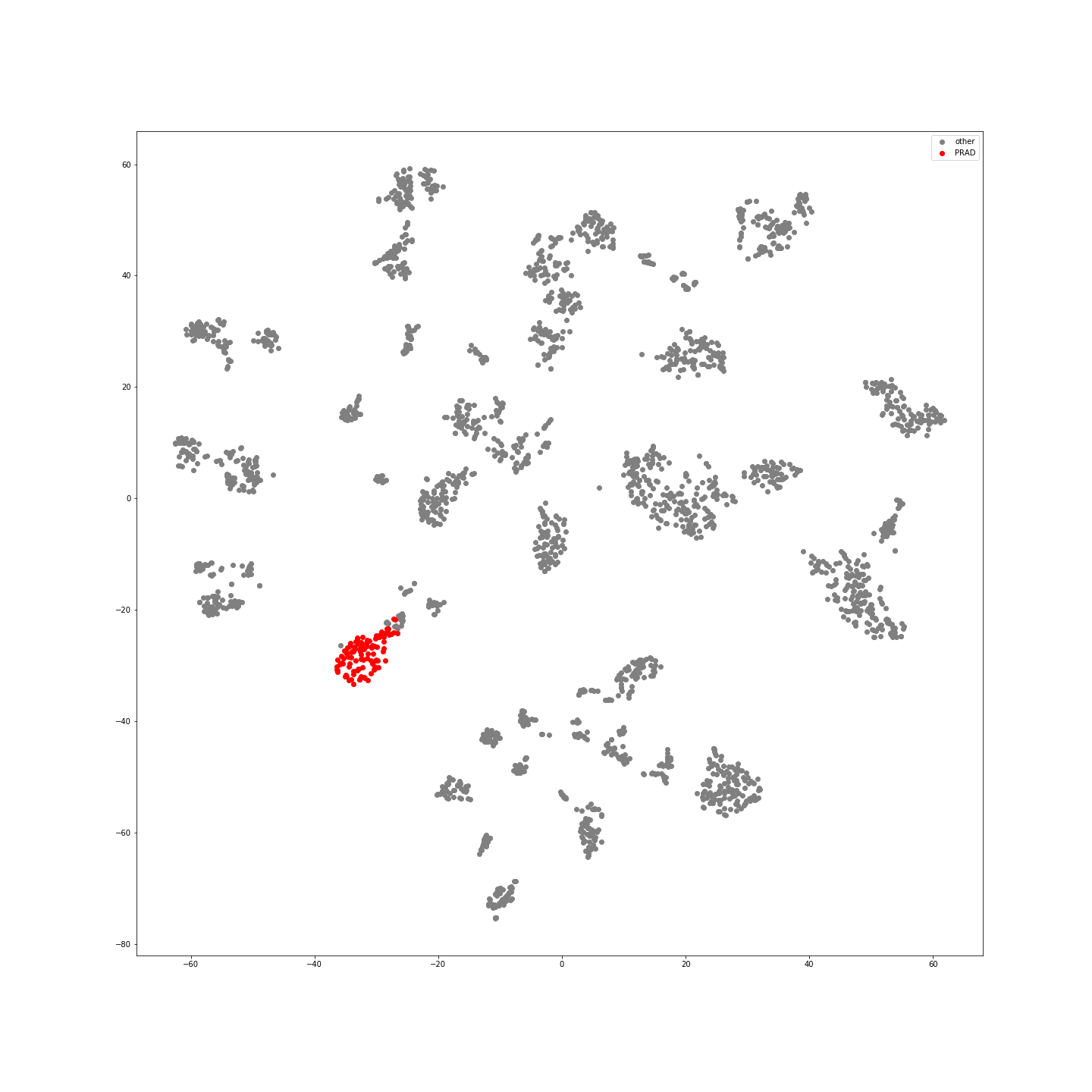


Supplementary Figure 52: PRAD Samples in Latent Space


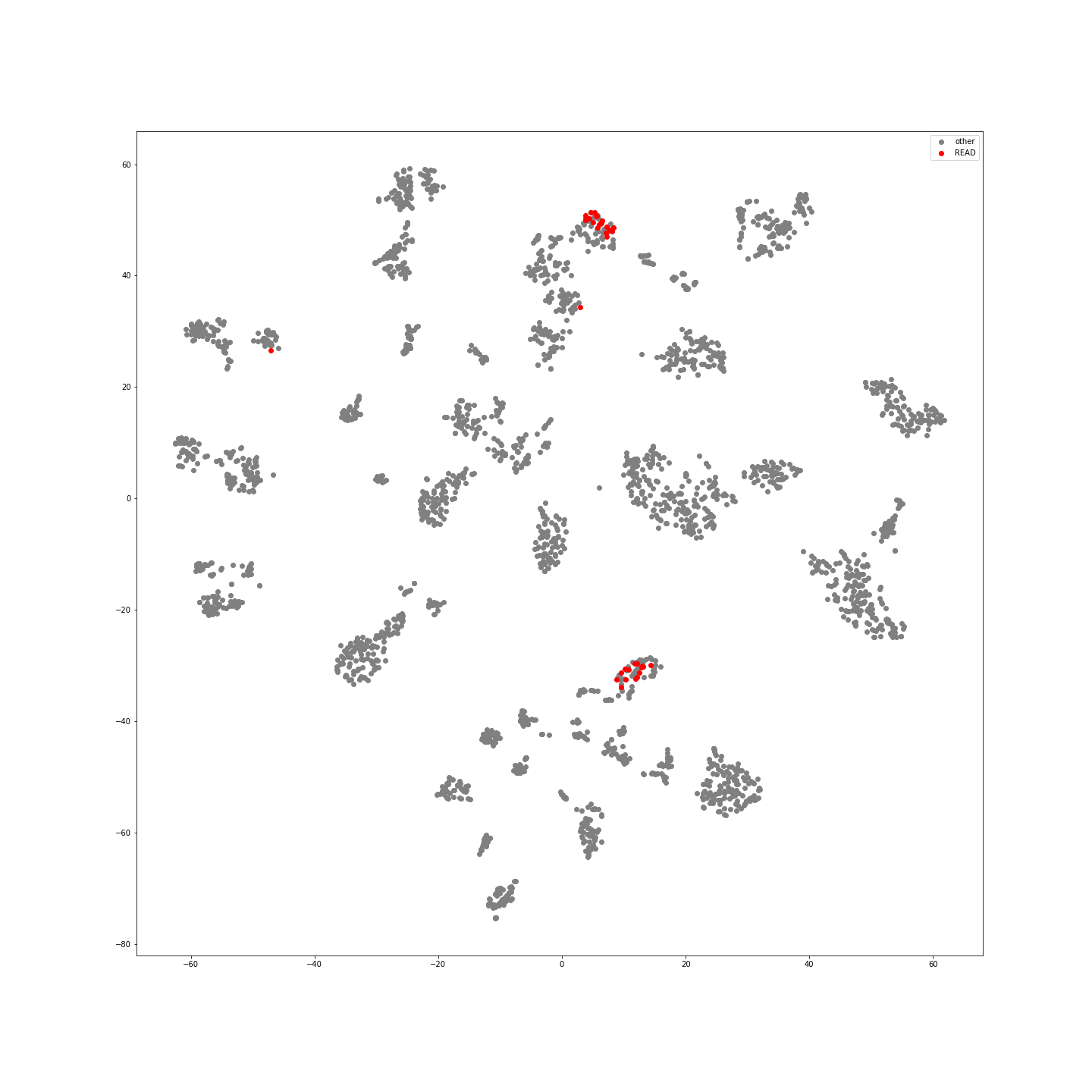


Supplementary Figure 53: READ Samples in Latent Space


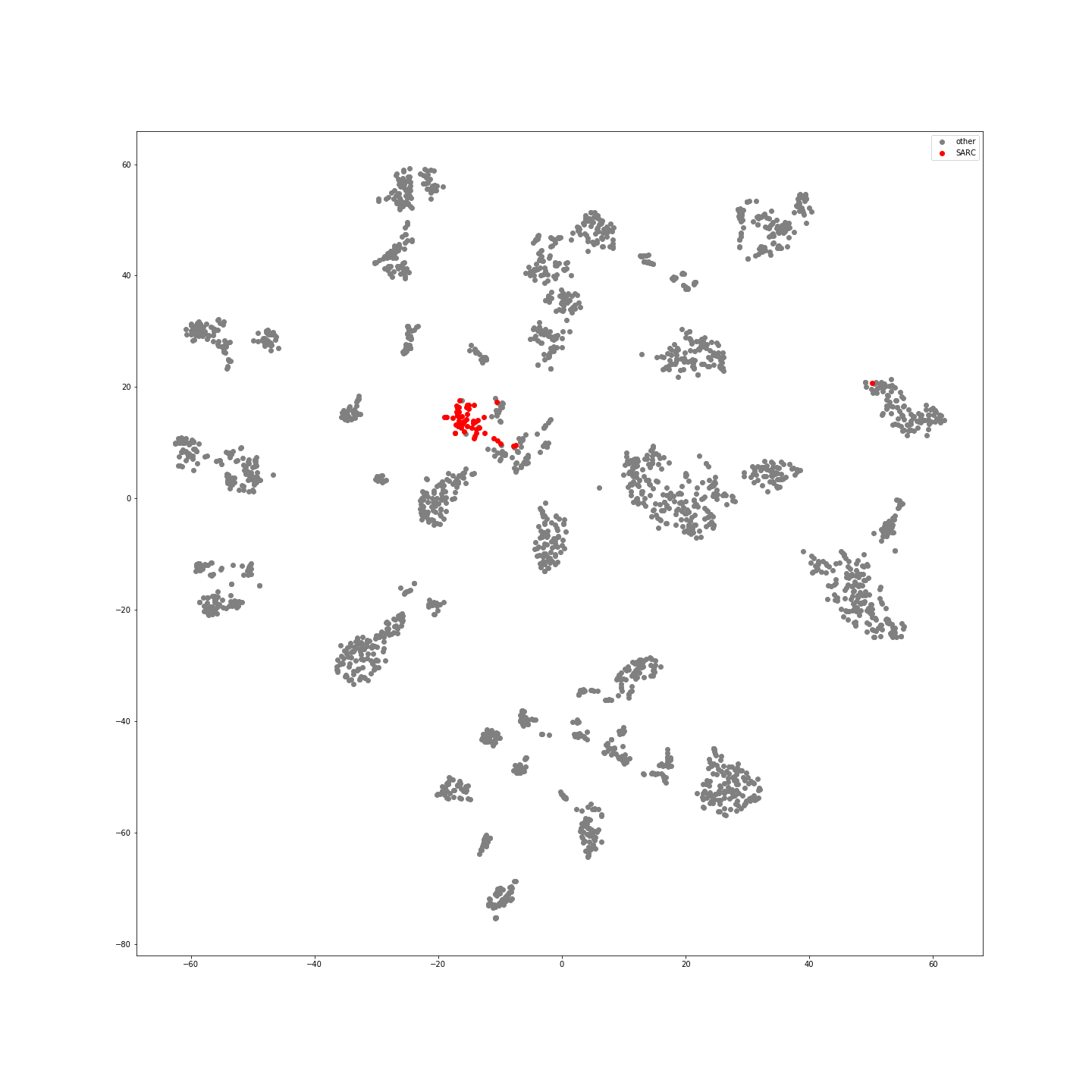


Supplementary Figure 54: SARC Samples in Latent Space


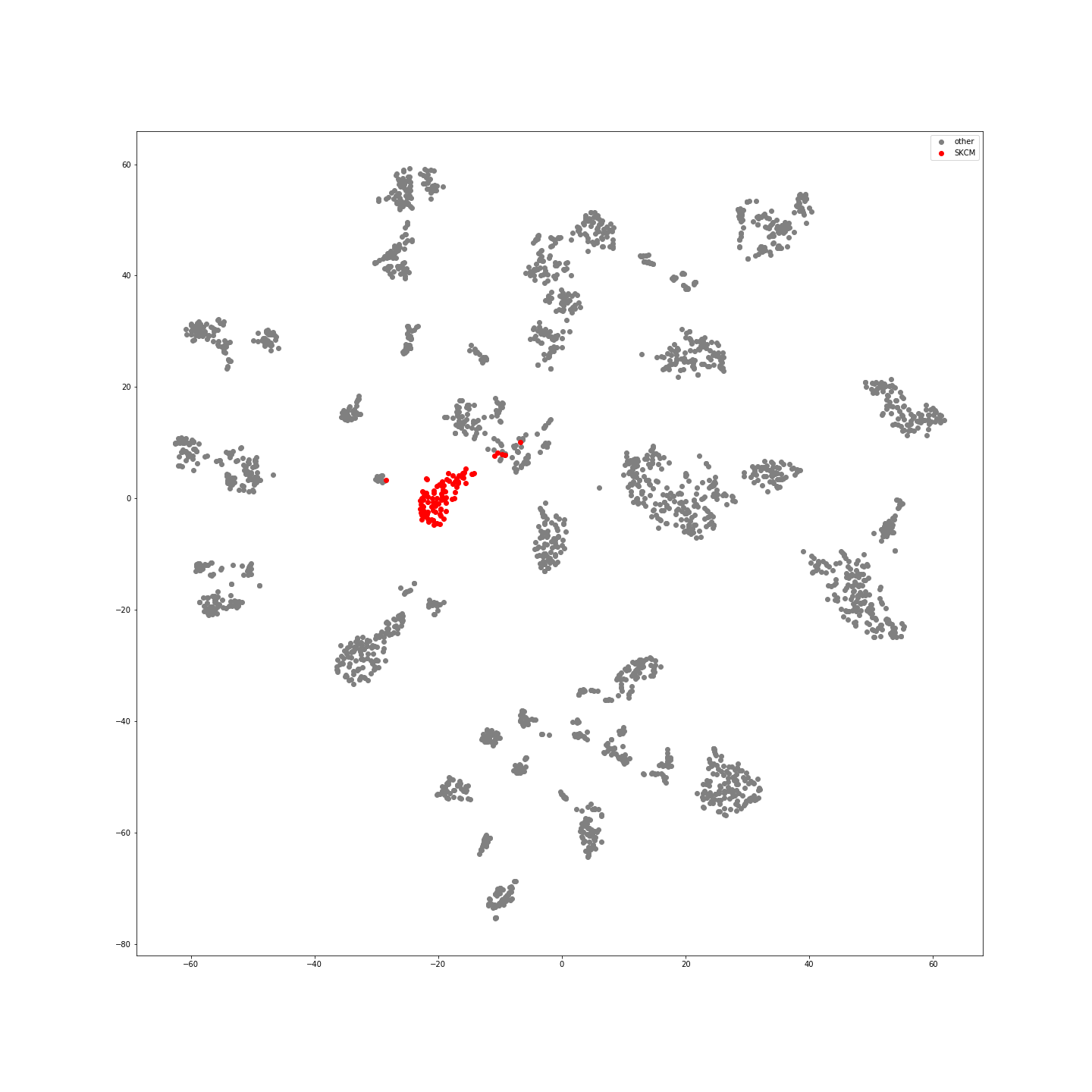


Supplementary Figure 55: SKCM Samples in Latent Space


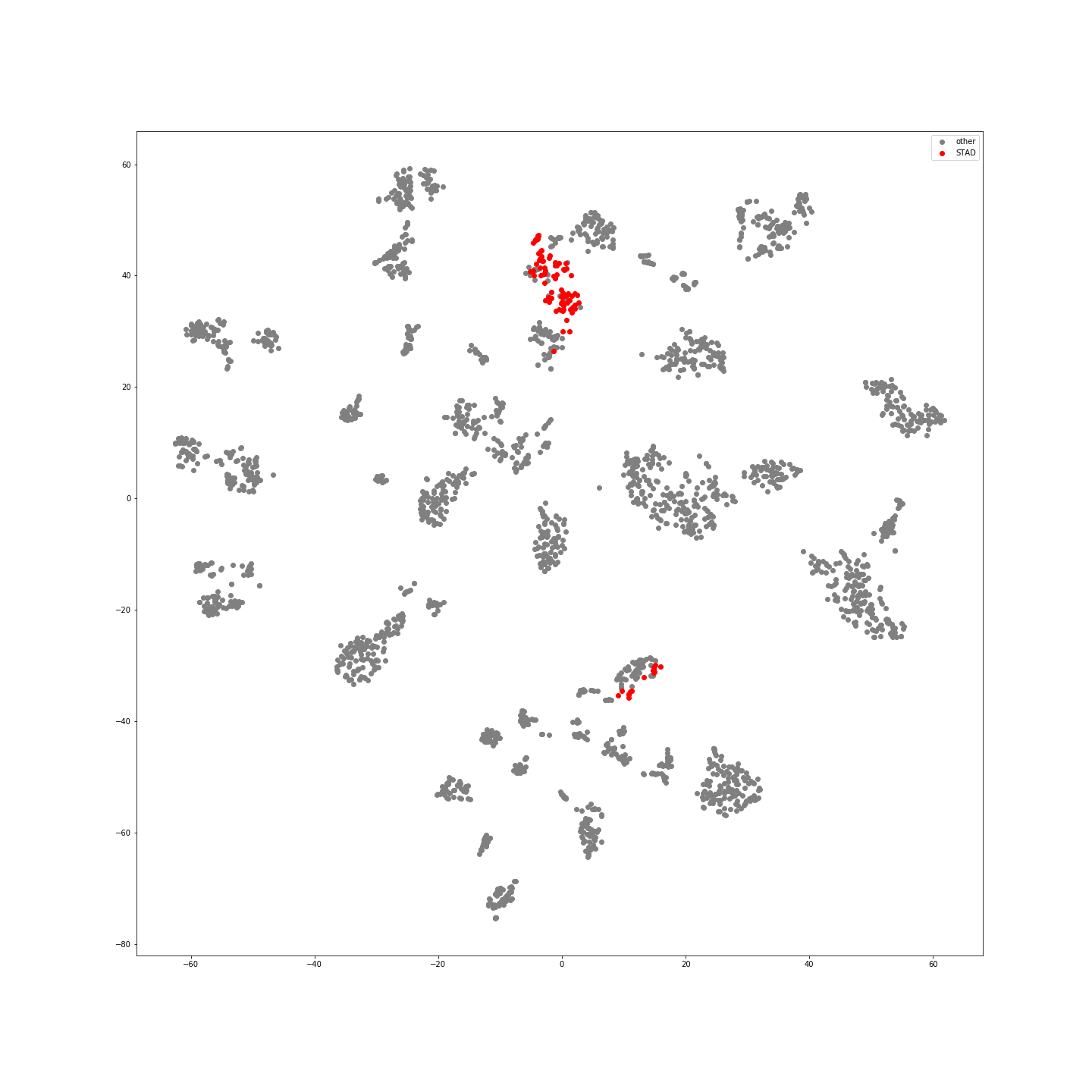


Supplementary Figure 56: STAD Samples in Latent Space


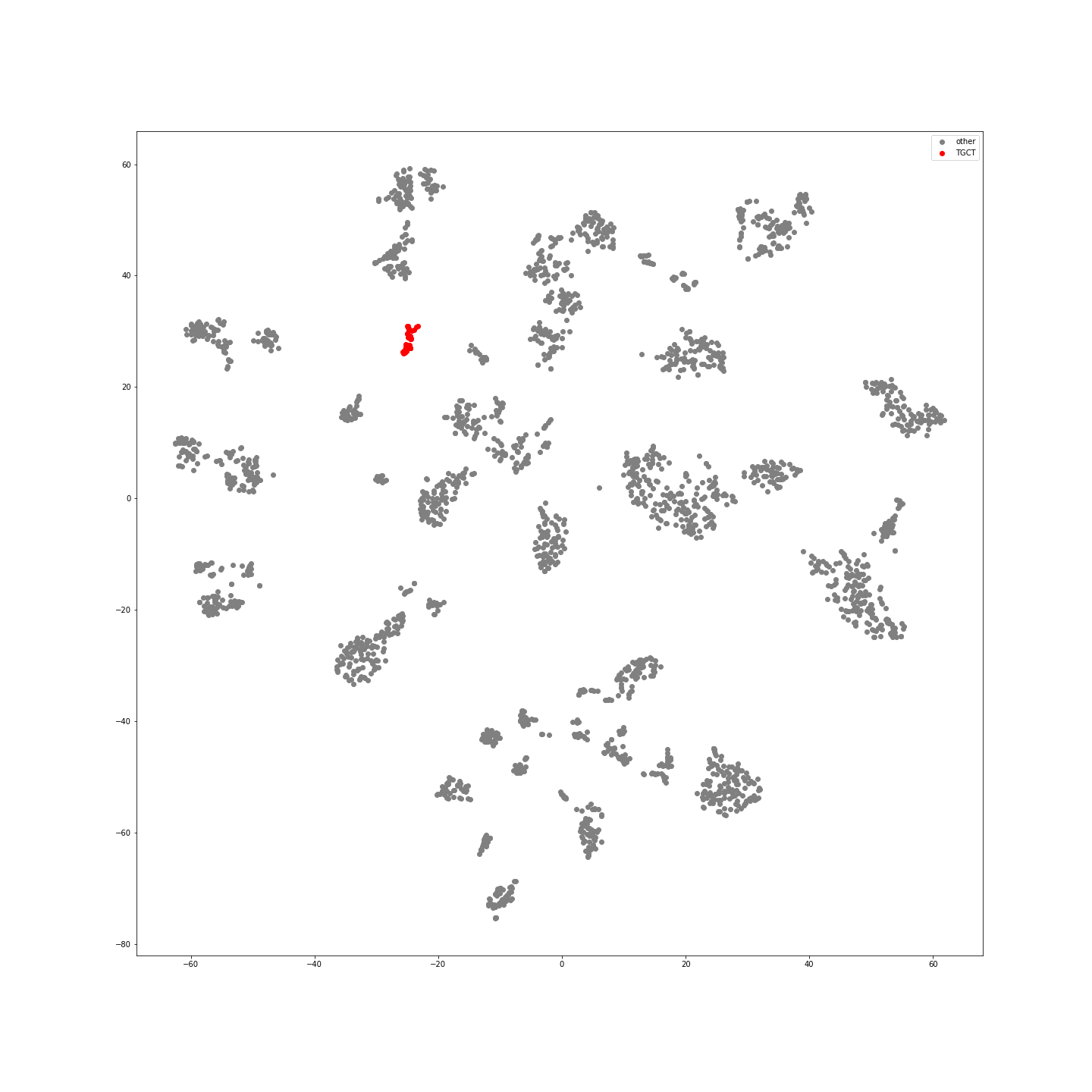


Supplementary Figure 57:TGCT Samples in Latent Space


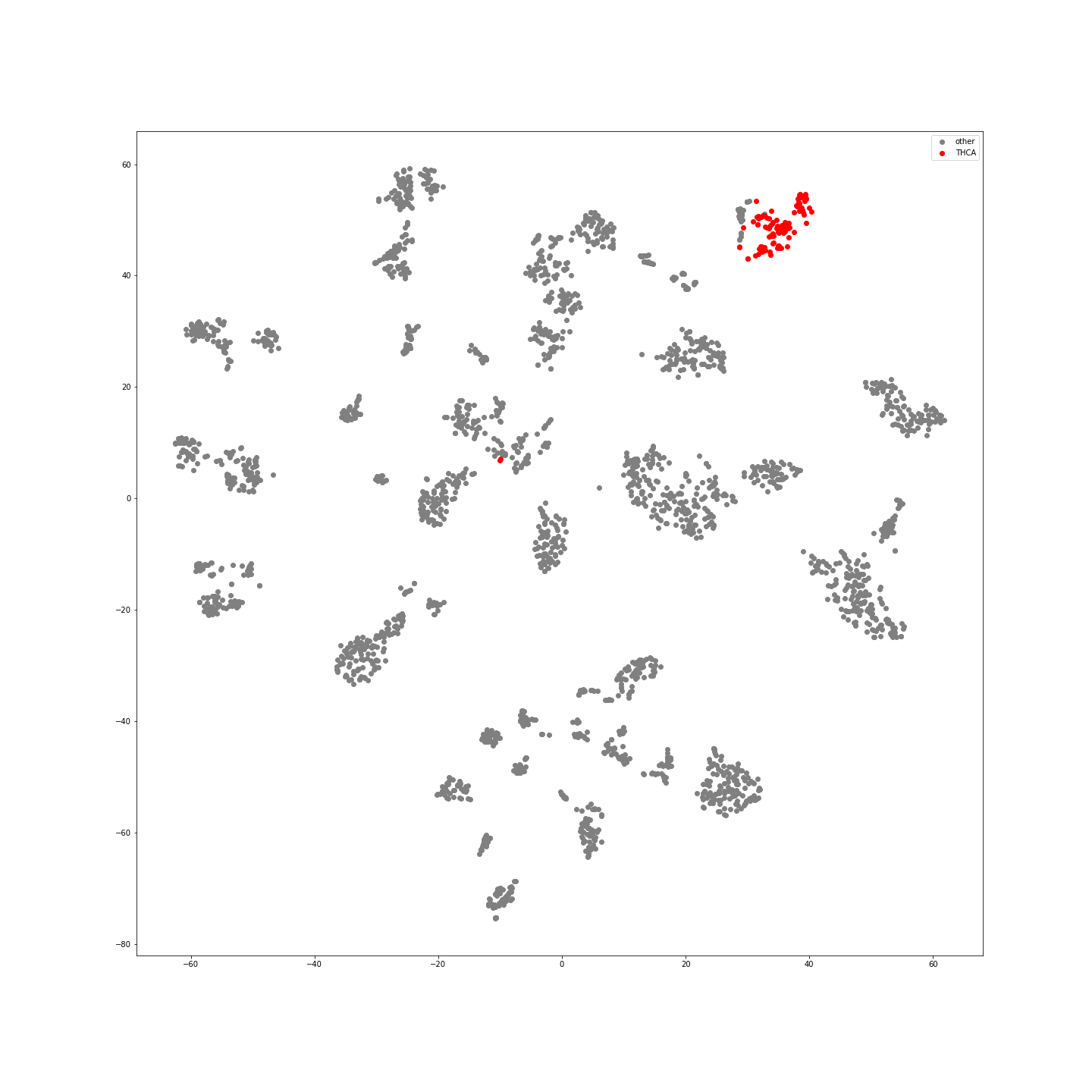


Supplementary Figure 58:THCA Samples in Latent Space


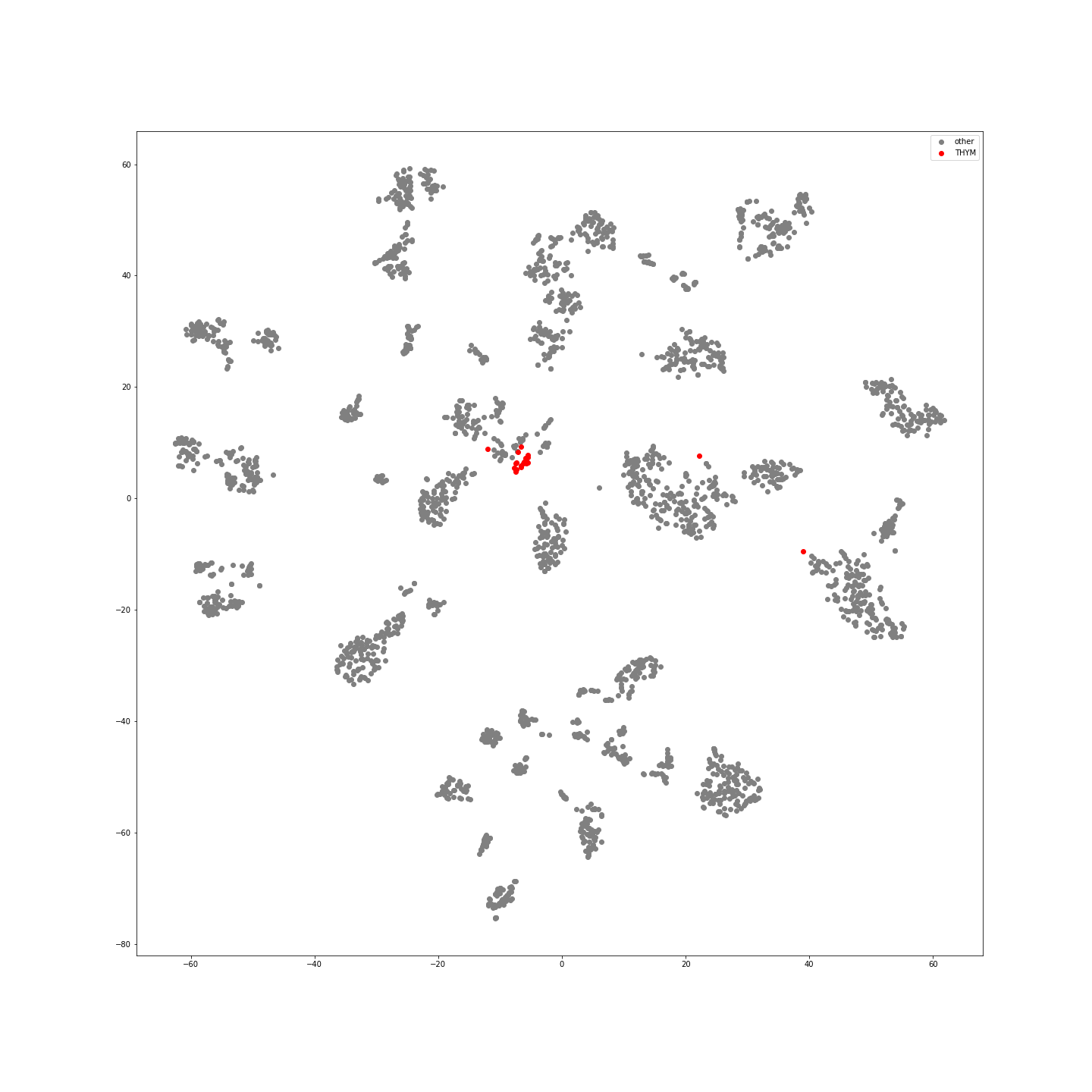


Supplementary Figure 59:THYM Samples in Latent Space


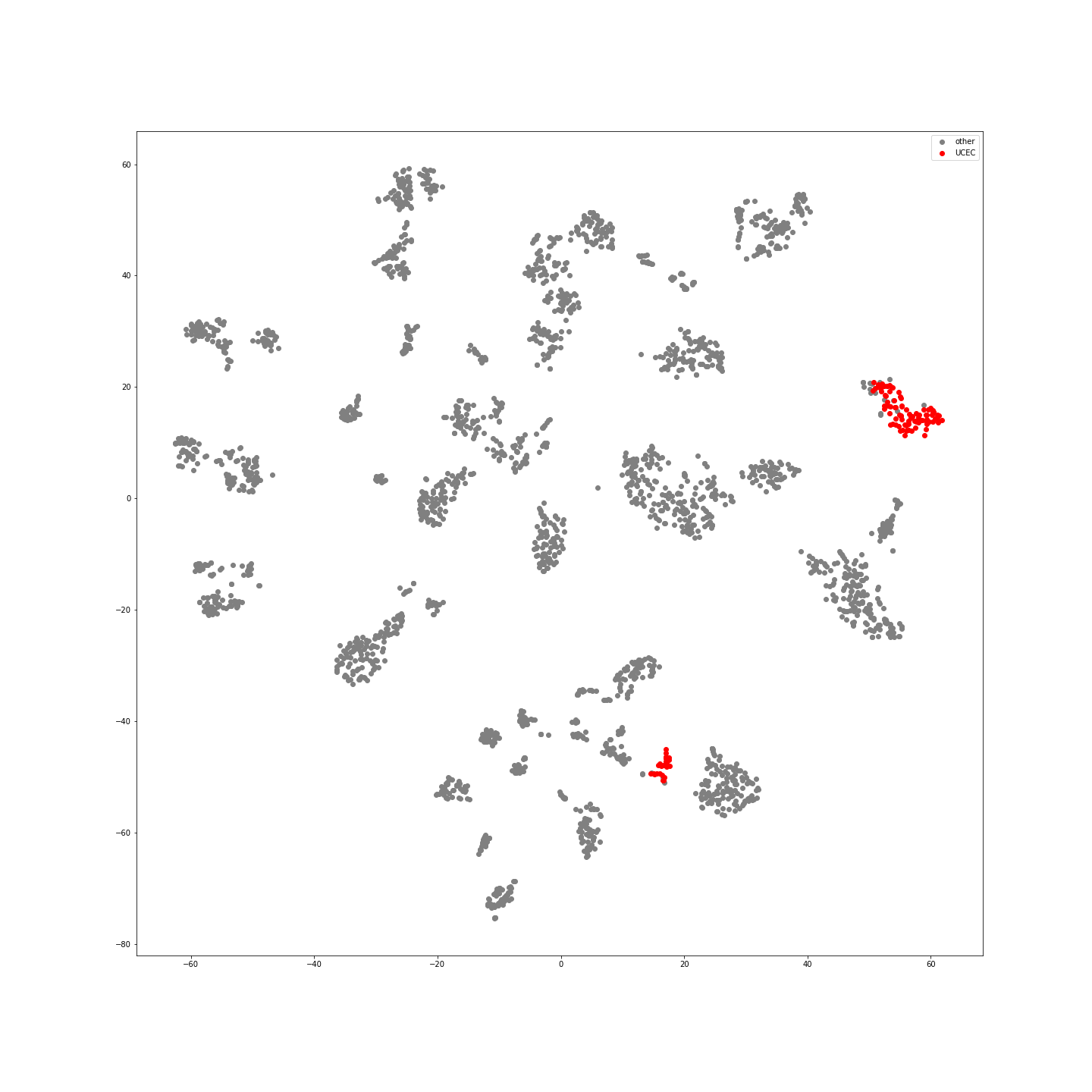


Supplementary Figure 60:UCEC Samples in Latent Space


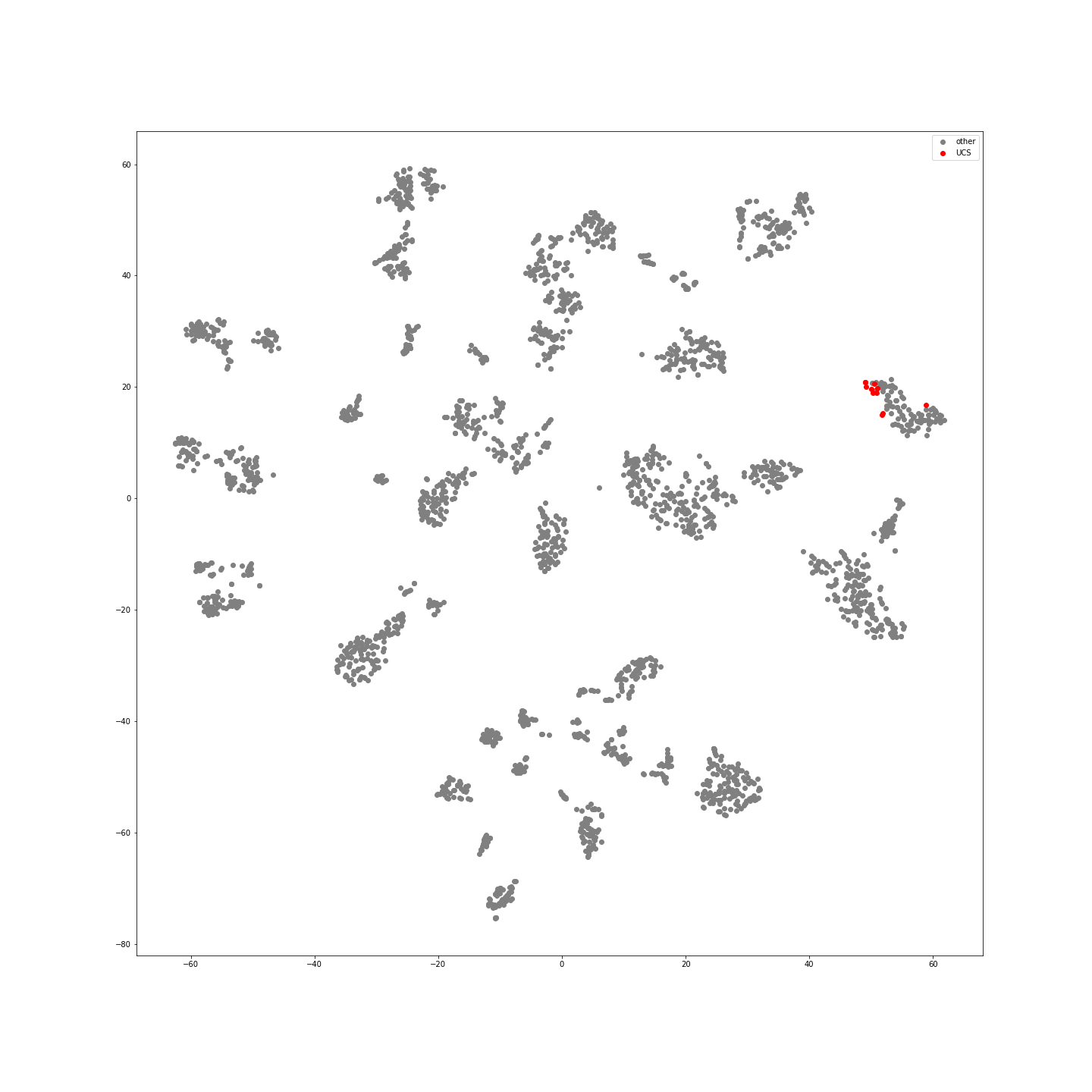


Supplementary Figure 61: UCS Samples in Latent Space


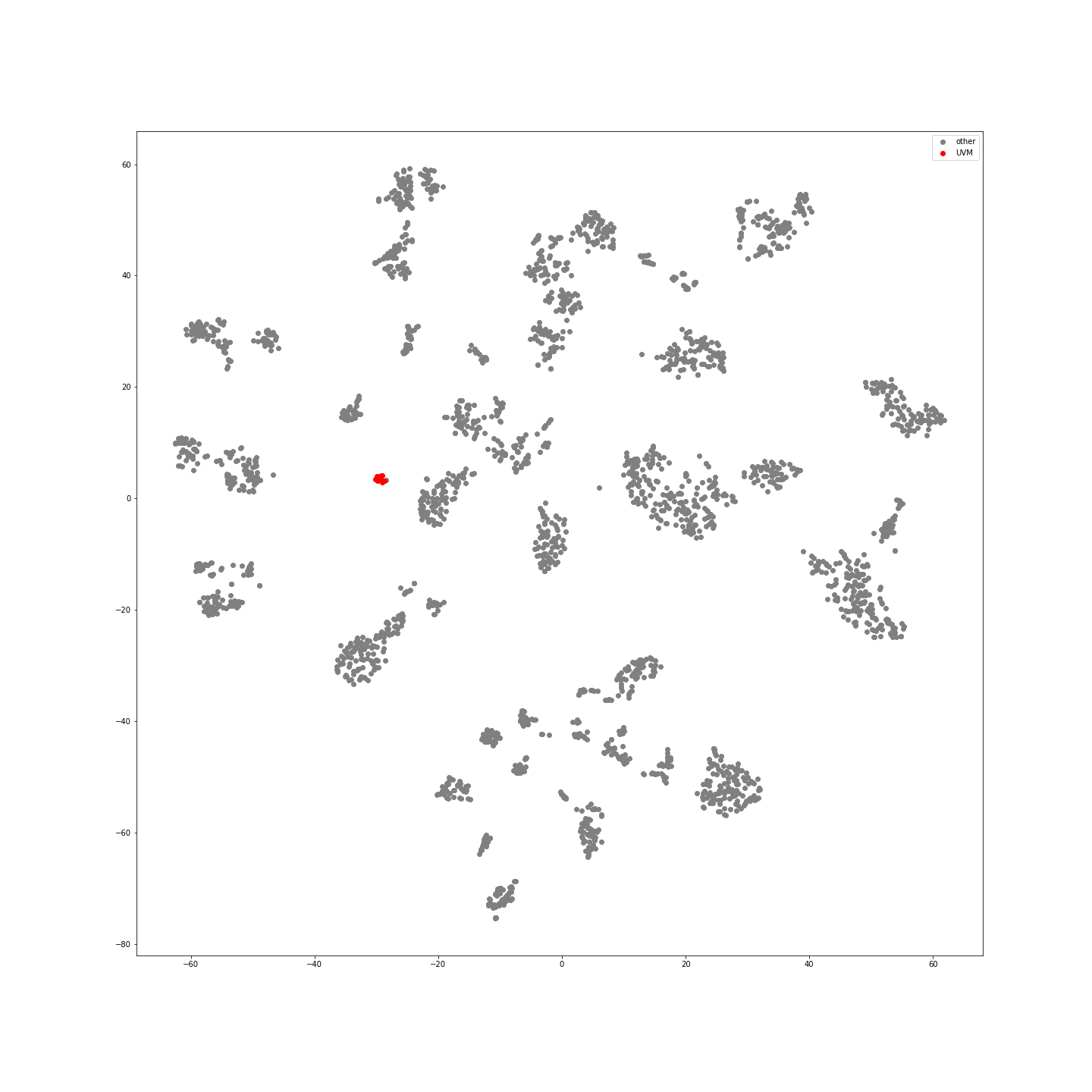


Supplementary Figure 62: UVM Samples in Latent Space

Supplementary Figure 63: Latent space arrangement of samples colored by predicted class


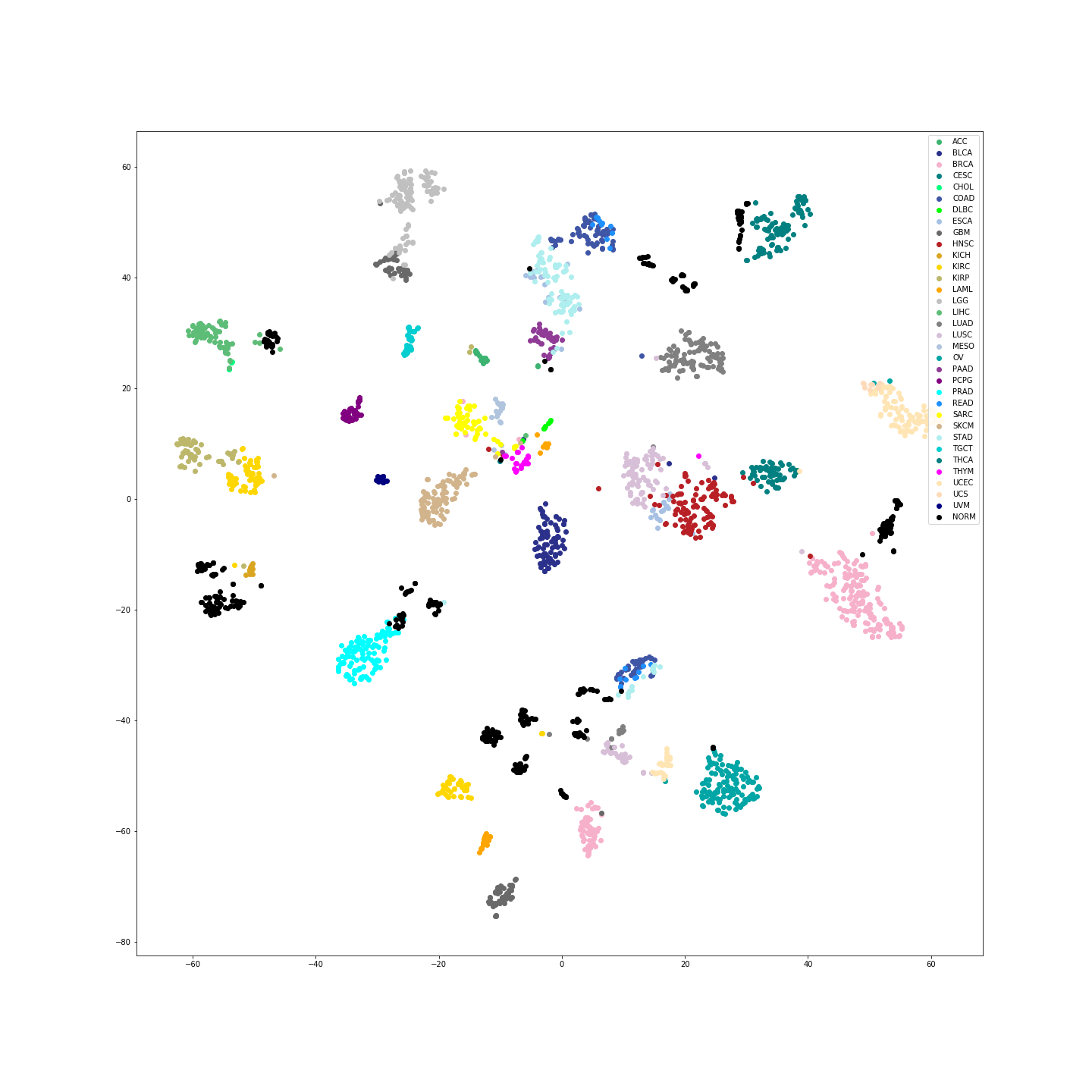


Supplementary Figure 63: All Samples in Latent Space
